# Consumer exposure to biocides - identification of relevant sources and evaluation of possible health effects

**DOI:** 10.1186/1476-069X-9-7

**Published:** 2010-02-03

**Authors:** Stefan Hahn, Klaus Schneider, Stefan Gartiser, Wolfgang Heger, Inge Mangelsdorf

**Affiliations:** 1 Fraunhofer Institute for Toxicology and Experimental Medicine, Department Chemical Risk Assessment, Nikolai-Fuchs-Str. 1, 30625 Hanover, Germany; 2 Research and Advisory Institute for Hazardous Substances GmbH (FoBiG), Klarastraße 63, 79106 Freiburg, Germany; 3 Hydrotox GmbH, Bötzinger Str. 29, 79111 Freiburg, Germany; 4 German Federal Environmental Agency, Corrensplatz 1, 14195 Berlin, Germany

## Abstract

**Background:**

Products containing biocides are used for a variety of purposes in the home environment. To assess potential health risks, data on products containing biocides were gathered by means of a market survey, exposures were estimated using a worst case scenario approach (screening), the hazard of the active components were evaluated, and a preliminary risk assessment was conducted.

**Methods:**

Information on biocide-containing products was collected by on-site research, by an internet inquiry as well as research into databases and lists of active substances. Twenty active substances were selected for detailed investigation. The products containing these substances were subsequently classified by range of application; typical concentrations were derived. Potential exposures were then estimated using a worst case scenario approach according to the European Commission's Technical Guidance Document on Risk Assessment. Relevant combinations of scenarios and active substances were identified. The toxicological data for these substances were compiled in substance dossiers. For estimating risks, the margins of exposure (MOEs) were determined.

**Results:**

Numerous consumer products were found to contain biocides. However, it appeared that only a limited number of biocidal active substances or groups of biocidal active substances were being used. The lowest MOEs for dermal exposure or exposure by inhalation were obtained for the following scenarios and biocides: indoor pest control using sprays, stickers or evaporators (chlorpyrifos, dichlorvos) and spraying of disinfectants as well as cleaning of surfaces with concentrates (hydrogen peroxide, formaldehyde, glutardialdehyde). The risk from aggregate exposure to individual biocides via different exposure scenarios was higher than the highest single exposure on average by a factor of three. From the 20 biocides assessed 10 had skin-sensitizing properties. The biocides isothiazolinone (mixture of 5-chloro-2-methyl-2H-isothiazolin-3-one and 2-methyl-2H-isothiazolin-3-one, CMI/MI), glutardialdehyde, formaldehyde and chloroacetamide may be present in household products in concentrations which have induced sensitization in experimental studies.

**Conclusions:**

Exposure to biocides from household products may contribute to induction of sensitization in the population. The use of biocides in consumer products should be carefully evaluated. Detailed risk assessments will become available within the framework of the EU Biocides Directive.

## Background

In many areas of our daily life, biocidal active substances are used to ensure personal hygiene, disinfect surfaces, control insects and preserve a wide range of non-durable goods. For some application areas, special antibacterial products (e.g., detergents, textile products, toilet seats) are available for everyday use. More than 200 biocides (active ingredients) have been notified within the scope of the Biocidal Products Directive for product type 1, 2 and 6 (human hygiene or private and public health area or in-can preservation) and more than 100 active ingredients for product type 18 and 19 (insecticides or repellents). However, some of the notified substances were not further supported by submitting a complete dossiers (e.g., chlorpyrifos, phoxim), so that the actually available number of biocidal active substances is expected to decrease. Human exposure may occur by inhalation from spraying of products containing biocides or from biocides evaporating from the products. Furthermore, biocides may come into contact with the skin during use of products containing biocides. Oral exposure is assumed to play a minor role with the exception of mouthing of contaminated objects by children.

Despite the wide uses and possible exposure, little is known about exposure concentrations and no systematic measurements are available for the home environment. Measurements have been taken primarily at workplaces, for example to determine exposure to formaldehyde during disinfection in hospitals [1,2], or at workplaces during spraying of biocides [3]. Apart from these studies, few measurements on consumer exposures are available, for example, on the emission of biocides from emulsion paints [4-6], or on the exposure to insecticides such as dichlorvos or chlorpyrifos [7-9]. Relevant for consumer exposure are also investigations on biocide emission from carpets [10].

As an alternative to measurements, exposures can be estimated using appropriate models. Models estimating exposure from the use of household products, have been outlined in guidance documents issued by the European Commission for assessing exposure for chemicals and biocidal active substances [11,12], and also in the HERA Guidance Document [13]. Computer-based models are available to calculate these exposure data and to refine calculated worst case scenario exposure data (e.g., SCIES-CEM (implemented in E-Fast), ConsExpo [14], SprayExpo [15]). However, the database for input parameters into exposure calculation is weak. In particular typical concentrations in products, use patterns and data on product use are missing.

Therefore the relevance of the biocides used in household products was investigated in a project sponsored by the German Federal Agency on the Environment (project period 15.10.2004 - 30.11.2005). In this project, first the content of biocides in consumer products was investigated. Then, the overall exposure to individual biocidal active substances from consumer products was modelled using a worst case scenario approach. Finally, the toxicological data were evaluated, and the potential health risks were assessed. For more detailed information see the final report of the project [16].

## Methods

### Data collection

The following consumer products were chosen: disinfectants, wood preservatives and insecticides, repellents and attractants, as well as preservatives in preparations (e.g. in washing and cleaning products, in cosmetics, in home improvement products, etc.). Information on the identity of active substances, their concentration and the application of use of these biocidal products, was collected in supermarkets and do-it-yourself stores ("on site market research"). The obligation to label biocidal products according to Article 20 of the European Biocidal Products Directive facilitated this task, as practically all information required was printed on the retail package. Additional information was obtained from inquiries to industry and via the Internet. Furthermore, databases and lists of active substances were analysed, such as the register of detergents and cleaning agents of the German Federal Environmental Agency, which provided (product-independent) summary analyses, and the German drug directory "Rote Liste".

In addition to biocides used indoors in private homes, other sources of exposure to biocidal substances were taken into account. These included, for example, preservatives in cosmetics, human and veterinary drugs, and antimicrobially-finished articles which do not (or only partly) fall under the Biocidal Products Directive. The typical concentrations of preservatives in consumer products were obtained by researching the Internet and by direct inquiries to manufacturing companies. For cosmetics, no information on the preservatives was available for individual products. Therefore, the maximum allowed concentrations according to the Cosmetics Directive [17] were used for the exposure assessment. Finally, some information could be obtained from the relevant literature.

The information on products and active substances has been compiled in so called product tables (containing information on each individual product identified, its biocidal ingredients and concentrations as well as instructions for use) and active substance (biocide) tables which summarized data obtained from the product tables. The individual products have then been assigned to product categories, product groups and typical products or exposure scenarios. Whenever gaps became apparent in the information on scenarios and products, the research was systematically refined (iterative process).

### Selection of biocides

Twenty active substances were selected for detailed investigation. The most important selection criteria were the application quantity (as far as known), a wide range of applications in the household and the personal home environment (e.g. as disinfectants and preservatives in household products, in handicraft materials, in cosmetics), and the use in a variety of household products. Furthermore, the selected active substances should be stated as notified in the Second Review Regulation of the Biocidal Products Directive to guarantee that they will be on the market in future. Active substances of low toxicity, such as citrate or sodium hydrogen carbonate, were excluded. Individual active substances intended for special application scenarios, e.g. mosquito and ant repellents, were additionally taken into account.

Products containing the selected active substances were categorized by types of applications. Then commonly used concentrations of the active ingredients for each type of application were derived.

### Exposure modelling

The algorithms proposed in the Technical Guidance Document on Risk Assessment (TGD) [11] were used for the estimation of the exposure (for screening purposes only). In the case of exposure to compounds by evaporation, the exposure concentration was limited to the maximum amount according to the ideal gas law. In addition, the fugacity was introduced to describe the distribution between liquids and gases. This was important for formaldehyde, which despite its high vapour pressure does not quickly evaporate from solutions due to its high water solubility (low Henry coefficient). Default values were taken from the TGD, ConsExpo 4.0, the HERA guidance document [13], the US EPA Exposure factors handbook [18] and by expert guess. Additionally, specific data were taken from product information (via internet and market research). Dermal and inhalation exposure were assessed for most of the exposure scenarios. The oral route has only been taken into consideration for exposure in a few scenarios e.g. via swallowing disinfected swimming pool water, via intake of residues of dishwashing products on dishes, as well as via intake of cosmetics (toothpaste, mouth wash, lip sticks). Exposure of children by mouthing of dust possibly contaminated with biocides has not been taken into consideration. The exposure calculated as body dose for the different routes (inhalation, dermal, oral) was summed up; in doing so the total exposure value for each scenario has been limited by using the maximum amount used. Then, the exposure from different sources of the same biocide were summed up to give the overall exposure. For details on the exposure modelling cf. Hahn et al. [16] or additional file 1: Printout of the spreadsheet used for exposure calculation (includes used assumptions and default values per scenario).

### Determination of Hazard and Risk

The toxicological data for the substances were analysed on the basis of reviews, supplemented by original literature, and summarised and evaluated with respect to the exposure data (for details cf. Hahn et al. [16]).

To assess the systemic effects of biocide exposure, MOEs (margins of exposure), i.e. the ratio of NOAEL (No Observed Adverse Effect Level) and exposure concentration (as body dose), were calculated. MOEs were calculated for the individual scenarios and for the total of all exposures from all scenarios.

**Figure 1:**
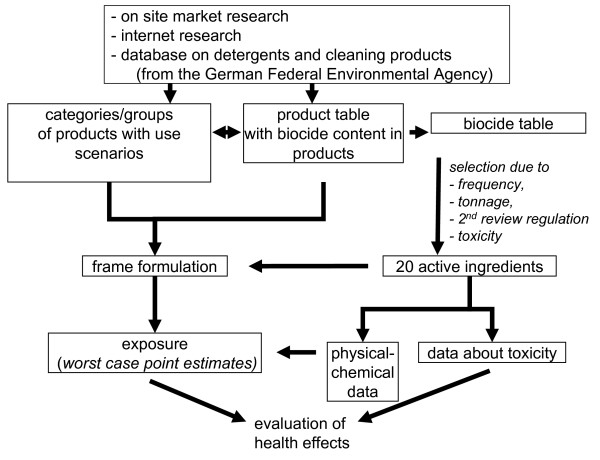
**Approach selected for screening potential health risks from biocide-containing products of daily use**.

Figure 1 schematically shows the selected approach in detail.

## Results

### Market research

Numerous consumer products have been identified, which contain biocides such as surface disinfectants, laundry disinfectants, insecticides and repellents. In addition, a major use of biocidal active substances is as a preservative in, for example, washing and cleaning products, cosmetics, and home improvement products such as paints.

Although more than 200 active ingredients have been notified within the scope of the Biocidal Products Directive for applications like disinfection (for human hygiene or private and public health area) or in can preservation and more than 100 active ingredients as insecticides or repellents, our market research has shown that only a limited number of active substances are used in most of the products [16]. In addition, the active substances can be subsumed by chemical groups such as formaldehyde and formaldehyde releasers, hydrogen peroxide and hydrogen peroxide releasers, pyrethroids, organophosphates, alcohols.

The market research revealed the following as main application areas of biocidal substances in washing and cleaning products, whereas the intended effect is not always primarily biocidal but possibly bleaching or cleaning:

a) surface disinfection (inclusive removal of moulds and films) using sodium hypochlorite (NaOCl), alcohols, quaternary ammonium compounds (QAC) and hydrogen peroxide;

b) laundry disinfection/cleaning clothes using hydrogen peroxide, NaOCl and QAC;

c) machine dishwashing products using dichloroisocyanurates and trichloroisocyanuric acid;

d) water purification in private swimming pools using dichloroisocyanurates, trichloroisocyanuric acid, sodium hypochlorite and hydrogen peroxide as the main biocidal active substances.

Preservatives used in liquid washing and cleaning products include isothiazolinones, benzoic acid, 2-phenoxyethanol, chloroacetamide, bronopol and triclosan. QAC, glutardialdehyde and formaldehyde or formaldehyde releasers are used as disinfectants and preservatives, too. Substances prevailing in the preservation of cosmetics are 2-phenoxyethanol, hydroxybenzoates (parabens), isothiazolinones and bronopol. The concentration range can be estimated fairly well based on the maximum concentration of active ingredients stipulated in the Cosmetics Directive. Frequently, cosmetics contain more than one biocidal active substance, e.g. groups of compounds such as several isothiazolinones, or several parabens or combinations of differently acting biocides such as isothiazilinone and formaldehyde-releasing agents. Our market research showed, furthermore, that the use of preservatives in toys such as finger paints or plasticine also follows the Cosmetics Directive. The most relevant active substances used for in-can preservation were identified as isothiazolinones, bronopol and formaldehyde releasing agents, while specific fungicides and herbicides such as triazines and carbamates are used for film preservation.

Household insecticides and repellents are used in the form of bait boxes, strips/stickers, powders or liquid preparations to control crawling insects and in the form of sprays and evaporators to control flying insects. The most commonly used active substances belong to the categories of pyrethroids (e.g., tetramethrin, allethrin, prallethrin, etc.) and organophosphates (e.g., chlorpyrifos, dichlorvos, phoxim, etc.). Among the repellents, the most relevant active substances were found to be icaridin, ethyl 3-(N-butylacetamido)propionate and diethyltoluamide.

To control ectoparasites on pets, mainly pyrethroids are used (e.g. in impregnated collars, sprays, powders and shampoos). As further sources of biocides in household-related areas, we identified antimicrobially-finished textile materials (in particular sportswear, carpets and mattresses). Here, mainly zinc pyrithione, carbendazim, different isothiazolinones, permethrin and triclosan are used as storage preservatives, as moth repellents and for odor control. No statement can be made at present about the relevance of other antimicrobially-finished articles (such as antimicrobial bin liners, toilet seats and carving boards) for the overall exposure. The active substances used in this domain, are normally integrated directly into the polymers. No meaningful data was found.

### Exposure assessment

Within this project, 20 active substances were selected for detailed investigation, 15 of which are used as disinfectants and preservatives and five as insecticides and repellents (Table 1). Products containing the selected active substances were classified by range of application. This resulted in a total of 50 scenarios (see Table S1; additional file 2) and about 220 formulations (i.e. combinations of commonly used concentrations of the active ingredients with the 50 scenarios). For all these formulations the exposure for the consumer was estimated using the algorithms of the TGD with some modifications (limited to maximal possible concentration in air using ideal gas law and fugacity, limited to maximal amount used). These estimations of the potential exposure are thus worst case scenario approaches intended for screening purposes. A refinement would probably result in considerably lower and more realistic values.

**Table 1:** Selected biocidal active ingredient attributed to field of application

CAS	EC	Name	Disinfectants	Preservatives	Insecticides, repellents
67-63-0	200-661-7	2-Propanol	x		

68391-01-5	269-919-4	Alkyl dimethyl benzyl ammonium chlorides (QAC)	x	x	

7681-52-9	231-668-3	Sodium hypochlorite	x		

2893-78-9	220-767-7	Sodium dichloroisocyanurate	x		

87-90-1	201-782-8	Trichloroisocyanuric acid	x		

7722-84-1	231-765-0	Hydrogen peroxide	x		

3380-34-5	222-182-2	Triclosan	x	x	

50-00-0	200-001-8	Formaldehyde	x	x	

111-30-8	203-856-5	Glutardialdehyde	x	x	

65-85-0	200-618-2	Benzoic acid	x	x	

55965-84-9	mixture	mixture of 5-chloro-2-methyl-2H-isothiazolin-3-one and 2-methyl-2H-isothiazolin-3-one (CMI/MI)	x	x	

2634-33-5	220-120-9	1,2-Benzisothiazolin-3-one	x	x	

52-51-7	200-143-0	Bronopol	x	x	

122-99-6	204-589-7	2-Phenoxyethanol	x	x	

79-07-2	201-174-2	Chloroacetamide	x	x	

119515-38-7	423-210-8	Icaridin			x

23031-36-9	245-387-9	Prallethrin			x

2921-88-2	220-864-4	Chlorpyrifos			x

62-73-7	200-547-7	Dichlorvos			x

14816-18-3	238-887-3	Phoxim			x

The results of the exposure calculation are given in Table 2 for oral exposure, exposure by inhalation and dermal exposure. In addition, the exposure from the three routes was summarized to result in an overall exposure.

**Table 2:** Worst case scenario exposure estimates (total of all scenarios)

Biocide	Exposure in mg/kg/d			
	Inhalation ^1^	Dermal ^1^	Oral ^1^	Total ^1, 2^
2-Propanol	82	382	0	387

Alkyl dimethyl benzyl ammonium chloride (QAC)	0.75	42	0.0010	43

Sodium hypochlorite	0.57	76	0.16	76

Sodium dichloroisocyanurate	3.7E-12	5.7	0.0027	5.8

Trichloroisocyanuric acid	4.3E-6	6.0	0.0030	6.0

Hydrogen peroxide	15	127	0.013	134

Triclosan	0.084	2.6	0.011	2.6

Formaldehyde	3.7	2.8	0.085	5.2

Glutardialdehyde	2.5	6.0	0.00025	8.1

Benzoic acid	3.1	4.3	0.0050	4.4

CMI/MI	0.17	0.15	0.000018	0.30

1,2-Benzisothiazolin-3-one	0.058	3.3	0.00013	3.3

Bronopol	0.22	1.9	0.0010	2.0

2-Phenoxyethanol	17	23	0.011	30

Chloroacetamide	4.3	4.6	0.0032	6.9

Icaridin	0.66	6.9	0	6.9

Prallethrin	0.012	0.039	0	0.051

Chlorpyrifos	0.61	2.2	0	2.8

Dichlorvos	7.0	0.096	0	7.0

Phoxim	0.059	1.8	0	1.9

CMI/MI = mixture of 5-chloro-2-methyl-2H-isothiazolin-3-one and 2-methyl-2H-isothiazolin-3-one

1 = value is an aggregate value of different scenarios (max 24 of 50)

2 = each scenario is limited to maximal amount used and then all scenarios are summed up

Roughly half of the selected biocidal active substances have a relatively low vapour pressure and/or a low Henry coefficient, so that inhalation exposure via the gas phase is negligible in most cases. Exceptions are dichlorvos and formaldehyde, which could evaporate from insecticide strips and home improvement products, respectively. In contrast, there can be high inhalation exposure through aerosols during spray application. This includes biocides like hydrogen peroxide, glutardialdehyde, formaldehyde, chlorpyrifos.

Looking at preservatives, there is a wide range of applications and at the same time a limited number of active substances used (e.g., in cleaning products, in cosmetics, in home improvement products, etc.), so that the aggregate exposure from all products is of importance. In addition, a high potential exposure (both dermal and inhalation) is possible in particular with leave-on cosmetics.

### Characterisation of biocide toxicity

Except for the two substances sodium dichloroisocyanurate and trichloroisocyanuric acid, there is sufficient data for all selected biocidal active substances available to characterise the systemic toxicity after long-term exposure, at least for oral exposure, although many of the relevant studies have not been published and are described only in secondary reports.

The profile of toxic effects, in agreement with the different chemical classes represented, varies broadly both in terms of type of effects and of potency [16]. For example, the insecticides belonging to the group of organophosphates (chlorpyrifos, dichlorvos, phoxim) show the known effects on cholinesterases in blood and in the brain, whereas for substances such as hydrogen peroxide, formaldehyde and glutardialdehyde, irritant effects are most prominent. NOAEL values after subchronic or chronic oral exposure vary across four orders of magnitude: The lowest value is 0.025 (chlorpyrifos), the highest is 500 mg/kg/d (benzoic acid). As expected, the toxicity of pest control agents is substantially higher than that of disinfectants or preservatives. The relevant effects of the substances and, as far as possible, the NOAELs (no observed adverse effect levels) for the oral, inhalational and dermal routes of intake were summarised (see Table S2; additional file 3).

In addition to systemic effects, irritant effects on the skin and mucous membranes deserve special attention. For 11 out of 20 substances, relevant irritation of the skin and mucous membranes was found: sodium hypochlorite, alkyl dimethyl benzyl ammonium chloride (QAC), isothiazolinones (mixture of 5-chloro-2-methyl-2H-isothiazolin-3-one and 2-methyl-2H-isothiazolin-3-one (CMI/MI), 1,2-benzisothiazolin-3-one), glutardialdehyde and formaldehyde, di- and trichloroisocyanurates, hydrogen peroxide, bronopol and triclosan belong to this group. Regarding irritation, an additive effect must be assumed if different products containing these active substances are used in parallel or consecutively within a short time period. Irritation effects in the respiratory tract may be relevant for inhalation exposure during spraying. This holds true in particular for formaldehyde and glutardialdehyde.

Furthermore, for 10 of the active substances investigated, there is evidence that they may possess skin-sensitising properties. These are the isothiazolinones (CMI/MI and 1,2-benzisothiazolin-3-one), glutardialdehyde and formaldehyde, chloroacetamide, dichlorvos, phoxim, bronopol, triclosan as well as sodium hypochlorite. No data are available for skin-sensitising effects of di- and trichloroisocyanurates [16]. The substantial importance of dermal contact with objects of daily use, underline the relevance of this endpoint. For some substances, the concentrations which induced sensitisation in humans or in the local lymph node assay in mice were compared to the application concentrations in products of daily use (Table 3). With the exception of bronopol, there seem to be only minor differences between the effect concentrations and application concentrations indicating that CMI/MI, gluardialdehyde, formaldehyde and chloracetamide in household products may induce sensitization.

**Table 3:** Comparison of concentrations with sensitising effects in human (HRIPT) or in mice (LLNA) to the estimated concentrations of application in products of daily use

Active substance	Sensitising concentration in human (HRIPT)	Sensitising concentration in animal testing (LLNA)	Concentration of application		
			Cleaning & washing	Cosmetics	Home improvement
CMI/MI	12.5 ppm [26](challenge conc. 12.5 ppm)	75 ppm (EC_3_) [27]	≤ 60 ppm	2.5 - 7.5 ppm (15 ppm^a^)	

Glutardialdehyde	5% [28](challenge conc. 0.5%)	0.1% (EC_3_) [29]	0.01 - 0.5%	≤ 0.1% (0.1%^a^)	

Formaldehyde	1% [28](challenge conc. 1%)	0.35% (EC_3_) [30]	≤ 0.05%	≤ 0.2% (0.2%^a^)	≤ 0.1%

Bronopol	5% [28](challenge conc. 2.5%)		0.0011 - 0.035%	0.01 - 0.1% (0.1%^a^)	0.0011 - 0.035%

Chloroacetamide	0.5% [31](challenge conc. 0.5%)		0.1 - 0.3%	≤ 0.3% (0.3%^a^)	0.1 - 0.3%

CMI/MI = mixture of 5-chloro-2-methyl-2H-isothiazolin-3-one and 2-methyl-2H-isothiazolin-3-oneHRIPT: Human Repeat Insult Patch test

LLNA: Local lymph node assay

EC_3_: concentration leading to a triplicate proliferation rate in the Local lymph node assay.

a = max. value of cosmetics directive [17]

### Identification of potential risks

Comparison of the modelled exposure concentrations with the NOAELs enabled calculation of a margin of exposure (MOE) as a preliminary indication of potential risk. For MOEs > 1 the exposure concentration is lower than the NOAEL, for MOEs < 1 the exposure concentration is higher than the NOAEL, i.e. the higher the MOE, the lower the potential risk. These MOEs can be used for relative comparison between the biocidal substances. Overall MOEs (Table 4) summed up for each individual compound over all exposure scenarios and all routes of exposure ranged from 57 for benzoic acid to 0.0057 for dichlorvos. In all cases, oral exposure has only a minor share in the overall exposure. For the inhalation pathway, MOEs below one were obtained for dichlorvos, formaldehyde, hydrogen peroxide, glutardialdehyde, CMI/MI and chlorpyrifos. The low MOEs are in general dominated by only a small number of scenarios: pest control using sprays (chlorpyrifos), stickers or evaporators (dichlorvos), spraying of disinfectants or cleaning of surfaces with concentrates (glutardialdehyde and hydrogen peroxide), application of water-based paints and adhesives (formaldehyde, CMI/MI), and use as preservative in personal hygiene products (formaldehyde). For the dermal pathway, MOEs below one were calculated for sodium hypochlorite, hydrogen peroxide, alkyl dimethyl benzyl ammonium chloride (QAC), 2-propanol and phoxim. In this context, similar to inhalation exposure, the focus for sodium hypochlorite, hydrogen peroxide and 2-propanol was on scenarios in which the biocidal substances are applied by spraying or in which concentrates are used.

**Table 4:** MOE based on aggregate exposure in adults (total of all scenarios)

active substance	MOE			
	Inhalation ^1^	Dermal ^1^	Oral ^1^	Total ^1, 2^
2-Propanol	1.8	0.26		0.26

Alkyl dimethyl benzyl ammonium chlorides (QAC)	33	0.24	25000	0.59

Sodium hypochlorite	5.2	0.040	18	0.040

Sodium dichloroisocyanurate	1.9e+10	8.7	18637	8.7

Trichloroisocyanuric acid	4122	8.4	16578	8.4

Hydrogen peroxide	0.0052	0.20	1950	0.19

Triclosan	298	31	2273	9.6

Formaldehyde	0.018	5.3	176	2.9

Glutardialdehyde	0.0043	8.3	15903	0.49

Benzoic acid	80	57	50000	57

CMI/MI	0.24	2.7	22200	1.3

1,2-Benzisothiazolin-3-one	144	2.6	66954	2.6

Bronopol	45	10	9827	4.9

2-Phenoxyethanol	4.7	21	7617	2.6

Chloroacetamide	2.3	7.7	3174	1.4

Icaridin	121	14		12

Prallethrin	6.7	761		49

Chlorpyrifos	0.058	2.3		0.011

Dichlorvos	0.00086	4.2		0.0057

Phoxim	6.4	0.27		0.20

CMI/MI = mixture of 5-chloro-2-methyl-2H-isothiazolin-3-one and 2-methyl-2H-isothiazolin-3-one

1 = value is an aggregate value of different scenarios (max 24 of 50) compared to respective NOAEL (inhalativ, dermal, oral)

2 = each scenario is limited to maximal amount used and then all scenarios are summed up. After that the value has been compared to the oral NOAEL

The influence of aggregate exposure on the MOE was assessed by comparing the MOE with the highest single exposure with the MOE for the exposure summed up for all scenarios for the same route of exposure [16]. Two to twenty-four scenarios were available per substance (average 11 scenarios). The highest aggregate effect was calculated for bronopol, the maximum factor here being 6.3 for inhalation and 8 for dermal intake, with a total of 24 scenarios. The average factor was 2.7 for inhalation and 3.1 for dermal exposure.

Comparing the MOEs for different biocides within one scenario, marked differences are observed between the different biocidal substances. The results for the airway-irritant biocides hydrogen peroxide, formaldehyde and glutardialdehyde give rise to concern regarding inhalation exposure to sprays (Table 5) and concentrates, MOEs being lowest for glutardialdehyde because of the particularly low NOAELs. Remarkable differences between MOEs for the different compounds also exist for the use of the substances as preservatives in cosmetics. This is of minor importance, however, for the assessment of the health risk, as all MOEs are very high both for inhalation and for dermal exposure. An exception is formaldehyde, which should, however, not be finally assessed until detailed exposure analyses have been carried out, since considerably lower and more realistic values can be achieved by refinement of the exposure assessment [16]. Regarding the use of insecticides as sprays for crack and crevice treatment, the MOE obtained for the organophosphate chlorpyrifos is substantially lower than that for the pyrethroid prallethrin (Table 6).

**Table 5:** MOE of different biocides for exposure scenario 8 (spraying and wiping, mould remover, algae remover, window cleaner)

active substance	MOE			
	inhalation	dermal	oral	total
*disinfection*				

2-Propanol	12	1.1	accidental	1.1

Hydrogen peroxide	0.012	0.52	accidental	0.52

Sodium hypochlorite	2.4e+12	12	accidental	12

Alkyl dimethyl benzyl ammonium chlorides (QAC)	320	1.3	accidental	3.3

*in-can preservative*				

CMI/MI	11	13	accidental	13

1,2-Benzisothiazolin-3-one	1018	11	accidental	11

Bronopol	2229	114	accidental	57

Formaldehyde	2.1	60	accidental	60

Glutardialdehyde	0.034	20	accidental	1.6

2-Phenoxyethanol	128	100	accidental	16

CMI/MI = mixture of 5-chloro-2-methyl-2H-isothiazolin-3-one and 2-methyl-2H-isothiazolin-3-one

**Table 6:** MOE of different biocides (insecticides) for exposure scenario 33c (sprays for crack and crevice, ant sprays)

Active substance	MOE			
	Inhalation	Dermal	Oral	Total
Chlorpyrifos	0.15	6.4	accidental	0.030

Prallethrin	6.8	770	accidental	49

## Discussion

As shown above, in this project the risk of different biocides in consumer products was assessed by modelling exposure concentrations and comparing these with NOAELs. The exposure was calculated using a worst case scenario approach by means of the algorithms given by the TGD with some modifications. Such worst case scenario approaches within exposure assessments are often used as a first tier for screening of risks and for comparison of different substances or exposure routes. However, due to the preliminary nature of the exposure assessment (screening), the estimated MOEs can be only a rough guide for assessing the health risks. It has to be taken into account that exposure levels obtained from the worst case scenario approach might be by a factor of 10-100 higher than the actual exposures [16,19]. Therefore, a refinement of the exposure values will actually be necessary. The exposure should be modelled with suitable tools such as ConsExpo [14] and SprayExpo [15], or even better, be measured. In cases, where sufficient information is available, probabilistic estimates might be used [20]. These refinements can then be subject to a detailed analysis of health risks.

Usually for defining safe levels in risk assessments, a margin of safety of 100 is considered appropriate [21]. However, a refinement of the exposure estimation may reduce the exposure by at least a factor of 10 to 100. Therefore only compounds and scenarios with MOEs < 1 have been highlighted in the results section. These involve mainly the indoor use of organophosphate pesticides. A refined exposure analysis including measured data from the literature for chlorpyrifos such as [9,22-24] and for dichlorvos such as [7] is necessary for assessing the risks from exposure to these organophosphates. To date, the application of chlorpyrifos for biocidal purposes is not allowed because industry did not submit a dossier for evaluation. Dichlorvos is being evaluated on a community level in the review programme of the Biocidal Product Directive. Other active substances may be considered for future use, as our comparative assessment showed that the exposure to prallethrin (a pyrethroid insecticide) resulted in a much higher MOE indicating a lower potential risk.

Another possible cause for concern was the use of sprays, which contain irritating compounds such as hydrogen peroxide, glutardialdehyde and formaldehyde. Also here a refined exposure analysis is of high priority as well as an analysis of particle size of the sprays.

Our analysis showed that the overall risk does not increase significantly if the exposure to a biocide from many uses is considered, because exposure is determined by few relevant scenarios. However, when assessing the overall MOE for 2-propanol, it must be taken into account that this biocide is also used as a solvent, which might add considerably to overall exposure. For this application area, our market research did not yield any detailed information, so that it could not be considered in a quantitative way.

Furthermore, the use of biocides with sensitizing properties deserves further attention and detailed evaluation. For CMI/MI, glutardialdehyde, formaldehyde and chloroacetamide, the concentration in the household products was in the same order of magnitude as the concentration which induces sensitization. The sensitization prevalence of these biocides was 2-3% in studies with 7800 patients from dermatological hospitals [25] and is also high in the general population (0.4% for CMI/MI [25]). A detailed risk characterization for these biocides is therefore needed.

## Conclusion

This investigation shows that a large number of household products contain biocides. Our analysis further revealed that the number of biocidal active substances on the market is limited and that the active substances used can be attributed to several main structural classes and principles of mode of action. There seems to be a limited number of biocidal active substances available, which have both good efficacy in preventing growth of microorganisms and lack of potential health effects. With respect to the possibility that microbes become resistant, a variety of different biocides, however, is desirable.

Several biocides in household products are well known skin sensitizers. It is desirable to replace these biocides with non-sensitizing biocides. In general, the need for preservation should be evaluated carefully for each consumer product.

Our assessment involves many worst case assumptions. However, it allows a comparative risk assessment and identification of important exposure scenarios and biocides of preliminary concern. A refined exposure and hazard assessment is necessary before final conclusions can be made. In the framework of the EU Biocides Directive detailed dossiers on exposure and health effects as well as risk assessments have been submitted to the rapporteur member states. Therefore, it is to be expected that a refined health risk assessment for individual biocides used in household products will be possible in the near future.

## Abbreviations

CMI/MI: mixture of 5-chloro-2-methyl-2H-isothiazolin-3-one and 2-methyl-2H-isothiazolin-3-one; MOE: Margin of Exposure; NaOCl: Sodium hypochlorite; NOAEL: No observed adverse effect level; QAC: Quaternary ammonium compounds such as alkyldimethyl benzyl ammoniumchlorides; TGD: Technical Guidance Document (cf. [11]); TNsG: Technical Notes for Guidance (cf. [12])

## Competing interests

Some author(s) are involved in the preparation of several dossiers of biocidal active substances and biocidal products which have been submitted within the scope of the Biocidal Products Directive. However, this has not affected the independency of the authors. The authors declare that they have no competing interest.

## Authors' contributions

SH, SG, KS, WH and IM jointly conceived the manuscript. SG carried out the market research and derived the typical concentrations together with SH; WH gave general support. SH carried out the exposure estimations and KS the characterisation of the biocide toxicity. WH, IM, SG, SH and KS participated in the selection of relevant biocides. IM and KS evaluated the health risks of the selected biocides in household products. SH wrote the first draft of the manuscript, which all authors revised and updated. All authors read and approved the final manuscript.

## Supplementary Material

Additional file 1 **Spreadsheet_hypotheticum**. Printout of the spreadsheet used for exposure calculation (includes used assumptions and default values per scenario) Click here for file

Additional file 2 **Table S1**. Product categories, product groups and exposure scenarios. Click here for file

Additional file 3 **Table S2**. Overview of the toxicity of selected active substances. Click here for file
