# Supplementary material for: Consumer exposure to biocides - identification of relevant sources and evaluation of possible health effects
Source: Environ Health. 2010 Feb 3;9:7. doi: 10.1186/1476-069X-9-7 (PMC2841155; doi:10.1186/1476-069X-9-7)
Supplement: Additional file 1 — Spreadsheet_hypotheticum. Printout of the spreadsheet used for exposure calculation (includes used assumptions and default values per scenario) [file 1476-069X-9-7-S1.PDF]

Substance parameters

Substance identification

				Comments
Name of active substance	hypotheticum		S	
CAS No. active substance			U	

PC-Data

Molecular weight	250	g/mol	S	
Vapour pressure	2,0E+00	Pa	S	*at 20°C*
Water solubilty	1,0E+06	mg/l	S	*at 20°C miscible*
LogKow	0,2	-	S	
Henry´s law constant	0,0005	Pa * m^3/mol	O	*VP/WS-method*
Physical state (gaseous/liquid/solid)	liquid/solid		S	
maximal concentration in air (vapour)	205,1493218	mg/m^3	O	*at 20 °C, calculated via ideal gas law*

Toxicity data

NOAEL (oral)	100	mg/kg/day	S	
NOAEL (inhalativ)	95,57142857	mg/kg/day	S	*600 mg/m^3 , 8 hours, rat*
NOAEL (dermal)	100	mg/kg/day	S	*not investigated, deviation from oral*
OEL	600	mg/m^3	S	
TDI	120	mg/kg/day	O	*calculated from MAK*

U = Undefined, D = Default, S = Set, O = Output

Default values

			Reference
**General**			
Quantity used per m²	50	ml	*TRGS 525 (1998) chapter 7*
Thickness of layer of product on skin	0,1	mm	*TGD (2003), HERA (2005), Vermeire et al. (1993)*
Temperature	20	°C	*EUSES 2 (average temperature on the workplace)*
Gas constant	8,314	J/(mol*K)	*TGD (2003)*
**Body weight**			
Body weight adult	60	kg	*TGD (2003)*
Body weight children (female, 9 month, median)	8,3	kg	*AUH (1995)*
**Mean body surface areas for adult (men)**			
Head (Face)	0,118	m^2	*US EPA (1997) cited in TGD (2003)*
Trunk	0,569	m^2	*US EPA (1997) cited in TGD (2003)*
Upper extremities	0,319	m^2	*US EPA (1997) cited in TGD (2003)*
Arms	0,228	m^2	*US EPA (1997) cited in TGD (2003)*
Upper arms	0,143	m^2	*US EPA (1997) cited in TGD (2003)*
Forearms	0,114	m^2	*US EPA (1997) cited in TGD (2003)*
Hands (fronts and backs)	0,084	m^2	*US EPA (1997) cited in TGD (2003)*
Fingertips	0,0168	m^2	*ITEM (20% of hands)*
Lower extremities	0,636	m^2	*US EPA (1997) cited in TGD (2003)*
Legs	0,506	m^2	*US EPA (1997) cited in TGD (2003)*
Thighs	0,198	m^2	*US EPA (1997) cited in TGD (2003)*
Lower legs	0,207	m^2	*US EPA (1997) cited in TGD (2003)*
Feet	0,112	m^2	*US EPA (1997) cited in TGD (2003)*
Whole body	1,94	m^2	*US EPA (1997) cited in TGD (2003)*
**Body surface areas for children (< 1 year)**			
Whole body children	0,3925	m^2	*Biocides Steering Group (1998)*
Head children (18.2% of whole body)	0,071435	m^2	*US EPA (1997)*
Trunk children (35.7% of whole body)	0,1401225	m^2	*US EPA (1997)*
Arms children (13.7% of whole body)	0,0537725	m^2	*US EPA (1997)*
Hands children (5.3% of whole body)	0,0208025	m^2	*US EPA (1997)*
Legs children (20.6% of whole body)	0,080855	m^2	*US EPA (1997)*
Feet children (6.54% of whole body)	0,0256695	m^2	*US EPA (1997)*
**Respiration rate**			
Human (worker)	1,5	m^3/h	*EUSES 2, TGD (2003)*
Human (consumer, environment)	0,83333333	m^3/h	*EUSES 2, TGD (2003)*
Children (< 1 year, medium activity)	0,24166667	m^3/h	*AUH (1995) cited in TGD (2003)*
**Absorption**			
Bioavailability after inhalation	75	%	*Biocides Steering Group (1998)*
Bioavailability after dermal exposure	100	%	*Biocides Steering Group (1998)*
Bioavailability after ingestion	100	%	*Biocides Steering Group (1998)*
**Room volume**			
Living room	58	m^3	*Bremmer and van Veen (2000) cited in TGD (2003)*
Room 1	40	m^3	*Bremmer and van Veen (2000) cited in TGD (2003)*
Room 2	30	m^3	*Bremmer and van Veen (2000) cited in TGD (2003)*
Sleeping room	16	m^3	*Bremmer and van Veen (2000) cited in TGD (2003)*
Kitchen	15	m^3	*Bremmer and van Veen (2000) cited in TGD (2003)*
Toilet	2,5	m^3	*Bremmer and van Veen (2000) cited in TGD (2003)*
Bathroom	10	m^3	*ConsExpo 4, Bremmer (1998) cited in Biocides Steering Group (1998); Bremmer and van Veen (2000) cited in TGD (2003) = 4 m^3*

Chronic exposure to household products: hypotheticum

Scenario	consumer (adult) inhalative	dermal	oral	total	bystander (child, 9-12 month) inhalative	dermal	oral	total
	mg/kg/day				mg/kg/day			
Scenario 1: cleaning/disinfection: small surfaces using concentrate	0,83	4,0	accidental	4,8	1,7	1,3	accidental	3,0
Scenario 2: cleaning/disinfection: small surfaces using dilution of liquid concentrate	0,11	0,45	accidental	0,56	0,24	0,028	accidental	0,27
Scenario 3: cleaning/disinfection: surfaces using dilution of liquid concentrate	0,044	0,064	accidental	0,11	0,09	0,0039	accidental	0,10
Scenario 4: cleaning/disinfection: small surfaces or objects using (ready for-use) spray	0,57	5,0	accidental	5,0	1,2	1,3	accidental	2,5
Scenario 5: cleaning/disinfection: shoes using (ready for-use) spray	0,00080	0,031	accidental	0,031	0,0017	0,10	accidental	0,10
Scenario 6: cleaning/disinfection: textiles (e.g. jacket, sofa) using (ready for-use) spray	0,0057	0,27	accidental	0,27	0,012	0,30	accidental	0,31
Scenario 7: cleaning/disinfection: surfaces using spray (dilution of liquid concentrate)	0,40	0,28	accidental	0,67	0,83	0,036	accidental	0,87
Scenario 8: cleaning/disinfection: surfaces using (ready-for-use) spray (incl. wiping of surface)	0,63	5,0	accidental	5,0	1,3	2,5	accidental	3,8
Scenario 9: cleaning/disinfection: surfaces using (ready-for-use) spray (incl. washing-up of surface)	0,16	5,0	accidental	5,0	0,33	1,3	accidental	1,6
Scenario 10: cleaning/disinfection: disinfection wipe (small surfaces)	0,43	3,3	accidental	3,8	0,90	12	accidental	13
Scenario 11: cleaning/disinfection: toilet cleaners (liquid/gel)	0,0031	0,080	accidental	0,083	0,0065	0	accidental	0,0065
Scenario 12: cleaning/disinfection: drain cleaner (liquid)	0,00012	0,0031	accidental	0,0032	0,00025	0	accidental	0,00025
Scenario 13: cleaning/disinfection: swimming pool water treatment	2,5E-06	0,040	3,7E-06	0,040	5,3E-06	5,3E-05	2,7E-05	8,5E-05
Scenario 14: cleaning/disinfection: textiles, machine wash using laundry compact (liquid/gel)	1,2	0,40	accidental	1,6	8,4	0,0066	accidental	8,4
Scenario 14b: cleaning/disinfection: textiles, machine wash using laundry additives (liquid)	0,48	0,16	accidental	0,63	3,4	0,0027	accidental	3,4
Scenario 15: cleaning/disinfection: textiles, hand wash using laundry compact (liquid/gel)	1,7	0,50	accidental	2,2	12	0,0095	accidental	12
Scenario 15b: cleaning/disinfection: textiles, hand wash using laundry additive (liquid)	0,95	0,20	accidental	1,1	6,9	0,0054	accidental	6,9
Scenario 16: textiles, wearing clothes with antimicrobial protection	1,19	0,00	accidental	1,2	7,7	0,0067	accidental	7,7
Scenario 17: cleaning/disinfection: machine dishwashing (tablet, powder)	0,036	0,0067	8,1E-06	0,042	0,07	0,00	0,00	0,07
Scenario 17b: cleaning/disinfection: machine dishwashing (liquid)	0,00035	0,28	8,1E-06	0,28	0,00074	0	5,8E-05	0,00080
Scenario 17c: cleaning/disinfection: hand dishwashing (liquid concentrate)	4,9E-05	0,85	0,00050	0,85	0,00010	0	0,0036	0,0037
Scenario 18: personal care: whole body rinse-off	0,083	2,0	accidental	2,0	0,17	14	accidental	14
Scenario 19: personal care: whole body leave-on	2,5	2,5	accidental	2,5	18	18	accidental	18
Scenario 20: personal care: head leave-on	1,7	1,7	accidental	1,7	12	2,5	accidental	12
Scenario 21: personal care: hands leave-on	0,57	0,57	accidental	0,57	4,1	0	accidental	4,1

Chronic exposure to household products: hypotheticum

Scenario	consumer (adult) inhalative	dermal	oral	total	bystander (child, 9-12 month) inhalative	dermal	oral	total
	mg/kg/day				mg/kg/day			
Scenario 22: personal care: (ready for-use) spray (deo spray, hair spray)	1,7	1,7	accidental	1,7	12	2,5	accidental	12
Scenario 23: personal care: wet tissues	51	33	accidental	66	65	238	accidental	302
Scenario 24: personal care: hands/feet using (ready for-use) spray	0,0048	0,23	accidental	0,23	0,01	0	accidental	0,29
Scenario 25: personal care: toothpaste	negligible	negligible	0,027	0,027	negligible	negligible	1,3	1,3
Scenario 25b: personal care: mouth wash	negligible	negligible	0,83	0,83	negligible	negligible	6,0	6,0
Scenario 25c: personal care: lip stick	negligible	negligible	0,010	0,010	negligible	negligible	0,072	0,072
Scenario 26: home improvement: painting, latex paint	0,35	0,094	accidental	0,45	0,74	0,22	accidental	0,95
Scenario 26b: home improvement: painting, latex paint + anti mould additive	0,066	0,0039	accidental	0,069	0,14	0,0036	accidental	0,14
Scenario 27: home improvement: varnish (water-based)	0,35	0,039	accidental	0,39	0,74	0,025	accidental	0,76
Scenario 28: home improvement: wallpaper paste anti mould additive	0,20	0,0062	accidental	0,21	0,42	0,0090	accidental	0,43
Scenario 29: home improvement: (ready-for-use) floor adhesive	0,35	0,045	accidental	0,40	0,74	0,031	accidental	0,77
Scenario 30: handicrafts: playing with finger paints	0,22	0,30	accidental	0,52	0,47	3,8	accidental	4,2
Scenario 31: repellent: liquid	0,17	0,17	accidental	0,17	0,42	0,42	accidental	0,42
Scenario 32: repellent, spray	0,17	0,17	accidental	0,17	0,42	0,42	accidental	0,42
Scenario 33: insecticide: spray, air space	0,012	0,29	accidental	0,29	0,026	2,1	accidental	2,1
Scenario 33b: insecticide: spray, targetted spot	0,47	0,61	accidental	1,1	0,99	1,8	accidental	2,8
Scenario 33c: insecticide: spray, crack and crevice	0,31	0,52	accidental	0,83	0,66	1,4	accidental	2,1
Scenario 34: insecticide: liquid, crack and crevice	0,015	0,033	accidental	0,033	0,030	5,0E-05	accidental	0,031
Scenario 35: insecticide: dilution of powder, crack and crevice	0,39	0,012	accidental	0,40	0,82	0,0018	accidental	0,83
Scenario 35b: insecticide: powder, crack and crevice	0,093	0,00038	accidental	0,093	0,19	0,0014	accidental	0,20
Scenario 36: insecticide: evaporator, indoor	0,0027	0,00007	accidental	0,0028	0,0058	0	accidental	0,0058
Scenario 36b: insecticide: evaporator, wardrobe	0,00018	1,8E-07	accidental	0,00018	0,0013	0	accidental	0,0013
Scenario 36c: insecticide: evaporator, dustbin	0,00027	2,7E-06	accidental	0,00027	0,0018	0	accidental	0,0018
Scenario 37: insecticide: strip	0,00027	2,7E-05	accidental	0,00027	0,0020	0	accidental	0,0020
Scenario 38: insecticide: trap	0,0079	0	accidental	0,0079	0,053	0	accidental	0,053
**Sum**	**69**	**70**	**0,87**	**114**	**163**	**304**	**7,4**	**451**

Chronic exposure to household products: hypotheticum

Scenario	consumer (adult) inhalative	dermal	oral	total	bystander (child, 9-12 month) inhalative	dermal	oral	total
	MOE				MOE			
Scenario 1: cleaning/disinfection: small surfaces using concentrate	115	25	accidental	21	55	80	accidental	33
Scenario 2: cleaning/disinfection: small surfaces using dilution of liquid concentrate	834	223	accidental	178	398	3627	accidental	373
Scenario 3: cleaning/disinfection: surfaces using dilution of liquid concentrate	2195	1566	accidental	931	1047	25460	accidental	1051
Scenario 4: cleaning/disinfection: small surfaces or objects using (ready for-use) spray	167	20	accidental	20	80	80	accidental	41
Scenario 5: cleaning/disinfection: shoes using (ready for-use) spray	119601	3259	accidental	3259	57051	983	accidental	968
Scenario 6: cleaning/disinfection: textiles (e.g. jacket, sofa) using (ready for-use) spray	16744	365	accidental	365	7987	339	accidental	325
Scenario 7: cleaning/disinfection: surfaces using spray (dilution of liquid concentrate)	241	362	accidental	149	115	2801	accidental	116
Scenario 8: cleaning/disinfection: surfaces using (ready-for-use) spray (incl. wiping of surface)	153	20	accidental	20	73	40	accidental	26
Scenario 9: cleaning/disinfection: surfaces using (ready-for-use) spray (incl. washing-up of surface)	612	20	accidental	20	292	80	accidental	63
Scenario 10: cleaning/disinfection: disinfection wipe (small surfaces)	224	30	accidental	27	107	8	accidental	8
Scenario 11: cleaning/disinfection: toilet cleaners (liquid/gel)	30816	1253	accidental	1207	14699	#DIV/0!	accidental	15381
Scenario 12: cleaning/disinfection: drain cleaner (liquid)	805588	32589	accidental	31376	384275	#DIV/0!	accidental	402081
Scenario 13: cleaning/disinfection: swimming pool water treatment	37586292	2505	27000000	2504	17929093	1903185	3735000	1181366
Scenario 14: cleaning/disinfection: textiles, machine wash using laundry compact (liquid/gel)	82	248	accidental	64	11	15105	accidental	12
Scenario 14b: cleaning/disinfection: textiles, machine wash using laundry additives (liquid)	201	620	accidental	158	28	37108	accidental	29
Scenario 15: cleaning/disinfection: textiles, hand wash using laundry compact (liquid/gel)	57	200	accidental	46	8	10573	accidental	8
Scenario 15b: cleaning/disinfection: textiles, hand wash using laundry additive (liquid)	101	498	accidental	87	14	18554	accidental	15
Scenario 16: textiles, wearing clothes with antimicrobial protection	81	21709	accidental	84	12	14843	accidental	13
Scenario 17: cleaning/disinfection: machine dishwashing (tablet, powder)	2683	15000	12412121	2365	1280	#DIV/0!	1717010	1338
Scenario 17b: cleaning/disinfection: machine dishwashing (liquid)	269713	357	12412121	357	128656	#DIV/0!	1717010	124831
Scenario 17c: cleaning/disinfection: hand dishwashing (liquid concentrate)	1944764	118	202020	118	927675	#DIV/0!	27946	27164
Scenario 18: personal care: whole body rinse-off	1147	50	accidental	50	547	7	accidental	7
Scenario 19: personal care: whole body leave-on	38	40	accidental	40	5	6	accidental	6
Scenario 20: personal care: head leave-on	57	60	accidental	60	8	40	accidental	8
Scenario 21: personal care: hands leave-on	169	176	accidental	176	23	#DIV/0!	accidental	24

Chronic exposure to household products: hypotheticum

Scenario	consumer (adult) inhalative	dermal	oral	total	bystander (child, 9-12 month) inhalative	dermal	oral	total
	MOE				MOE			
Scenario 22: personal care: (ready for-use) spray (deo spray, hair spray)	57	60	accidental	60	8	40	accidental	8
Scenario 23: personal care: wet tissues	2	3	accidental	2	1	0	accidental	0
Scenario 24: personal care: hands/feet using (ready for-use) spray	19933	435	accidental	435	9508	#DIV/0!	accidental	343
Scenario 25: personal care: toothpaste	negligible	negligible	3750	3750	negligible	negligible	78	78
Scenario 25b: personal care: mouth wash	negligible	negligible	120	120	negligible	negligible	17	17
Scenario 25c: personal care: lip stick	negligible	negligible	10000	10000	negligible	negligible	1383	1383
Scenario 26: home improvement: painting, latex paint	272	1067	accidental	225	130	461	accidental	105
Scenario 26b: home improvement: painting, latex paint + anti mould additive	1458	25886	accidental	1441	696	27665	accidental	709
Scenario 27: home improvement: varnish (water-based)	272	2556	accidental	256	130	4045	accidental	131
Scenario 28: home improvement: wallpaper paste anti mould additive	477	16114	accidental	484	227	11066	accidental	233
Scenario 29: home improvement: (ready-for-use) floor adhesive	272	2225	accidental	252	130	3236	accidental	130
Scenario 30: handicrafts: playing with finger paints	429	334	accidental	192	205	27	accidental	24
Scenario 31: repellent: liquid	554	579	accidental	579	230	240	accidental	240
Scenario 32: repellent, spray	554	579	accidental	579	230	240	accidental	240
Scenario 33: insecticide: spray, air space	7708	348	accidental	348	3677	48	accidental	48
Scenario 33b: insecticide: spray, targetted spot	203	163	accidental	92	97	55	accidental	36
Scenario 33c: insecticide: spray, crack and crevice	305	193	accidental	120	145	70	accidental	48
Scenario 34: insecticide: liquid, crack and crevice	6571	3008	accidental	3008	3135	2000433	accidental	3275
Scenario 35: insecticide: dilution of powder, crack and crevice	243	8279	accidental	247	116	56012	accidental	121
Scenario 35b: insecticide: powder, crack and crevice	1028	263221	accidental	1071	490	72825	accidental	509
Scenario 36: insecticide: evaporator, indoor	34793	1460000	accidental	35520	16597	#DIV/0!	accidental	17366
Scenario 36b: insecticide: evaporator, wardrobe	523254	547500000	accidental	547500	72383	#DIV/0!	accidental	75738
Scenario 36c: insecticide: evaporator, dustbin	348836	36500000	accidental	365000	52346	#DIV/0!	accidental	54772
Scenario 37: insecticide: strip	348836	3650000	accidental	365000	48256	#DIV/0!	accidental	50492
Scenario 38: insecticide: trap	12029	#DIV/0!	accidental	12586	1799	#DIV/0!	accidental	1883
Sum	1	1	115	1	1	0	14	0

Scenario 1: cleaning/disinfection: small surfaces using concentrate

Scenario description

				Comments
Name of product	disinfection cleaner		S	
Physical state product (liquid/solid)	liquid		S	
Density product	1	g/cm^3	D	
Concentration of active substance in product (concentrate)	1	%	S	*Frame formulation*
Concentration of active substance in product (concentrate) (mg/l)	10000	mg/l	O	
User	Consumer		S	
Bystander	Children		S	
Temperature	20	°C	D	*room temperature*

Mixing & Loading

Task A: not applicable

Application

Task B: cleaning: dipping into concentrate, wiping/mopping of surface

Number of tasks per year	365	tasks/yr	S	*once per day, max [AISE (2002) cited in TGD (2003)]*
Duration of task	20	min/task	S	*max [AISE (2002) cited in TGD (2003)]*
Surface (treated per task)	0,8	m^2/task	S	
Volume of product used per m^2	50	ml/m^2	D	
Volume of product used per task	40	ml/task	O	
Quantity of product used per task	40	g/task	O	*max 40 g [AISE (2002) cited in TGD (2003)]*
Quantity of active substance used per task	400	mg/task	O	
Model inhalation exposure	Exposure to vapour / Instantaneous release (limited to vapour pressure)			
Room volume	10	m^3	S	*Bathroom*
Model dermal exposure	Direct dermal contact			
Contact area between product (concentrate) and skin	0,198	m^2	S	*Forearms, Hands*
Model oral exposure	accidental			

Post application phase

Task C: residence time

Number of tasks per year	365	tasks/yr	S	*once per day, max [AISE (2002) cited in TGD (2003)]*
Duration of task	100	min/task	S	*ConsExpo: 120 min total inhalative exposure*
Quantity of active substance used per task	400	mg/task	O	
Model inhalation exposure	Exposure to vapour / Instantaneous release (limited to vapour pressure)			
Room volume	10	m^3	S	*Bathroom*
Model dermal exposure	Direct dermal contact			
Contact area between product (concentrate) and skin	0,042	m^2	S	*50% Hands*
Contact area between product (concentrate) and skin children	0,01040125	m^2	S	*50% Hands Children*
Model oral exposure	accidental			

Disposal

Task D: not applicable

Summary Results Exposure

	User	Consumer	Bystander	Children
Highest potential exposure acute (all amount used is absorbed)	6,666666667	mg/kg bw	48,19277108	mg/kg bw
Highest potential exposure chronic (all amount used is absorbed)	6,666666667	mg/kg bw/day	48,19277108	mg/kg bw/day
Highest potential concentration in air	40	mg/m^3	40	mg/m^3
Inhalation acute	0,833333333	mg/kg bw	1,746987952	mg/kg bw
Inhalation chronic	0,833333333	mg/kg bw/day	1,746987952	mg/kg bw/day
Dermal acute	4	mg/kg bw	1,253162651	mg/kg bw
Dermal chronic	4	mg/kg bw/day	1,253162651	mg/kg bw/day
Oral acute	accidental		accidental	
Oral chronic	accidental		accidental	
Intake acute	4,833333333	mg/kg bw	3,000150602	mg/kg bw
Daily intake chronic	4,833333333	mg/kg bw/day	3,000150602	mg/kg bw/day

U = Undefined, D = Default, S = Set, O = Output, REM = Remark, not used in calculation

Scenario 1: cleaning/disinfection: small surfaces using concentrate

Results / Output

**Mixing & Loading**	Task A: not applicable					

**Application**	Task B: cleaning: dipping into concentrate, wiping/mopping of surface					
	**Primary exposure**	**User**		**Secondary exposure**	**Bystander**	
**Inhalation**						
Concentration of potential exposure	40	mg/m^3	O	40	mg/m^3	O
Inhalation	0,555555556	mg/min	O	0,161111111	mg/min	O
Absorption per task	8,333333333	mg/task	O	2,416666667	mg/task	O
Exposure per task / Acute Dose	0,138888889	mg/kg bw	O	0,291164659	mg/kg bw	O
Exposure per day / Chronic Dose	0,138888889	mg/kg bw/day	O	0,291164659	mg/kg bw/day	O
**Dermal**						
Contact area between product and skin	0,198	m^2	S			
Volume of contact	19,8	ml	O			
Quantity of contact (mg)	198	mg	O			
Dermal load (mg/cm^2)	0,1	mg/cm^2	O			
Absorption (mg)	198	mg	O			
Exposure per task / Acute Dose	3,3	mg/kg bw	O	no		S
Exposure per day / Chronic Dose	3,3	mg/kg bw/day	O	no		S
**Oral**						
Exposure / Dose	accidental		S	accidental		S
**Intake**						
Exposure per task / Acute Dose	3,438888889	mg/kg bw	O	0,291164659	mg/kg bw	O
Exposure per day / Chronic Dose	3,438888889	mg/kg bw/day	O	0,291164659	mg/kg bw/day	O

**Post application phase**	Task C: residence time					
	**Primary exposure**	**User**		**Secondary exposure**	**Bystander**	
**Inhalation**						
Concentration of potential exposure	40	mg/m^3	O	40	mg/m^3	O
Inhalation	0,555555556	mg/min	O	0,161111111	mg/min	O
Absorption per task	41,66666667	mg/task	O	12,08333333	mg/task	O
Exposure per task / Acute Dose	0,694444444	mg/kg bw	O	1,455823293	mg/kg bw	O
Exposure per day / Chronic Dose	0,694444444	mg/kg bw/day	O	1,455823293	mg/kg bw/day	O
**Dermal**						
Contact area between product and skin	0,042	m^2	S	0,01040125	m^2	S
Volume of contact	4,2	ml	O	1,040125	ml	O
Quantity of contact (mg)	42	mg	O	10,40125	mg	O
Dermal load (mg/cm^2)	0,1	mg/cm^2	O	0,1	mg/cm^2	O
Absorption (mg)	42	mg	O	10,40125	mg	O
Exposure per task / Acute Dose	0,7	mg/kg bw	O	1,253162651	mg/kg bw	O
Exposure per day / Chronic Dose	0,7	mg/kg bw/day	O	1,253162651	mg/kg bw/day	O
**Oral**						
Exposure / Dose	accidental		S	accidental		S
**Intake**						
Exposure per task / Acute Dose	1,394444444	mg/kg bw	O	2,708985944	mg/kg bw	O
Exposure per day / Chronic Dose	1,394444444	mg/kg bw/day	O	2,708985944	mg/kg bw/day	O

**Disposal**	Task D: not applicable					

U = Undefined, D = Default, S = Set, O = Output, REM = Remark, not used in calculation

Scenario 2: cleaning/disinfection: small surfaces using dilution of liquid concentrate

Scenario description

				Comments
Name of product	disinfection cleaner		S	
Physical state product (liquid/solid)	liquid		S	
Density product	1	g/cm^3	D	
Concentration of active substance in product (concentrate)	1	%	S	*Frame Formulation*
Concentration of active substance in product (concentrate) (mg/l)	10000	mg/l	O	
User	Consumer		S	
Bystander	Children		S	
Temperature	20	°C	D	*room temperature*
**Mixing & Loading**	Task A: simple dilution with water in bucket			
Number of tasks per year	365	tasks/yr	S	*max once a day [AISE (2002) cited in TGD (2003)]*
Duration of task	1,333333333	min/task	S	*80 sec. [TNsG 2002 p. 252 mixing for spray]*
Volume of product used per task	44	ml/task	S	
Quantity of water used per task	2	L/task	S	
Quantity of product used per task	44	g/task	O	*max 110 g /5 L wash water volume [AISE (2002) cited in TGD (2*
Quantity of active substance used per task	440	mg/task	O	
Concentration of active substance in ready-for-use solution	220	mg/l	O	
Model inhalation exposure	Exposure to vapour / Fugacity concept (equilibrium between ready-for-use solution and air, limited to vapour pressure)			
Room volume	1	m^3	S	*cloud around user*
REM Evaporation from mixture, release area concentrate	*0,002*	*m^2*	*S*	*bottle diameter 5 cm [TNsG 2002 p. 252]*
REM Evaporation from mixture, release area ready-for-use solution	*0,070685835*	*m^2*	*S*	*bucket diameter 30 cm*
Model dermal exposure	Direct dermal contact			
Contact area between product (concentrate) and skin	0,0168	m^2	S	*Fingertips*
Model oral exposure	accidental			
**Application**	Task B: cleaning: dipping into ready-for-use solution, wiping/mopping of surface			
Number of tasks per year	365	tasks/yr	S	*max once a day [AISE (2002) cited in TGD (2003)]*
Duration of task	20	min/task	S	*max [AISE (2002) cited in TGD (2003)]*
Surface (treated per task)	5	m^2/task	S	
Quantity of ready-for-use solution used per m^2	50	ml/m^2	D	
Quantity of ready-for-use solution used per task	250	ml/task	O	
Concentration of active substance in ready-for-use solution	220	mg/l	O	
Quantity of active substance used per task	55	mg/task	O	
Model inhalation exposure	Exposure to vapour / Instantaneous release (limited to vapour pressure)			
Room volume	10	m^3	S	*Bathroom*
Model dermal exposure	Direct dermal contact			
Contact area between ready-for-use solution and skin	0,2187	m^2	S	*Forearms, Hands, 10% Lower legs*
Model oral exposure	accidental			
**Post application phase**	Task C: residence time			
Number of tasks per year	365	tasks/yr	S	*max once a day [AISE (2002) cited in TGD (2003)]*
Duration of task	100	min/task	S	*ConsExpo: 120 min total inhalative exposure*
Concentration of active substance in ready-for-use solution	220	mg/l	O	
Quantity of active substance used per task	55	mg/task	O	
Model inhalation exposure	Exposure to vapour / Instantaneous release (limited to vapour pressure)			
Room volume	10	m^3	S	*Bathroom*
Model dermal exposure	Direct dermal contact			
Contact area between ready-for-use solution and skin	0,042	m^2	S	*50% Hands*
Contact area between ready-for-use solution and skin children	0,01040125	m^2	S	*50% Hands Children*
Model oral exposure	accidental			
**Disposal**	Task D: excessive ready-for-use solution is disposed to the main drainage			
Number of tasks per year	365	tasks/yr	S	*max once a day [AISE (2002) cited in TGD (2003)]*
Duration of task	1	min/task	S	
Volume of ready-for-use solution disposed per task	1,75	L/task	S	
Concentration of active substance in ready-for-use solution	220	mg/l	O	
Quantity of active substance disposed per task	385	mg/task	O	
Model inhalation exposure	Exposure to vapour / Fugacity concept (equilibrium between ready-for-use solution and air, limited to vapour pressure)			
Room volume	1	m^3	S	
REM Evaporation from mixture, release area ready-for-use solution	*0,070685835*	*m^2*	*S*	*bucket diameter 30 cm*
Model dermal exposure	Direct dermal contact			
Contact area between ready-for-use solution and skin	0,198	m^2	S	*Hands, Forearms*
Model oral exposure	accidental			

Summary Results Exposure

	User	Consumer		Bystander	Children	
Highest potential exposure acute (all amount used is absorbed)		7,333333333	mg/kg bw		53,01204819	mg/kg bw
Highest potential exposure chronic (all amount used is absorbed)		7,333333333	mg/kg bw/day		53,01204819	mg/kg bw/day
Highest potential concentration in air		5,5	mg/m^3		5,5	mg/m^3
Inhalation acute		0,114601614	mg/kg bw		0,240249167	mg/kg bw
Inhalation chronic		0,114601614	mg/kg bw/day		0,240249167	mg/kg bw/day
Dermal acute		0,44819	mg/kg bw		0,027569578	mg/kg bw
Dermal chronic		0,44819	mg/kg bw/day		0,027569578	mg/kg bw/day
Oral acute	accidental			accidental		
Oral chronic	accidental			accidental		
Intake acute		0,562791614	mg/kg bw		0,267818746	mg/kg bw
Daily intake chronic		0,562791614	mg/kg bw/day		0,267818746	mg/kg bw/day

U = Undefined, D = Default, S = Set, O = Output, REM = Remark, not used in calculation

Scenario 2: cleaning/disinfection: small surfaces using dilution of liquid concentrate

Results / Output

Mixing & Loading

Task A: simple dilution with water in bucket

	Primary exposure	User		Secondary exposure	Bystander	
**Inhalation**						
Concentration of potential exposure	0,045128222	mg/m^3	O	0,045128222	mg/m^3	O
Inhalation	0,000626781	mg/min	O	0,000181766	mg/min	O
Absorption per task	0,000626781	mg/task	O	0,000181766	mg/task	O
Exposure per task / Acute Dose	1,04463E-05	mg/kg bw	O	2,18996E-05	mg/kg bw	O
Exposure per day / Chronic Dose	1,04463E-05	mg/kg bw/day	O	2,18996E-05	mg/kg bw/day	O
**Dermal**						
Contact area between product and skin	0,0168	m^2	S			
Volume of contact	1,68	ml	O			
Quantity of contact (mg)	16,8	mg	O			
Dermal load (mg/cm^2)	0,1	mg/cm^2	O			
Absorption (mg)	16,8	mg	O			
Exposure per task / Acute Dose	0,28	mg/kg bw	O	no		S
Exposure per day / Chronic Dose	0,28	mg/kg bw/day	O	no		S
**Oral**						
Exposure / Dose	accidental		S	accidental		S
**Intake**						
Exposure per task / Acute Dose	0,280010446	mg/kg bw	O	2,18996E-05	mg/kg bw	O
Exposure per day / Chronic Dose	0,280010446	mg/kg bw/day	O	2,18996E-05	mg/kg bw/day	O

Application

Task B: cleaning: dipping into ready-for-use solution, wiping/mopping of surface

	Primary exposure	User		Secondary exposure	Bystander	
**Inhalation**						
Concentration of potential exposure	5,5	mg/m^3	O	5,5	mg/m^3	O
Inhalation	0,076388889	mg/min	O	0,022152778	mg/min	O
Absorption per task	1,145833333	mg/task	O	0,332291667	mg/task	O
Exposure per task / Acute Dose	0,019097222	mg/kg bw	O	0,040035141	mg/kg bw	O
Exposure per day / Chronic Dose	0,019097222	mg/kg bw/day	O	0,040035141	mg/kg bw/day	O
**Dermal**						
Contact area between ready-for-use solution and skin	0,2187	m^2	S			
Volume of contact	21,87	ml	O			
Quantity of contact (mg)	4,8114	mg	O			
Dermal load (mg/cm^2)	0,0022	mg/cm^2	O			
Absorption (mg)	4,8114	mg	O			
Exposure per task / Acute Dose	0,08019	mg/kg bw	O	no		S
Exposure per day / Chronic Dose	0,08019	mg/kg bw/day	O	no		S
**Oral**						
Exposure / Dose	accidental		S	accidental		S
**Intake**						
Exposure per task / Acute Dose	0,099287222	mg/kg bw	O	0,040035141	mg/kg bw	O
Exposure per day / Chronic Dose	0,099287222	mg/kg bw/day	O	0,040035141	mg/kg bw/day	O

Post application phase

Task C: residence time

	Primary exposure	User		Secondary exposure	Bystander	
**Inhalation**						
Concentration of potential exposure	5,5	mg/m^3	O	5,5	mg/m^3	O
Inhalation	0,076388889	mg/min	O	0,022152778	mg/min	O
Absorption per task	5,729166667	mg/task	O	1,661458333	mg/task	O
Exposure per task / Acute Dose	0,095486111	mg/kg bw	O	0,200175703	mg/kg bw	O
Exposure per day / Chronic Dose	0,095486111	mg/kg bw/day	O	0,200175703	mg/kg bw/day	O
**Dermal**						
Contact area between ready-for-use solution and skin	0,042	m^2	S	0,01040125	m^2	S
Volume of contact	4,2	ml	O	1,040125	ml	O
Quantity of contact (mg)	0,924	mg	O	0,2288275	mg	O
Dermal load (mg/cm^2)	0,0022	mg/cm^2	O	0,0022	mg/cm^2	O
Absorption (mg)	0,924	mg	O	0,2288275	mg	O
Exposure per task / Acute Dose	0,0154	mg/kg bw	O	0,027569578	mg/kg bw	O
Exposure per day / Chronic Dose	0,0154	mg/kg bw/day	O	0,027569578	mg/kg bw/day	O
**Oral**						
Exposure / Dose	accidental		S	accidental		S
**Intake**						
Exposure per task / Acute Dose	0,110886111	mg/kg bw	O	0,227745281	mg/kg bw	O
Exposure per day / Chronic Dose	0,110886111	mg/kg bw/day	O	0,227745281	mg/kg bw/day	O

Disposal

Task D: excessive ready-for-use solution is disposed to the main drainage

	Primary exposure	User		Secondary exposure	Bystander	
**Inhalation**						
Concentration of potential exposure	0,045127561	mg/m^3	O	0,045127561	mg/m^3	O
Inhalation	0,000626772	mg/min	O	0,000181764	mg/min	O
Absorption per task	0,000470079	mg/task	O	0,000136323	mg/task	O
Exposure per task / Acute Dose	7,83465E-06	mg/kg bw	O	1,64244E-05	mg/kg bw	O
Exposure per day / Chronic Dose	7,83465E-06	mg/kg bw/day	O	1,64244E-05	mg/kg bw/day	O
**Dermal**						
Contact area between ready-for-use solution and skin	0,198	m^2	S			
Volume of contact	19,8	ml	O			
Quantity of contact (mg)	4,356	mg	O			
Dermal load (mg/cm^2)	0,0022	mg/cm^2	O			
Absorption (mg)	4,356	mg	O			
Exposure per task / Acute Dose	0,0726	mg/kg bw	O	no		S
Exposure per day / Chronic Dose	0,0726	mg/kg bw/day	O	no		S
**Oral**						
Exposure / Dose	accidental		S	accidental		S
**Intake**						
Exposure per task / Acute Dose	0,072607835	mg/kg bw	O	1,64244E-05	mg/kg bw	O
Exposure per day / Chronic Dose	0,072607835	mg/kg bw/day	O	1,64244E-05	mg/kg bw/day	O

U = Undefined, D = Default, S = Set, O = Output, REM = Remark, not used in calculation

Scenario 3: cleaning/disinfection: surfaces using dilution of liquid concentrate

Scenario description

Parameter	Value	Unit		*Comments*
Name of product	disinfection cleaner, floor cleaner		S	
Physical state product (liquid/solid)	liquid		S	
Density product	1	g/cm^3	D	
Concentration of active substance in product (concentrate)	1	%	S	*Frame Formulation*
Concentration of active substance in product (concentrate) (mg/l)	10000	mg/l	O	
User	Consumer		S	
Bystander	Children		S	
Temperature	20	°C	D	*room temperature*

Mixing & Loading

	Task A: simple dilution with water in bucket			
Number of tasks per year	52	tasks/yr	S	
Duration of task	1,333333333	min/task	S	*80 sec. [TNsG 2002 p. 252 spray]*
Volume of product used per task	220	ml/task	S	
Quantity of water used per task	10	L/task	S	
Quantity of product used per task	220	g/task	O	*max 110 g /5 L wash water volume [AISE (2002) cited in TGD (2*
Quantity of active substance used per task	2200	mg/task	S	
Concentration of active substance in ready-for-use solution	220	mg/l	O	
Model inhalation exposure	Exposure to vapour / Fugacity concept (equilibrium between ready-for-use solution and air, limited to vapour pressure)			
Room volume	1	m^3	S	
REM Evaporation from mixture, release area concentrate	*0,002*	*m^2*	*S*	*bottle diameter 5 cm [TNsG 2002 p. 252]*
REM Evaporation from mixture, release area ready-for-use solution	*0,070685835*	*m^2*	*S*	*bucket diameter 30 cm*
Model dermal exposure	Direct dermal contact			
Contact area between Product (concentrate) and skin	0,0168	m^2	S	*Fingertips*
Model oral exposure	accidental			

Application

	Task B: cleaning: dipping into ready-for-use solution, wiping/mopping of surface			
Number of tasks per year	52	tasks/yr	S	
Duration of task	20	min/task	S	
Surface (treated per task)	20	m^2/task	S	
Quantity of ready-for-use solution used per m^2	50	ml/m^2	D	
Quantity of ready-for-use solution used per task	1000	ml/task	O	
Concentration of active substance in ready-for-use solution	220	mg/l	O	
Quantity of active substance used per task	220	mg/task	O	
Model inhalation exposure	Exposure to vapour / Instantaneous release (limited to vapour pressure)			
Room volume	15	m^3	S	*Kitchen*
Model dermal exposure	Direct dermal contact			
Contact area between ready-for-use solution and skin	0,2187	m^2	S	*Forearms, Hands, 10% Lower legs*
Model oral exposure	accidental			

Post application phase

	Task C: residence time			
Number of tasks per year	52	tasks/yr	S	
Duration of task	100	min/task	S	
Concentration of active substance in ready-for-use solution	220	mg/l	O	
Quantity of active substance used per task	220	mg/task	O	
Model inhalation exposure	Exposure to vapour / Instantaneous release (limited to vapour pressure)			
Room volume	15	m^3	S	*Kitchen*
Model dermal exposure	Direct dermal contact			
Contact area between ready-for-use solution and skin	0,042	m^2	S	*50% Hands*
Contact area between ready-for-use solution and skin children	0,01040125	m^2	S	*50% Hands Children*
Model oral exposure	accidental			

Disposal

	Task D: excessive ready-for-use solution is disposed to the main drainage			
Number of tasks per year	52	tasks/yr	S	
Duration of task	1	min/task	S	
Volume of ready-for-use solution disposed per task	9	L/task	S	
Concentration of active substance in ready-for-use solution	220	mg/l	O	
Quantity of active substance disposed per task	1980	mg/task	O	
Model inhalation exposure	Exposure to vapour / Fugacity concept (equilibrium between ready-for-use solution and air, limited to vapour pressure)			
Room volume	1	m^3	S	
REM Evaporation from mixture, release area ready-for-use solution	*0,070685835*	*m^2*	*S*	*bucket diameter 30 cm*
Model dermal exposure	Direct dermal contact			
Contact area between ready-for-use solution and skin	0,198	m^2	S	*Hands, Forearms*
Model oral exposure	accidental			

Summary Results Exposure

	User	Consumer		Bystander	Children	
Highest potential exposure acute (all amount used is absorbed)		36,66666667	mg/kg bw		265,060241	mg/kg bw
Highest potential exposure chronic (all amount used is absorbed)		5,223744292	mg/kg bw/day		37,76200693	mg/kg bw/day
Highest potential concentration in air		14,66666667	mg/m^3		14,66666667	mg/m^3
Inhalation acute		0,305573838	mg/kg bw		0,640600576	mg/kg bw
Inhalation chronic		0,043533807	mg/kg bw/day		0,091263644	mg/kg bw/day
Dermal acute		0,44819	mg/kg bw		0,027569578	mg/kg bw
Dermal chronic		0,063851726	mg/kg bw/day		0,003927721	mg/kg bw/day
Oral acute	accidental			accidental		
Oral chronic	accidental			accidental		
Intake acute		0,753763838	mg/kg bw		0,668170155	mg/kg bw
Daily intake chronic		0,107385533	mg/kg bw/day		0,095191365	mg/kg bw/day

U = Undefined, D = Default, S = Set, O = Output, REM = Remark, not used in calculation

Scenario 3: cleaning/disinfection: surfaces using dilution of liquid concentrate

Results / Output

**Mixing & Loading**	Task A: simple dilution with water in bucket					
	**Primary exposure**	**User**		**Secondary exposure**	**Bystander**	
**Inhalation**						
Concentration of potential exposure	0,045131925	mg/m^3	O	0,045131925	mg/m^3	O
Inhalation	0,000626832	mg/min	O	0,000181781	mg/min	O
Absorption per task	0,000626832	mg/task	O	0,000181781	mg/task	O
Exposure per task / Acute Dose	1,04472E-05	mg/kg bw	O	2,19014E-05	mg/kg bw	O
Exposure per day / Chronic Dose	1,48837E-06	mg/kg bw/day	O	3,1202E-06	mg/kg bw/day	O
**Dermal**						
Contact area between product and skin	0,0168	m^2	S			
Volume of contact	1,68	ml	O			
Quantity of contact (mg)	16,8	mg	O			
Dermal load (mg/cm^2)	0,1	mg/cm^2	O			
Absorption (mg)	16,8	mg	O			
Exposure per task / Acute Dose	0,28	mg/kg bw	O	no		S
Exposure per day / Chronic Dose	0,039890411	mg/kg bw/day	O	no		S
**Oral**						
Exposure / Dose	accidental		S	accidental		S
**Intake**						
Exposure per task / Acute Dose	0,280010447	mg/kg bw	O	2,19014E-05	mg/kg bw	O
Exposure per day / Chronic Dose	0,039891899	mg/kg bw/day	O	3,1202E-06	mg/kg bw/day	O

**Application**	Task B: cleaning: dipping into ready-for-use solution, wiping/mopping of surface					
	**Primary exposure**	**User**		**Secondary exposure**	**Bystander**	
**Inhalation**						
Concentration of potential exposure	14,66666667	mg/m^3	O	14,66666667	mg/m^3	O
Inhalation	0,203703704	mg/min	O	0,059074074	mg/min	O
Absorption per task	3,055555556	mg/task	O	0,886111111	mg/task	O
Exposure per task / Acute Dose	0,050925926	mg/kg bw	O	0,106760375	mg/kg bw	O
Exposure per day / Chronic Dose	0,0072552	mg/kg bw/day	O	0,015209697	mg/kg bw/day	O
**Dermal**						
Contact area between ready-for-use solution and skin	0,2187	m^2	S			
Volume of contact	21,87	ml	O			
Quantity of contact (mg)	4,8114	mg	O			
Dermal load (mg/cm^2)	0,0022	mg/cm^2	O			
Absorption (mg)	4,8114	mg	O			
Exposure per task / Acute Dose	0,08019	mg/kg bw	O	no		S
Exposure per day / Chronic Dose	0,011424329	mg/kg bw/day	O	no		S
**Oral**						
Exposure / Dose	accidental		S	accidental		S
**Intake**						
Exposure per task / Acute Dose	0,131115926	mg/kg bw	O	0,106760375	mg/kg bw	O
Exposure per day / Chronic Dose	0,018679529	mg/kg bw/day	O	0,015209697	mg/kg bw/day	O

**Post application phase**	Task C: residence time					
	**Primary exposure**	**User**		**Secondary exposure**	**Bystander**	
**Inhalation**						
Concentration of potential exposure	14,66666667	mg/m^3	O	14,66666667	mg/m^3	O
Inhalation	0,203703704	mg/min	O	0,059074074	mg/min	O
Absorption per task	15,27777778	mg/task	O	4,430555556	mg/task	O
Exposure per task / Acute Dose	0,25462963	mg/kg bw	O	0,533801874	mg/kg bw	O
Exposure per day / Chronic Dose	0,036276002	mg/kg bw/day	O	0,076048486	mg/kg bw/day	O
**Dermal**						
Contact area between ready-for-use solution and skin	0,042	m^2	S	0,01040125	m^2	S
Volume of contact	4,2	ml	O	1,040125	ml	O
Quantity of contact (mg)	0,924	mg	O	0,2288275	mg	O
Dermal load (mg/cm^2)	0,0022	mg/cm^2	O	0,0022	mg/cm^2	O
Absorption (mg)	0,924	mg	O	0,2288275	mg	O
Exposure per task / Acute Dose	0,0154	mg/kg bw	O	0,027569578	mg/kg bw	O
Exposure per day / Chronic Dose	0,002193973	mg/kg bw/day	O	0,003927721	mg/kg bw/day	O
**Oral**						
Exposure / Dose	accidental		S	accidental		S
**Intake**						
Exposure per task / Acute Dose	0,27002963	mg/kg bw	O	0,561371452	mg/kg bw	O
Exposure per day / Chronic Dose	0,038469975	mg/kg bw/day	O	0,079976207	mg/kg bw/day	O

**Disposal**	Task D: excessive ready-for-use solution is disposed to the main drainage					
	**Primary exposure**	**User**		**Secondary exposure**	**Bystander**	
**Inhalation**						
Concentration of potential exposure	0,045131822	mg/m^3	O	0,045131822	mg/m^3	O
Inhalation	0,000626831	mg/min	O	0,000181781	mg/min	O
Absorption per task	0,000470123	mg/task	O	0,000136336	mg/task	O
Exposure per task / Acute Dose	7,83539E-06	mg/kg bw	O	1,6426E-05	mg/kg bw	O
Exposure per day / Chronic Dose	1,11627E-06	mg/kg bw/day	O	2,34014E-06	mg/kg bw/day	O
**Dermal**						
Contact area between ready-for-use solution and skin	0,198	m^2	S			
Volume of contact	19,8	ml	O			
Quantity of contact (mg)	4,356	mg	O			
Dermal load (mg/cm^2)	0,0022	mg/cm^2	O			
Absorption (mg)	4,356	mg	O			
Exposure per task / Acute Dose	0,0726	mg/kg bw	O	no		S
Exposure per day / Chronic Dose	0,010343014	mg/kg bw/day	O	no		S
**Oral**						
Exposure / Dose	accidental		S	accidental		S
**Intake**						
Exposure per task / Acute Dose	0,072607835	mg/kg bw	O	1,6426E-05	mg/kg bw	O
Exposure per day / Chronic Dose	0,01034413	mg/kg bw/day	O	2,34014E-06	mg/kg bw/day	O

U = Undefined, D = Default, S = Set, O = Output, REM = Remark, not used in calculation

Scenario 4: cleaning/disinfection: small surfaces or objects using (ready for-use) spray

Scenario description

				Comments
Name of product	disinfection spray		S	
Physical state product (liquid/solid)	liquid		S	
Density product	1	g/cm^3	D	
Concentration of active substance in product	1	%	S	*Frame Formulation*
Concentration of active substance in product (mg/l)	10000	mg/l	O	
User	Consumer		S	
Bystander	Children		S	
Temperature	20	°C	D	*room temperature*
**Mixing & Loading**	Task A: not applicable			
**Application**	Task B: spraying onto surface + deposit time			
Number of tasks per year	365	tasks/yr	S	*once per day, max [AISE (2002) cited in TGD (2003)]*
Duration of task	10	min/task	S	*max [AISE (2002) cited in TGD (2003)], ConsExpo: spray duration 0.41 min, exposure duration: 10 min*
Surface (treated per task)	0,6	m^2/task	S	
Quantity of product used per m^2	50	ml/m^2	D	
Quantity of product used per task	30	ml/task	O	*max 30 g [AISE (2002) cited in TGD (2003)], ConsExpo: 0.41 min x 0.78 g/s = 19 g*
Quantity of active substance used per task	300	mg/task	O	
Model inhalation exposure	Exposure to aerosols / Instantaneous release			
Room volume	10	m^3	S	*Bathroom*
Model dermal exposure	Direct dermal contact			
Contact area between product and skin	0,258033333	m^2	S	*Forearms, Hands, 33% Head, 10% Lower legs*
Model oral exposure	accidental			
**Post application phase**	Task C: residence time			
Number of tasks per year	365	tasks/yr	S	*once per day, max [AISE (2002) cited in TGD (2003)]*
Duration of task	100	min/task	S	
Quantity of active substance used per task	300	mg/task	O	
Model inhalation exposure	Exposure to vapour / Instantaneous release (limited to vapour pressure)			
Room volume	10	m^3	S	*Bathroom*
Model dermal exposure	Direct dermal contact			
Contact area between product and skin	0,042	m^2	S	*50% Hands*
Contact area between product and skin children	0,01040125	m^2	S	*50% Hands Children*
Model oral exposure	accidental			
**Disposal**	Task D: not applicable			

Summary Results Exposure

	User	Consumer	Bystander	Children
Highest potential exposure acute (all amount used is absorbed)	5	mg/kg bw	36,14457831	mg/kg bw
Highest potential exposure chronic (all amount used is absorbed)	5	mg/kg bw/day	36,14457831	mg/kg bw/day
Highest potential concentration in air	30	mg/m^3	30	mg/m^3
Inhalation acute	0,572916667	mg/kg bw	1,201054217	mg/kg bw
Inhalation chronic	0,572916667	mg/kg bw/day	1,201054217	mg/kg bw/day
Dermal acute	5	mg/kg bw	1,253162651	mg/kg bw
Dermal chronic	5	mg/kg bw/day	1,253162651	mg/kg bw/day
Oral acute	accidental		accidental	
Oral chronic	accidental		accidental	
Intake acute	5	mg/kg bw	2,454216867	mg/kg bw
Daily intake chronic	5	mg/kg bw/day	2,454216867	mg/kg bw/day

U = Undefined, D = Default, S = Set, O = Output, REM = Remark, not used in calculation

Scenario 4: cleaning/disinfection: small surfaces or objects using (ready for-use) spray

Results / Output

**Mixing & Loading**	Task A: not applicable					

**Application**	Task B: spraying onto surface + deposit time					
	**Primary exposure**	**User**		**Secondary exposure**	**Bystander**	
**Inhalation**						
Concentration of potential exposure	30	mg/m^3	O	30	mg/m^3	O
Inhalation	0,416666667	mg/min	O	0,120833333	mg/min	O
Absorption per task	3,125	mg/task	O	0,90625	mg/task	O
Exposure per task / Acute Dose	0,052083333	mg/kg bw	O	0,109186747	mg/kg bw	O
Exposure per day / Chronic Dose	0,052083333	mg/kg bw/day	O	0,109186747	mg/kg bw/day	O
**Dermal**						
Contact area between product and skin	0,258033333	m^2	S			
Volume of contact	25,80333333	ml	O			
Quantity of contact (mg)	258,0333333	mg	O			
Dermal load (mg/cm^2)	0,1	mg/cm^2	O			
Absorption (mg)	258,0333333	mg	O			
Exposure per task / Acute Dose	4,300555556	mg/kg bw	O	no		S
Exposure per day / Chronic Dose	4,300555556	mg/kg bw/day	O	no		S
**Oral**						
Exposure / Dose	accidental		S	accidental		S
**Intake**						
Exposure per task / Acute Dose	4,352638889	mg/kg bw	O	0,109186747	mg/kg bw	O
Exposure per day / Chronic Dose	4,352638889	mg/kg bw/day	O	0,109186747	mg/kg bw/day	O

**Post application phase**	Task C: residence time					
	**Primary exposure**	**User**		**Secondary exposure**	**Bystander**	
**Inhalation**						
Concentration of potential exposure	30	mg/m^3	O	30	mg/m^3	O
Inhalation	0,416666667	mg/min	O	0,120833333	mg/min	O
Absorption per task	31,25	mg/task	O	9,0625	mg/task	O
Exposure per task / Acute Dose	0,520833333	mg/kg bw	O	1,09186747	mg/kg bw	O
Exposure per day / Chronic Dose	0,520833333	mg/kg bw/day	O	1,09186747	mg/kg bw/day	O
**Dermal**						
Contact area between product and skin	0,042	m^2	S	0,01040125	m^2	S
Volume of contact	4,2	ml	O	1,040125	ml	O
Quantity of contact (mg)	42	mg	O	10,40125	mg	O
Dermal load (mg/cm^2)	0,1	mg/cm^2	O	0,1	mg/cm^2	O
Absorption (mg)	42	mg	O	10,40125	mg	O
Exposure per task / Acute Dose	0,7	mg/kg bw	O	1,253162651	mg/kg bw	O
Exposure per day / Chronic Dose	0,7	mg/kg bw/day	O	1,253162651	mg/kg bw/day	O
**Oral**						
Exposure / Dose	accidental		S	accidental		S
**Intake**						
Exposure per task / Acute Dose	1,220833333	mg/kg bw	O	2,34503012	mg/kg bw	O
Exposure per day / Chronic Dose	1,220833333	mg/kg bw/day	O	2,34503012	mg/kg bw/day	O

**Disposal**	Task D: not applicable					

Scenario 5: cleaning/disinfection: shoes using (ready for-use) spray

Scenario description

				Comments
Name of product	disinfection spray, shoe polish spray		S	
Physical state product (liquid/solid)	liquid		S	
Density product	1	g/cm^3	D	*ca.*
Concentration of active substance in product	1	%	S	*Frame Formulation*
Concentration of active substance in product (mg/l)	10000	mg/l	O	
User	Consumer		S	
Bystander	Children		S	
Temperature	20	°C	D	*room temperature*

Mixing & Loading

Task A: not applicable

Application

Task B: spraying onto surface + deposit time

Number of tasks per year	12	tasks/yr	S	*once a month, ConsExpo: shoe polish spray 8 tasks/yr*
Duration of task	5	min/task	S	*ConsExpo: shoe polish spray, spray duration 1.4 min*
Surface (treated per task)	0,112	m^2/task	S	*Shoes = Feet*
Quantity of product used per m^2	50	ml/m^2	D	
Quantity of product used per task	5,6	ml/task	O	*ConsExpo: 0.43 g/s -> 36.12 g*
Quantity of active substance used per task	56	mg/task	O	
Model inhalation exposure	Exposure to aerosols / Instantaneous release			
Room volume	10	m^3	S	*Bathroom*
Model dermal exposure	Direct dermal contact			
Contact area between product and skin	0,198	m^2	S	*Forearms, Hands*
Model oral exposure	accidental			

Post application phase

Task C: residence time

Number of tasks per year	12	tasks/yr	S	*once a month, ConsExpo: shoe polish spray 8 tasks/yr*
Duration of task	20	min/task	S	*ConsExpo: shoe polish spray, exposure duration 25 min*
Quantity of active substance used per task	56	mg/task	O	
Model inhalation exposure	Exposure to vapour / Instantaneous release (limited to vapour pressure)			
Room volume	10	m^3	S	*Bathroom*
Model dermal exposure	Direct dermal contact			
Contact area between product and skin	0,112	m^2	S	*Feet*
Contact area between product and skin children	0,0256695	m^2	S	*Feet Children*
Model oral exposure	accidental			

Disposal

Task D: not applicable

Summary Results Exposure

	User		Consumer	Bystander		Children
Highest potential exposure acute (all amount used is absorbed)		0,933333333	mg/kg bw		6,746987952	mg/kg bw
Highest potential exposure chronic (all amount used is absorbed)		0,030684932	mg/kg bw/day		0,221818782	mg/kg bw/day
Highest potential concentration in air		5,6	mg/m^3		5,6	mg/m^3
Inhalation acute		0,024305556	mg/kg bw		0,050953815	mg/kg bw
Inhalation chronic		0,000799087	mg/kg bw/day		0,001675194	mg/kg bw/day
Dermal acute		0,933333333	mg/kg bw		3,092710843	mg/kg bw
Dermal chronic		0,030684932	mg/kg bw/day		0,101678165	mg/kg bw/day
Oral acute	accidental			accidental		
Oral chronic	accidental			accidental		
Intake acute		0,933333333	mg/kg bw		3,143664659	mg/kg bw
Daily intake chronic		0,030684932	mg/kg bw/day		0,103353359	mg/kg bw/day

U = Undefined, D = Default, S = Set, O = Output, REM = Remark, not used in calculation

Scenario 5: cleaning/disinfection: shoes using (ready for-use) spray

Results / Output

**Mixing & Loading**	Task A: not applicable					

**Application**	Task B: spraying onto surface + deposit time					
	**Primary exposure**	**User**		**Secondary exposure**	**Bystander**	
**Inhalation**						
Concentration of potential exposure	5,6	mg/m^3	O	5,6	mg/m^3	O
Inhalation	0,077777778	mg/min	O	0,022555556	mg/min	O
Absorption per task	0,291666667	mg/task	O	0,084583333	mg/task	O
Exposure per task / Acute Dose	0,004861111	mg/kg bw	O	0,010190763	mg/kg bw	O
Exposure per day / Chronic Dose	0,000159817	mg/kg bw/day	O	0,000335039	mg/kg bw/day	O
**Dermal**						
Contact area between product and skin	0,198	m^2	S			
Volume of contact	19,8	ml	O			
Quantity of contact (mg)	56	mg	S			
Dermal load (mg/cm^2)	0,028282828	mg/cm^2	O			
Absorption (mg)	56	mg	O			
Exposure per task / Acute Dose	0,933333333	mg/kg bw	O	no		S
Exposure per day / Chronic Dose	0,030684932	mg/kg bw/day	O	no		S
**Oral**						
Exposure / Dose	accidental		S	accidental		S
**Intake**						
Exposure per task / Acute Dose	0,933333333	mg/kg bw	S	0,010190763	mg/kg bw	O
Exposure per day / Chronic Dose	0,030684932	mg/kg bw/day	O	0,000335039	mg/kg bw/day	O

**Post application phase**	Task C: residence time					
	**Primary exposure**	**User**		**Secondary exposure**	**Bystander**	
**Inhalation**						
Concentration of potential exposure	5,6	mg/m^3	O	5,6	mg/m^3	O
Inhalation	0,077777778	mg/min	O	0,022555556	mg/min	O
Absorption per task	1,166666667	mg/task	O	0,338333333	mg/task	O
Exposure per task / Acute Dose	0,019444444	mg/kg bw	O	0,040763052	mg/kg bw	O
Exposure per day / Chronic Dose	0,000639269	mg/kg bw/day	O	0,001340155	mg/kg bw/day	O
**Dermal**						
Contact area between product and skin	0,112	m^2	S	0,0256695	m^2	S
Volume of contact	11,2	ml	O	2,56695	ml	O
Quantity of contact (mg)	56	mg	S	25,6695	mg	O
Dermal load (mg/cm^2)	0,05	mg/cm^2	O	0,1	mg/cm^2	O
Absorption (mg)	56	mg	O	25,6695	mg	O
Exposure per task / Acute Dose	0,933333333	mg/kg bw	O	3,092710843	mg/kg bw	O
Exposure per day / Chronic Dose	0,030684932	mg/kg bw/day	O	0,101678165	mg/kg bw/day	O
**Oral**						
Exposure / Dose	accidental		S	accidental		S
**Intake**						
Exposure per task / Acute Dose	0,933333333	mg/kg bw	S	3,133473896	mg/kg bw	O
Exposure per day / Chronic Dose	0,030684932	mg/kg bw/day	O	0,10301832	mg/kg bw/day	O

**Disposal**	Task D: not applicable					

U = Undefined, D = Default, S = Set, O = Output, REM = Remark, not used in calculation

Scenario 6: cleaning/disinfection: textiles (e.g. jacket, sofa) using (ready for-use) spray

Scenario description				Comments
Name of product	disinfection spray		S	
Physical state product (liquid/solid)	liquid		S	
Density product	1	g/cm^3	D	
Concentration of active substance in product	1	%	S	*Frame Formulation*
Concentration of active substance in product (mg/l)	10000	mg/l	O	
User	Consumer		S	
Bystander	Children		S	
Temperature	20	°C	D	*room temperature*
**Mixing & Loading**	Task A: not applicable			
**Application**	Task B: spraying onto surface + deposit time			
Number of tasks per year	12	tasks/yr	S	*once a month*
Duration of task	10	min/task	S	*incl. deposit time*
Surface (treated per task)	1	m^2/task	S	
Quantity of product used per m^2	50	ml/m^2	D	
Quantity of product used per task	50	ml/task	O	
Quantity of active substance used per task	500	mg/task	O	
Model inhalation exposure	Exposure to aerosols / Instantaneous release			
Room volume	30	m^3	S	*Room*
Model dermal exposure	Direct dermal contact			
Contact area between product and skin	0,258033333	m^2	S	*Forearms, Hands, 33% Head, 10% Lower legs*
Model oral exposure	accidental			
**Post application phase**	Task C: residence time			
Number of tasks per year	12	tasks/yr	S	*once a month*
Duration of task	50	min/task	S	
Quantity of active substance used per task	500	mg/task	O	
Model inhalation exposure	Exposure to vapour / Instantaneous release (limited to vapour pressure)			
Room volume	30	m^3	S	*Room*
Model dermal exposure	Direct dermal contact			
Contact area between product and skin	0,319	m^2	S	*Upper extremities (Jacket with T-shirt)*
Contact area between product and skin children	0,074575	m^2	S	*Arms, Hands children*
Model oral exposure	accidental			
**Disposal**	Task D: not applicable			

Summary Results Exposure

	User	Consumer	Bystander	Children
Highest potential exposure acute (all amount used is absorbed)	8,333333333	mg/kg bw	60,24096386	mg/kg bw
Highest potential exposure chronic (all amount used is absorbed)	0,273972603	mg/kg bw/day	1,980524839	mg/kg bw/day
Highest potential concentration in air	16,66666667	mg/m^3	16,66666667	mg/m^3
Inhalation acute	0,173611111	mg/kg bw	0,363955823	mg/kg bw
Inhalation chronic	0,005707763	mg/kg bw/day	0,011965671	mg/kg bw/day
Dermal acute	8,333333333	mg/kg bw	8,984939759	mg/kg bw
Dermal chronic	0,273972603	mg/kg bw/day	0,29539528	mg/kg bw/day
Oral acute	accidental		accidental	
Oral chronic	accidental		accidental	
Intake acute	8,333333333	mg/kg bw	9,348895582	mg/kg bw
Daily intake chronic	0,273972603	mg/kg bw/day	0,307360951	mg/kg bw/day

U = Undefined, D = Default, S = Set, O = Output, REM = Remark, not used in calculation

Scenario 6: cleaning/disinfection: textiles (e.g. jacket, sofa) using (ready for-use) spray

Results / Output

**Mixing & Loading**	Task A: not applicable					

**Application**	Task B: spraying onto surface + deposit time					
	**Primary exposure**	**User**		**Secondary exposure**	**Bystander**	
**Inhalation**						
Concentration of potential exposure	16,66666667	mg/m^3	O	16,66666667	mg/m^3	O
Inhalation	0,231481481	mg/min	O	0,06712963	mg/min	O
Absorption per task	1,736111111	mg/task	O	0,503472222	mg/task	O
Exposure per task / Acute Dose	0,028935185	mg/kg bw	O	0,060659304	mg/kg bw	O
Exposure per day / Chronic Dose	0,000951294	mg/kg bw/day	O	0,001994278	mg/kg bw/day	O
**Dermal**						
Contact area between product and skin	0,258033333	m^2	S			
Volume of contact	25,80333333	ml	O			
Quantity of contact (mg)	258,0333333	mg	O			
Dermal load (mg/cm^2)	0,1	mg/cm^2	O			
Absorption (mg)	258,0333333	mg	O			
Exposure per task / Acute Dose	4,300555556	mg/kg bw	O	no		S
Exposure per day / Chronic Dose	0,141388128	mg/kg bw/day	O	no		S
**Oral**						
Exposure / Dose	accidental		S	accidental		S
**Intake**						
Exposure per task / Acute Dose	4,329490741	mg/kg bw	O	0,060659304	mg/kg bw	O
Exposure per day / Chronic Dose	0,142339422	mg/kg bw/day	O	0,001994278	mg/kg bw/day	O

**Post application phase**	Task C: residence time					
	**Primary exposure**	**User**		**Secondary exposure**	**Bystander**	
**Inhalation**						
Concentration of potential exposure	16,66666667	mg/m^3	O	16,66666667	mg/m^3	O
Inhalation	0,231481481	mg/min	O	0,06712963	mg/min	O
Absorption per task	8,680555556	mg/task	O	2,517361111	mg/task	O
Exposure per task / Acute Dose	0,144675926	mg/kg bw	O	0,303296519	mg/kg bw	O
Exposure per day / Chronic Dose	0,004756469	mg/kg bw/day	O	0,009971392	mg/kg bw/day	O
**Dermal**						
Contact area between product and skin	0,319	m^2	S	0,074575	m^2	S
Volume of contact	31,9	ml	O	7,4575	ml	O
Quantity of contact (mg)	319	mg	O	74,575	mg	O
Dermal load (mg/cm^2)	0,1	mg/cm^2	O	0,1	mg/cm^2	O
Absorption (mg)	319	mg	O	74,575	mg	O
Exposure per task / Acute Dose	5,316666667	mg/kg bw	O	8,984939759	mg/kg bw	O
Exposure per day / Chronic Dose	0,174794521	mg/kg bw/day	O	0,29539528	mg/kg bw/day	O
**Oral**						
Exposure / Dose	accidental		S	accidental		S
**Intake**						
Exposure per task / Acute Dose	5,461342593	mg/kg bw	O	9,288236278	mg/kg bw	O
Exposure per day / Chronic Dose	0,179550989	mg/kg bw/day	O	0,305366672	mg/kg bw/day	O

**Disposal**	Task D: not applicable					

Scenario 7: cleaning/disinfection: surfaces using spray (dilution of liquid concentrate)

Scenario description

				Comments
Name of product	disinfection cleaner		S	
Physical state product (liquid/solid)	liquid		S	
Density product	1	g/cm^3	D	
Concentration of active substance in product (concentrate)	1	%	S	*Frame Formulation*
Concentration of active substance in product (concentrate) (mg/l)	10000	mg/l	O	
User	Consumer		S	
Bystander	Children		S	
Temperature	20	°C	D	*room temperature*

Mixing & Loading

	Task A: simple dilution with water in spray bottles			
Number of tasks per year	52	tasks/yr	S	*once a week*
Duration of task	1,333333333	min/task	S	*80 sec. [mixing spray TNsG 2002 p. 252]*
Volume of product used per task	1000	ml/task	S	*Frame Formulation 100-200 ml/l*
Quantity of water used per task	4	L/task	S	*Gesamtvolumen 5 Liter*
Quantity of product used per task	1000	g/task	O	
Quantity of active substance used per task	10000	mg/task	O	
Concentration of active substance in ready-for-use solution	2000	mg/l	S	
Model inhalation exposure	Exposure to vapour / Fugacity concept (equilibrium between ready-for-use solution and air, limited to vapour pressure)			
Room volume	1	m^3	S	*cloud around user*
REM Evaporation from mixture, release area concentrate	*0,002*	*m^2*	*D*	*bottle diameter 5 cm [TNsG 2002 p. 252]*
REM Evaporation from mixture, release area ready-for-use solution	*0,001963495*	*m^2*	*S*	*bottle diameter 5 cm*
Model dermal exposure	Direct dermal contact			
Contact area between Product (concentrate) and skin	0,0168	m^2	S	*Fingertips*
Model oral exposure	accidental			

Application

	Task B: spraying onto surface + deposit time			
Number of tasks per year	52	tasks/yr	S	*once a week*
Duration of task	30	min/task	S	
Surface (treated per task)	20	m^2/task	S	
Quantity of ready-for-use solution used per m^2	50	ml/m^2	D	
Quantity of ready-for-use solution used per task	1000	ml/task	O	
Quantity of active substance used per task	2000	mg/task	O	
Concentration of active substance in ready-for-use solution	2000	mg/l	S	
Model inhalation exposure	Exposure to aerosols / Instantaneous release			
Room volume	15	m^3	S	*Kitchen (Terrace)*
Model dermal exposure	Direct dermal contact			
Contact area between ready-for-use solution and skin	0,258033333	m^2	S	*Forearms, Hands, 33% Head, 10% Lower legs*
Model oral exposure	accidental			

Post application phase

	Task C: residence time			
Number of tasks per year	52	tasks/yr	S	*once a week*
Duration of task	90	min/task	S	*ConsExpo: 120 min total inhalative exposure liquid cleaner*
Quantity of active substance used per task	2000	mg/task	O	
Concentration of active substance in ready-for-use solution	2000	mg/l	S	
Model inhalation exposure	Exposure to vapour / Instantaneous release (limited to vapour pressure)			
Room volume	15	m^3	S	*Kitchen (Terrace)*
Model dermal exposure	Direct dermal contact			
Contact area between ready-for-use solution and skin	0,042	m^2	S	*50% Hands*
Contact area between ready-for-use solution and skin children	0,01040125	m^2	S	*50% Hands Children*
Model oral exposure	accidental			

Disposal

	Task D: excessive ready-for-use solution is disposed to the main drainage			
Number of tasks per year	52	tasks/yr	S	*once a week*
Duration of task	1	min/task	S	
Volume of ready-for-use solution disposed per task	4	L/task	S	
Concentration of active substance in ready-for-use solution	2000	mg/l	S	
Quantity of active substance disposed per task	8000	mg/task	O	
Model inhalation exposure	Exposure to vapour / Fugacity concept (equilibrium between ready-for-use solution and air, limited to vapour pressure)			
Room volume	1	m^3	S	*cloud around user*
REM Evaporation from mixture, release area ready-for-use solution	*0,070685835*	*m^2*	*S*	*bucket diameter 30 cm*
Model dermal exposure	Direct dermal contact			
Contact area between ready-for-use solution and skin	0,198	m^2	S	*Hands, Forearms*
Model oral exposure	accidental			

Summary Results Exposure

	**User**	**Consumer**		**Bystander**	**Children**	
Highest potential exposure acute (all amount used is absorbed)		166,6666667	mg/kg bw		1204,819277	mg/kg bw
Highest potential exposure chronic (all amount used is absorbed)		23,74429224	mg/kg bw/day		171,6454861	mg/kg bw/day
Highest potential concentration in air		133,3333333	mg/m^3		133,3333333	mg/m^3
Inhalation acute		2,777967721	mg/kg bw		5,823691367	mg/kg bw
Inhalation chronic		0,395765264	mg/kg bw/day		0,829676578	mg/kg bw/day
Dermal acute		1,940111111	mg/kg bw		0,25063253	mg/kg bw
Dermal chronic		0,276399391	mg/kg bw/day		0,035706552	mg/kg bw/day
Oral acute	accidental			accidental		
Oral chronic	accidental			accidental		
Intake acute		4,718078832	mg/kg bw		6,074323897	mg/kg bw
Daily intake chronic		0,672164656	mg/kg bw/day		0,865383131	mg/kg bw/day

U = Undefined, D = Default, S = Set, O = Output, REM = Remark, not used in calculation

Scenario 7: cleaning/disinfection: surfaces using spray (dilution of liquid concentrate)

Results / Output

**Mixing & Loading**	Task A: simple dilution with water in spray bottles					
	**Primary exposure**	**User**		**Secondary exposure**	**Bystander**	
**Inhalation**						
Concentration of potential exposure	0,512847002	mg/m^3	O	0,512847002	mg/m^3	O
Inhalation	0,007122875	mg/min	O	0,002065634	mg/min	O
Absorption per task	0,007122875	mg/task	O	0,002065634	mg/task	O
Exposure per task / Acute Dose	0,000118715	mg/kg bw	O	0,000248872	mg/kg bw	O
Exposure per day / Chronic Dose	1,69128E-05	mg/kg bw/day	O	3,54557E-05	mg/kg bw/day	O
**Dermal**						
Contact area between product and skin	0,0168	m^2	S			
Volume of contact	1,68	ml	O			
Quantity of contact (mg)	16,8	mg	O			
Dermal load (mg/cm^2)	0,1	mg/cm^2	O			
Absorption (mg)	16,8	mg	O			
Exposure per task / Acute Dose	0,28	mg/kg bw	O	no		S
Exposure per day / Chronic Dose	0,039890411	mg/kg bw/day	O	no		S
**Oral**						
Exposure / Dose	accidental		S	accidental		S
**Intake**						
Exposure per task / Acute Dose	0,280118715	mg/kg bw	O	0,000248872	mg/kg bw	O
Exposure per day / Chronic Dose	0,039907324	mg/kg bw/day	O	3,54557E-05	mg/kg bw/day	O

**Application**	Task B: spraying onto surface + deposit time					
	**Primary exposure**	**User**		**Secondary exposure**	**Bystander**	
**Inhalation**						
Concentration of potential exposure	133,3333333	mg/m^3	O	133,3333333	mg/m^3	O
Inhalation	1,851851852	mg/min	O	0,537037037	mg/min	O
Absorption per task	41,66666667	mg/task	O	12,08333333	mg/task	O
Exposure per task / Acute Dose	0,694444444	mg/kg bw	O	1,455823293	mg/kg bw	O
Exposure per day / Chronic Dose	0,098934551	mg/kg bw/day	O	0,207404962	mg/kg bw/day	O
**Dermal**						
Contact area between ready-for-use solution and skin	0,258033333	m^2	S			
Volume of contact	25,80333333	ml	O			
Quantity of contact (mg)	51,60666667	mg	O			
Dermal load (mg/cm^2)	0,02	mg/cm^2	O			
Absorption (mg)	51,60666667	mg	O			
Exposure per task / Acute Dose	0,860111111	mg/kg bw	O	no		S
Exposure per day / Chronic Dose	0,122536377	mg/kg bw/day	O	no		S
**Oral**						
Exposure / Dose	accidental		S	accidental		S
**Intake**						
Exposure per task / Acute Dose	1,554555556	mg/kg bw	O	1,455823293	mg/kg bw	O
Exposure per day / Chronic Dose	0,221470928	mg/kg bw/day	O	0,207404962	mg/kg bw/day	O

**Post application phase**	Task C: residence time					
	**Primary exposure**	**User**		**Secondary exposure**	**Bystander**	
**Inhalation**						
Concentration of potential exposure	133,3333333	mg/m^3	O	133,3333333	mg/m^3	O
Inhalation	1,851851852	mg/min	O	0,537037037	mg/min	O
Absorption per task	125	mg/task	O	36,25	mg/task	O
Exposure per task / Acute Dose	2,083333333	mg/kg bw	O	4,36746988	mg/kg bw	O
Exposure per day / Chronic Dose	0,296803653	mg/kg bw/day	O	0,622214887	mg/kg bw/day	O
**Dermal**						
Contact area between ready-for-use solution and skin	0,042	m^2	S	0,01040125	m^2	S
Volume of contact	4,2	ml	O	1,040125	ml	O
Quantity of contact (mg)	8,4	mg	O	2,08025	mg	O
Dermal load (mg/cm^2)	0,02	mg/cm^2	O	0,02	mg/cm^2	O
Absorption (mg)	8,4	mg	O	2,08025	mg	O
Exposure per task / Acute Dose	0,14	mg/kg bw	O	0,25063253	mg/kg bw	O
Exposure per day / Chronic Dose	0,019945205	mg/kg bw/day	O	0,035706552	mg/kg bw/day	O
**Oral**						
Exposure / Dose	accidental		S	accidental		S
**Intake**						
Exposure per task / Acute Dose	2,223333333	mg/kg bw	O	4,61810241	mg/kg bw	O
Exposure per day / Chronic Dose	0,316748858	mg/kg bw/day	O	0,657921439	mg/kg bw/day	O

**Disposal**	Task D: excessive ready-for-use solution is disposed to the main drainage					
	**Primary exposure**	**User**		**Secondary exposure**	**Bystander**	
**Inhalation**						
Concentration of potential exposure	0,410277602	mg/m^3	O	0,410277602	mg/m^3	O
Inhalation	0,0056983	mg/min	O	0,001652507	mg/min	O
Absorption per task	0,004273725	mg/task	O	0,00123938	mg/task	O
Exposure per task / Acute Dose	7,12288E-05	mg/kg bw	O	0,000149323	mg/kg bw	O
Exposure per day / Chronic Dose	1,01477E-05	mg/kg bw/day	O	2,12734E-05	mg/kg bw/day	O
**Dermal**						
Contact area between ready-for-use solution and skin	0,198	m^2	S			
Volume of contact	19,8	ml	O			
Quantity of contact (mg)	39,6	mg	O			
Dermal load (mg/cm^2)	0,02	mg/cm^2	O			
Absorption (mg)	39,6	mg	O			
Exposure per task / Acute Dose	0,66	mg/kg bw	O	no		S
Exposure per day / Chronic Dose	0,094027397	mg/kg bw/day	O	no		S
**Oral**						
Exposure / Dose	accidental		S	accidental		S
**Intake**						
Exposure per task / Acute Dose	0,660071229	mg/kg bw	O	0,000149323	mg/kg bw	O
Exposure per day / Chronic Dose	0,094037545	mg/kg bw/day	O	2,12734E-05	mg/kg bw/day	O

Scenario 8: cleaning/disinfection: surfaces using (ready-for-use) spray (incl. wiping of surface)

Scenario description				*Comments*
Name of product	disinfection cleaner		S	
Physical state product (liquid/solid)	liquid		S	
Density product	1	g/cm^3	D	
Concentration of active substance in product	1	%	S	*Frame Formulation*
Concentration of active substance in product (mg/l)	10000	mg/l	O	
User	Consumer		S	
Bystander	Children		S	
Temperature	20	°C	D	*room temperature*
**Mixing & Loading**	Task A: not applicable			
**Application I**	Task B: spraying onto surface + deposit time			
Number of tasks per year	365	tasks/yr	S	*once per day, max [AISE (2002) cited in TGD (2003)]*
Duration of task	10	min/task	S	*max 10 min [AISE (2002) cited in TGD (2003)]*
Surface (treated per task)	0,6	m^2/task	S	
Quantity of product used per m^2	50	ml/m^2	D	
Quantity of product used per task	30	ml/task	O	*max 30 g [AISE (2002) cited in TGD (2003)]*
Quantity of active substance used per task	300	mg/task	O	
Model inhalation exposure	Exposure to aerosols / Instantaneous release			
Room volume	10	m^3	S	*Bathroom*
Model dermal exposure	Direct dermal contact			
Contact area between product and skin	0,258033333	m^2	S	*Forearms, Hands, 33% Head, 10% Lower legs*
Model oral exposure	accidental			
**Post application phase I**	Task C: residence time after spraying			
Number of tasks per year	365	tasks/yr	S	*once per day, max [AISE (2002) cited in TGD (2003)]*
Duration of task	10	min/task	S	
Quantity of active substance used per task	300	mg/task	O	
Model inhalation exposure	Exposure to vapour / Instantaneous release (limited to vapour pressure)			
Room volume	10	m^3	S	*Bathroom*
Model dermal exposure	Direct dermal contact			
Contact area between product and skin	0,042	m^2	D	*50% Hands*
Contact area between product and skin children	0,01040125	m^2	D	*50% Hands Children*
Model oral exposure	accidental			
**Application II**	Task D: wiping of surface			
Number of tasks per year	365	tasks/yr	S	*once per day, max [AISE (2002) cited in TGD (2003)]*
Duration of task	10	min/task	S	
Surface (treated per task)	0,6	m^2/task	S	
Quantity of active substance used per task	300	mg/task	O	
Model inhalation exposure	Exposure to vapour / Instantaneous release (limited to vapour pressure)			
Room volume	10	m^3	S	*Bathroom*
Model dermal exposure	Direct dermal contact			
Contact area between product and skin	0,198	m^2	S	*Forearms, Hands*
Model oral exposure	accidental			
**Post application phase II**	Task E: residence time after wiping			
Number of tasks per year	365	tasks/yr	S	*once per day, max [AISE (2002) cited in TGD (2003)]*
Duration of task	90	min/task	S	*ConsExpo: 120 min total inhalative exposure liquid cleaner*
Quantity of active substance used per task	300	mg/task	O	
Model inhalation exposure	Exposure to vapour / Instantaneous release (limited to vapour pressure)			
Room volume	10	m^3	S	*Bathroom*
Model dermal exposure	Direct dermal contact			
Contact area between product and skin	0,042	m^2	S	*50% Hands*
Contact area between product and skin children	0,01040125	m^2	S	*50% Hands Children*
Model oral exposure	accidental			
**Disposal**	Task F: not applicable			

Summary Results Exposure

	User		Consumer	Bystander		Children
Highest potential exposure acute (all amount used is absorbed)		5	mg/kg bw		36,14457831	mg/kg bw
Highest potential exposure chronic (all amount used is absorbed)		5	mg/kg bw/day		36,14457831	mg/kg bw/day
Highest potential concentration in air		30	mg/m^3		30	mg/m^3
Inhalation acute		0,625	mg/kg bw		1,310240964	mg/kg bw
Inhalation chronic		0,625	mg/kg bw/day		1,310240964	mg/kg bw/day
Dermal acute		5	mg/kg bw		2,506325301	mg/kg bw
Dermal chronic		5	mg/kg bw/day		2,506325301	mg/kg bw/day
Oral acute	accidental			accidental		
Oral chronic	accidental			accidental		
Intake acute		5	mg/kg bw		3,816566265	mg/kg bw
Daily intake chronic		5	mg/kg bw/day		3,816566265	mg/kg bw/day

U = Undefined, D = Default, S = Set, O = Output, REM = Remark, not used in calculation

Scenario 8: cleaning/disinfection: surfaces using (ready-for-use) spray (incl. wiping of surface)

Results / Output

Mixing & Loading	Task A: not applicable					

**Application I**	Task B: spraying onto surface + deposit time					
	**Primary exposure**	**User**		**Secondary exposure**	**Bystander**	
**Inhalation**						
Concentration of potential exposure	30	mg/m^3	O	30	mg/m^3	O
Inhalation	0,416666667	mg/min	O	0,120833333	mg/min	O
Absorption per task	3,125	mg/task	O	0,90625	mg/task	O
Exposure per task / Acute Dose	0,052083333	mg/kg bw	O	0,109186747	mg/kg bw	O
Exposure per day / Chronic Dose	0,052083333	mg/kg bw/day	O	0,109186747	mg/kg bw/day	O
**Dermal**						
Contact area between product and skin	0,258033333	m^2	S			
Volume of contact	25,80333333	ml	O			
Quantity of contact (mg)	258,0333333	mg	O			
Dermal load (mg/cm^2)	0,1	mg/cm^2	O			
Absorption (mg)	258,0333333	mg	O			
Exposure per task / Acute Dose	4,300555556	mg/kg bw	O	no		S
Exposure per day / Chronic Dose	4,300555556	mg/kg bw/day	O	no		S
**Oral**						
Exposure / Dose	accidental		S	accidental		S
**Intake**						
Exposure per task / Acute Dose	4,352638889	mg/kg bw	O	0,109186747	mg/kg bw	O
Exposure per day / Chronic Dose	4,352638889	mg/kg bw/day	O	0,109186747	mg/kg bw/day	O

**Post application phase I**	Task C: residence time after spraying					
	**Primary exposure**	**User**		**Secondary exposure**	**Bystander**	
**Inhalation**						
Concentration of potential exposure	30	mg/m^3	O	30	mg/m^3	O
Inhalation	0,416666667	mg/min	O	0,120833333	mg/min	O
Absorption per task	3,125	mg/task	O	0,90625	mg/task	O
Exposure per task / Acute Dose	0,052083333	mg/kg bw	O	0,109186747	mg/kg bw	O
Exposure per day / Chronic Dose	0,052083333	mg/kg bw/day	O	0,109186747	mg/kg bw/day	O
**Dermal**						
Contact area between product and skin	0,042	m^2	D	0,01040125	m^2	D
Volume of contact	4,2	ml	O	1,040125	ml	O
Quantity of contact (mg)	42	mg	O	10,40125	mg	O
Dermal load (mg/cm^2)	0,1	mg/cm^2	O	0,1	mg/cm^2	O
Absorption (mg)	42	mg	O	10,40125	mg	O
Exposure per task / Acute Dose	0,7	mg/kg bw	O	1,253162651	mg/kg bw	O
Exposure per day / Chronic Dose	0,7	mg/kg bw/day	O	1,253162651	mg/kg bw/day	O
**Oral**						
Exposure / Dose	accidental		S	accidental		S
**Intake**						
Exposure per task / Acute Dose	0,752083333	mg/kg bw	O	1,362349398	mg/kg bw	O
Exposure per day / Chronic Dose	0,752083333	mg/kg bw/day	O	1,362349398	mg/kg bw/day	O

**Application II**	Task D: wiping of surface					
	**Primary exposure**	**User**		**Secondary exposure**	**Bystander**	
**Inhalation**						
Concentration of potential exposure	30	mg/m^3	O	30	mg/m^3	O
Inhalation	0,416666667	mg/min	O	0,120833333	mg/min	O
Absorption per task	3,125	mg/task	O	0,90625	mg/task	O
Exposure per task / Acute Dose	0,052083333	mg/kg bw	O	0,109186747	mg/kg bw	O
Exposure per day / Chronic Dose	0,052083333	mg/kg bw/day	O	0,109186747	mg/kg bw/day	O
**Dermal**						
Contact area between product and skin	0,198	m^2	O			
Volume of contact	19,8	ml	O			
Quantity of contact (mg)	198	mg	O			
Dermal load (mg/cm^2)	0,1	mg/cm^2	O			
Absorption (mg)	198	mg	O			
Exposure per task / Acute Dose	3,3	mg/kg bw	O	no		S
Exposure per day / Chronic Dose	3,3	mg/kg bw/day	O	no		S
**Oral**						
Exposure / Dose	accidental		S	accidental		S
**Intake**						
Exposure per task / Acute Dose	3,352083333	mg/kg bw	O	0,109186747	mg/kg bw	O
Exposure per day / Chronic Dose	3,352083333	mg/kg bw/day	O	0,109186747	mg/kg bw/day	O

**Post application phase II**	Task E: residence time after wiping					
	**Primary exposure**	**User**		**Secondary exposure**	**Bystander**	
**Inhalation**						
Concentration of potential exposure	30	mg/m^3	O	30	mg/m^3	O
Inhalation	0,416666667	mg/min	O	0,120833333	mg/min	O
Absorption per task	28,125	mg/task	O	8,15625	mg/task	O
Exposure per task / Acute Dose	0,46875	mg/kg bw	O	0,982680723	mg/kg bw	O
Exposure per day / Chronic Dose	0,46875	mg/kg bw/day	O	0,982680723	mg/kg bw/day	O
**Dermal**						
Contact area between product and skin	0,042	m^2	S	0,01040125	m^2	S
Volume of contact	4,2	ml	O	1,040125	ml	O
Quantity of contact (mg)	42	mg	O	10,40125	mg	O
Dermal load (mg/cm^2)	0,1	mg/cm^2	O	0,1	mg/cm^2	O
Absorption (mg)	42	mg	O	10,40125	mg	O
Exposure per task / Acute Dose	0,7	mg/kg bw	O	1,253162651	mg/kg bw	O
Exposure per day / Chronic Dose	0,7	mg/kg bw/day	O	1,253162651	mg/kg bw/day	O
**Oral**						
Exposure / Dose	accidental		S	accidental		S
**Intake**						
Exposure per task / Acute Dose	1,16875	mg/kg bw	O	2,235843373	mg/kg bw	O
Exposure per day / Chronic Dose	1,16875	mg/kg bw/day	O	2,235843373	mg/kg bw/day	O

Disposal	Task F: not applicable					

U = Undefined, D = Default, S = Set, O = Output, REM = Remark, not used in calculation

Scenario 9: cleaning/disinfection: surfaces using (ready-for-use) spray (incl. washing-up of surface)

Scenario description

				Comments
Name of product	disinfection cleaner		S	
Physical state product (liquid/solid)	liquid		S	
Density product	1	g/cm^3	D	
Concentration of active substance in product	1	%	S	*Frame Formulation*
Concentration of active substance in product (mg/l)	10000	mg/l	O	
User	Consumer		S	
Bystander	Children		S	
Temperature	20	°C	D	*room temperature*
**Mixing & Loading**	Task A: not applicable			
**Application I**	Task B: spraying onto surface + deposit time			
Number of tasks per year	365	tasks/yr	S	*once per day, max [AISE (2002) cited in TGD (2003)]*
Duration of task	10	min/task	S	*max 10 min [AISE (2002) cited in TGD (2003)]*
Surface (treated per task)	0,6	m^2/task	S	
Quantity of product used per m^2	50	ml/m^2	D	
Quantity of product used per task	30	ml/task	O	*max 30 g [AISE (2002) cited in TGD (2003)]*
Quantity of active substance used per task	300	mg/task	O	
Model inhalation exposure	Exposure to aerosols / Instantaneous release			
Room volume	10	m^3	S	*Bathroom*
Model dermal exposure	Direct dermal contact			
Contact area between product and skin	0,258033333	m^2	S	*Forearms, Hands, 33% Head, 10% Lower legs*
Model oral exposure	accidental			
**Post application phase I**	Task C: residence time			
Number of tasks per year	365	tasks/yr	S	*once per day, max [AISE (2002) cited in TGD (2003)]*
Duration of task	10	min/task	S	
Quantity of active substance used per task	300	mg/task	O	
Model inhalation exposure	Exposure to vapour / Instantaneous release (limited to vapour pressure)			
Room volume	10	m^3	S	*Bathroom*
Model dermal exposure	Direct dermal contact			
Contact area between product and skin	0,042	m^2	S	*50% Hands*
Contact area between product and skin children	0,01040125	m^2	S	*50% Hands Children*
Model oral exposure	accidental			
**Application II**	Task D: wash-up of surface			
Number of tasks per year	365	tasks/yr	S	*once per day, max [AISE (2002) cited in TGD (2003)]*
Duration of task	10	min/task	S	
Surface (treated per task)	0,6	m^2/task	S	
Quantity of active substance used per task	300	mg/task	O	
Model inhalation exposure	Exposure to vapour / Instantaneous release (limited to vapour pressure)			
Room volume	10	m^3	S	*Bathroom*
Model dermal exposure	Direct dermal contact			
Contact area between product and skin	0,198	m^2	S	*Forearms, Hands*
Model oral exposure	accidental			
**Post application phase II**	Task E: not applicable			
**Disposal**	Task F: not applicable			

Summary Results Exposure

	User	Consumer	Bystander	Children
Highest potential exposure acute (all amount used is absorbed)	5	mg/kg bw	36,14457831	mg/kg bw
Highest potential exposure chronic (all amount used is absorbed)	5	mg/kg bw/day	36,14457831	mg/kg bw/day
Highest potential concentration in air	30	mg/m^3	30	mg/m^3
Inhalation acute	0,15625	mg/kg bw	0,327560241	mg/kg bw
Inhalation chronic	0,15625	mg/kg bw/day	0,327560241	mg/kg bw/day
Dermal acute	5	mg/kg bw	1,253162651	mg/kg bw
Dermal chronic	5	mg/kg bw/day	1,253162651	mg/kg bw/day
Oral acute	accidental		accidental	
Oral chronic	accidental		accidental	
Intake acute	5	mg/kg bw	1,580722892	mg/kg bw
Daily intake chronic	5	mg/kg bw/day	1,580722892	mg/kg bw/day

U = Undefined, D = Default, S = Set, O = Output, REM = Remark, not used in calculation

Scenario 9: cleaning/disinfection: surfaces using (ready-for-use) spray (incl. washing-up of surface)

Results / Output

**Mixing & Loading**	Task A: not applicable

**Application I**	Task B: spraying onto surface + deposit time					
	**Primary exposure**	**User**		**Secondary exposure**	**Bystander**	
**Inhalation**						
Concentration of potential exposure	30	mg/m³	O	30	mg/m³	O
Inhalation	0,416666667	mg/min	O	0,120833333	mg/min	O
Absorption per task	3,125	mg/task	O	0,90625	mg/task	O
Exposure per task / Acute Dose	0,052083333	mg/kg bw	O	0,109186747	mg/kg bw	O
Exposure per day / Chronic Dose	0,052083333	mg/kg bw/day	O	0,109186747	mg/kg bw/day	O
**Dermal**						
Contact area between product and skin	0,258033333	m²	S			
Volume of contact	25,80333333	ml	O			
Quantity of contact (mg)	258,0333333	mg	O			
Dermal load (mg/cm²)	0,1	mg/cm²	O			
Absorption (mg)	258,0333333	mg	O			
Exposure per task / Acute Dose	4,300555556	mg/kg bw	O	no		S
Exposure per day / Chronic Dose	4,300555556	mg/kg bw/day	O	no		S
**Oral**						
Exposure / Dose	accidental		S	accidental		S
**Intake**						
Exposure per task / Acute Dose	4,352638889	mg/kg bw	O	0,109186747	mg/kg bw	O
Exposure per day / Chronic Dose	4,352638889	mg/kg bw/day	O	0,109186747	mg/kg bw/day	O

**Post application phase I**	Task C: residence time					
	**Primary exposure**	**User**		**Secondary exposure**	**Bystander**	
**Inhalation**						
Concentration of potential exposure	30	mg/m³	O	30	mg/m³	O
Inhalation	0,416666667	mg/min	O	0,120833333	mg/min	O
Absorption per task	3,125	mg/task	O	0,90625	mg/task	O
Exposure per task / Acute Dose	0,052083333	mg/kg bw	O	0,109186747	mg/kg bw	O
Exposure per day / Chronic Dose	0,052083333	mg/kg bw/day	O	0,109186747	mg/kg bw/day	O
**Dermal**						
Contact area between product and skin	0,042	m²	S	0,01040125	m²	S
Volume of contact	4,2	ml	O	1,040125	ml	O
Quantity of contact (mg)	42	mg	O	10,40125	mg	O
Dermal load (mg/cm²)	0,1	mg/cm²	O	0,1	mg/cm²	O
Absorption (mg)	42	mg	O	10,40125	mg	O
Exposure per task / Acute Dose	0,7	mg/kg bw	O	1,253162651	mg/kg bw	O
Exposure per day / Chronic Dose	0,7	mg/kg bw/day	O	1,253162651	mg/kg bw/day	O
**Oral**						
Exposure / Dose	accidental		S	accidental		S
**Intake**						
Exposure per task / Acute Dose	0,752083333	mg/kg bw	O	1,362349398	mg/kg bw	O
Exposure per day / Chronic Dose	0,752083333	mg/kg bw/day	O	1,362349398	mg/kg bw/day	O

**Application II**	Task D: wash-up of surface					
	**Primary exposure**	**User**		**Secondary exposure**	**Bystander**	
**Inhalation**						
Concentration of potential exposure	30	mg/m³	O	30	mg/m³	O
Inhalation	0,416666667	mg/min	O	0,120833333	mg/min	O
Absorption per task	3,125	mg/task	O	0,90625	mg/task	O
Exposure per task / Acute Dose	0,052083333	mg/kg bw	O	0,109186747	mg/kg bw	O
Exposure per day / Chronic Dose	0,052083333	mg/kg bw/day	O	0,109186747	mg/kg bw/day	O
**Dermal**						
Contact area between product and skin	0,198	m²	O			
Volume of contact	19,8	ml	O			
Quantity of contact (mg)	198	mg	O			
Dermal load (mg/cm²)	0,1	mg/cm²	O			
Absorption (mg)	198	mg	O			
Exposure per task / Acute Dose	3,3	mg/kg bw	O	no		S
Exposure per day / Chronic Dose	3,3	mg/kg bw/day	O	no		S
**Oral**						
Exposure / Dose	accidental		S	accidental		S
**Intake**						
Exposure per task / Acute Dose	3,352083333	mg/kg bw	O	0,109186747	mg/kg bw	O
Exposure per day / Chronic Dose	3,352083333	mg/kg bw/day	O	0,109186747	mg/kg bw/day	O

**Post application phase II**	Task E: not applicable

**Disposal**	Task F: not applicable

Scenario 10: cleaning/disinfection: disinfection wipe (small surfaces)

Scenario description				*Comments*
Name of product	disinfection wipe		S	
Physical state product (liquid/solid)	liquid		S	
Concentration of active substance in product	1	g/wipe	S	*Frame Formulation*
User	Consumer		S	
Bystander	Children		S	
Temperature	20	°C	D	*room temperature*
**Mixing & Loading**	Task A: not applicable			
**Application**	Task B: disinfection: wiping of surface			
Number of tasks per year	365	tasks/yr	S	*once a day*
Duration of task	2	min/task	S	*ConsExpo*
Quantity of wipes used per task	1	wipes/task	S	
Quantity of active substance used per task	1000	mg/task	O	
Model inhalation exposure	Exposure to vapour / Instantaneous release (limited to vapour pressure)			
Room volume	1	m^3	S	*cloud around user*
REM Surface (treated per task), Release area	*0,0682*	*m^2/task*	*S*	*toilet seat, doorknob*
Model dermal exposure	Direct dermal contact			
Contact area between product and skin	0,042	m^2	S	*50% Hands*
Contact amount between active substance and skin	100	mg/task	S	*10% of whole amount used*
Model oral exposure	accidental			
**Post application phase**	Task C: residence time			
Number of tasks per year	365	tasks/yr	S	*once a day*
Duration of task	10	min/task	S	
Quantity of wipes used per task	1	wipes/task	S	
Quantity of active substance used per task	1000	mg/task	O	
Model inhalation exposure	Exposure to vapour / Instantaneous release (limited to vapour pressure)			
Room volume	2,5	m^3	S	*Toilet*
REM Surface (treated per task), Release area	*0,0682*	*m^2/task*	*S*	*toilet seat, doorknob*
Model dermal exposure	Direct dermal contact			
Contact area between treated surface and skin	0,042	m^2	S	*50% Hands*
Contact area between treated surface and skin children	0,01040125	m^2	S	*50% Hands Children*
Contact amount between active substance and skin	100	mg/task	S	*10% of whole amount used, residuals on surface*
Model oral exposure	accidental			
**Disposal**	Task D: not applicable			

Summary Results Exposure

	User	Consumer		Bystander	Children	
Highest potential exposure acute (all amount used is absorbed)		16,66666667	mg/kg bw		120,4819277	mg/kg bw
Highest potential exposure chronic (all amount used is absorbed)		16,66666667	mg/kg bw/day		120,4819277	mg/kg bw/day
Highest potential concentration in air		205,1493218	mg/m^3		205,1493218	mg/m^3
Inhalation acute		0,42739442	mg/kg bw		0,895983484	mg/kg bw
Inhalation chronic		0,42739442	mg/kg bw/day		0,895983484	mg/kg bw/day
Dermal acute		3,333333333	mg/kg bw		12,04819277	mg/kg bw
Dermal chronic		3,333333333	mg/kg bw/day		12,04819277	mg/kg bw/day
Oral acute	accidental			accidental		
Oral chronic	accidental			accidental		
Intake acute		3,760727754	mg/kg bw		12,94417625	mg/kg bw
Daily intake chronic		3,760727754	mg/kg bw/day		12,94417625	mg/kg bw/day

U = Undefined, D = Default, S = Set, O = Output, REM = Remark, not used in calculation

Scenario 10: cleaning/disinfection: disinfection wipe (small surfaces)

Results / Output

Mixing & Loading	Task A: not applicable						

Application	Task B: disinfection: wiping of surface						
	Primary exposure	User		Secondary exposure		Bystander	
**Inhalation**							
Concentration of potential exposure	205,1493218	mg/m^3	S		205,1493218	mg/m^3	S
Inhalation	2,849296137	mg/min	O		0,82629588	mg/min	O
Absorption per task	4,273944205	mg/task	O		1,239443819	mg/task	O
Exposure per task / Acute Dose	0,071232403	mg/kg bw	O		0,149330581	mg/kg bw	O
Exposure per day / Chronic Dose	0,071232403	mg/kg bw/day	O		0,149330581	mg/kg bw/day	O
**Dermal**							
Contact area between product and skin	0,042	m^2	S				
Quantity of contact (mg)	100	mg	S				
Dermal load (mg/cm^2)	0,238095238	mg/cm^2	O				
Absorption (mg)	100	mg	O				
Exposure per task / Acute Dose	1,666666667	mg/kg bw	O	no			S
Exposure per day / Chronic Dose	1,666666667	mg/kg bw/day	O	no			S
**Oral**							
Exposure / Dose	accidental		S	accidental			S
**Intake**							
Exposure per task / Acute Dose	1,73789907	mg/kg bw	O		0,149330581	mg/kg bw	O
Exposure per day / Chronic Dose	1,73789907	mg/kg bw/day	O		0,149330581	mg/kg bw/day	O

Post application phase	Task C: residence time						
	Primary exposure	User		Secondary exposure		Bystander	
**Inhalation**							
Concentration of potential exposure	205,1493218	mg/m^3	S		205,1493218	mg/m^3	S
Inhalation	2,849296137	mg/min	O		0,82629588	mg/min	O
Absorption per task	21,36972102	mg/task	O		6,197219097	mg/task	O
Exposure per task / Acute Dose	0,356162017	mg/kg bw	O		0,746652903	mg/kg bw	O
Exposure per day / Chronic Dose	0,356162017	mg/kg bw/day	O		0,746652903	mg/kg bw/day	O
**Dermal**							
Contact area between product and skin	0,042	m^2	S		0,01040125	m^2	S
Quantity of contact (mg)	100	mg	S		100	mg	S
Dermal load (mg/cm^2)	0,238095238	mg/cm^2	O		0,961422906	mg/cm^2	O
Absorption (mg)	100	mg	O		100	mg	O
Exposure per task / Acute Dose	1,666666667	mg/kg bw	O		12,04819277	mg/kg bw	O
Exposure per day / Chronic Dose	1,666666667	mg/kg bw/day	O		12,04819277	mg/kg bw/day	O
**Oral**							
Exposure / Dose	accidental		S	accidental			S
**Intake**							
Exposure per task / Acute Dose	2,022828684	mg/kg bw	O		12,79484567	mg/kg bw	O
Exposure per day / Chronic Dose	2,022828684	mg/kg bw/day	O		12,79484567	mg/kg bw/day	O

Disposal	Task D: not applicable						

U = Undefined, D = Default, S = Set, O = Output, REM = Remark, not used in calculation

Scenario 11: cleaning/disinfection: toilet cleaners (liquid/gel)

Scenario description

				Comments
Name of product	disinfection cleaner used for toilet cleaning		S	
Physical state product (liquid/solid)	liquid		S	
Density product	1	g/cm^3	D	
Concentration of active substance in product	1	%	S	*Frame Formulation*
Concentration of active substance in product (mg/l)	10000	mg/l	O	
User	Consumer		S	
Bystander	Children		S	
Temperature	20	°C	D	*room temperature*

Mixing & Loading

Task A: not applicable

Application

Task B: splashing cleaner into toilet bowl

Number of tasks per year	104	tasks/yr	S	*twice a week, max [AISE (2002) cited in TGD (2003)]*
Duration of task	1	min/task	S	*< 1 min [AISE (2002) cited in TGD (2003)]*
Quantity of product used per task	35	g/task	S	*max gel [AISE (2002) cited in TGD (2003)]*
Quantity of active substance used per task	350	mg/task	O	
Volume of product used per task	35	ml/task	O	
Model inhalation exposure	Exposure to vapour / Fugacity concept (equilibrium between product/solution and air, limited to vapour pressure)			
Room volume	1	m^3	S	*cloud around user*
REM Surface (treated per task), release area	*0,225*	*m^2/task*	*S*	*toilet bowl*
Model dermal exposure	Direct dermal contact			
Contact area between product and skin	0,0168	m^2	S	*Fingertips*
Model oral exposure	accidental			

Post application phase

Task C: residence time until flushing

Number of tasks per year	104	tasks/yr	S	*twice a week, max [AISE (2002) cited in TGD (2003)]*
Duration of task	30	min/task	S	
Quantity of product used per task	35	g/task	S	*max gel [AISE (2002) cited in TGD (2003)]*
Quantity of active substance used per task	350	mg/task	O	
Volume of product used per task	35	ml/task	O	
Model inhalation exposure	Exposure to vapour / Fugacity concept (equilibrium between product/solution and air, limited to vapour pressure)			
Room volume	2,5	m^3	S	*toilet*
REM Surface (treated per task), release area	*0,225*	*m^2/task*	*S*	*toilet bowl*
Model dermal exposure	Direct dermal contact			
Contact area between product and skin	0	m^2	S	*no direct contact*
Contact area between product and skin children	0	m^2	S	*no direct contact*
Model oral exposure	accidental			

Disposal

Task D: not applicable

Summary Results Exposure

	User	Consumer	Bystander	Children
Highest potential exposure acute (all amount used is absorbed)	5,833333333	mg/kg bw	42,1686747	mg/kg bw
Highest potential exposure chronic (all amount used is absorbed)	1,662100457	mg/kg bw/day	12,01518402	mg/kg bw/day
Highest potential concentration in air	2,039538648	mg/m^3	2,039538648	mg/m^3
Inhalation acute	0,010884637	mg/kg bw	0,022818397	mg/kg bw
Inhalation chronic	0,003101376	mg/kg bw/day	0,00650168	mg/kg bw/day
Dermal acute	0,28	mg/kg bw	0	mg/kg bw
Dermal chronic	0,079780822	mg/kg bw/day	0	mg/kg bw/day
Oral acute	accidental		accidental	
Oral chronic	accidental		accidental	
Intake acute	0,290884637	mg/kg bw	0,022818397	mg/kg bw
Daily intake chronic	0,082882198	mg/kg bw/day	0,00650168	mg/kg bw/day

U = Undefined, D = Default, S = Set, O = Output, REM = Remark, not used in calculation

Scenario 11: cleaning/disinfection: toilet cleaners (liquid/gel)

Results / Output

Mixing & Loading	Task A: not applicable						

Application	Task B: splashing cleaner into toilet bowl						
	Primary exposure	User		Secondary exposure		Bystander	
**Inhalation**							
Concentration of potential exposure	2,039538648	mg/m^3	O		2,039538648	mg/m^3	O
Inhalation	0,028326926	mg/min	O		0,008214808	mg/min	O
Absorption per task	0,021245194	mg/task	O		0,006161106	mg/task	O
Exposure per task / Acute Dose	0,000354087	mg/kg bw	O		0,000742302	mg/kg bw	O
Exposure per day / Chronic Dose	0,00010089	mg/kg bw/day	O		0,000211505	mg/kg bw/day	O
**Dermal**							
Contact area between product and skin	0,0168	m^2	S				
Volume of contact	1,68	ml	O				
Quantity of contact (mg)	16,8	mg	O				
Dermal load (mg/cm^2)	0,1	mg/cm^2	O				
Absorption (mg)	16,8	mg	O				
Exposure per task / Acute Dose	0,28	mg/kg bw	O	no			S
Exposure per day / Chronic Dose	0,079780822	mg/kg bw/day	O	no			S
**Oral**							
Exposure / Dose	accidental		S	accidental			S
**Intake**							
Exposure per task / Acute Dose	0,280354087	mg/kg bw	O		0,000742302	mg/kg bw	O
Exposure per day / Chronic Dose	0,079881712	mg/kg bw/day	O		0,000211505	mg/kg bw/day	O

Post application phase	Task C: residence time until flushing						
	Primary exposure	User		Secondary exposure		Bystander	
**Inhalation**							
Concentration of potential exposure	2,021865762	mg/m^3	O		2,021865762	mg/m^3	O
Inhalation	0,028081469	mg/min	O		0,008143626	mg/min	O
Absorption per task	0,631833051	mg/task	O		0,183231585	mg/task	O
Exposure per task / Acute Dose	0,010530551	mg/kg bw	O		0,022076095	mg/kg bw	O
Exposure per day / Chronic Dose	0,003000486	mg/kg bw/day	O		0,006290175	mg/kg bw/day	O
**Dermal**							
Exposure per task / Acute Dose	no	mg/kg bw	S	no		mg/kg bw	S
Exposure per day / Chronic Dose	no	mg/kg bw/day	S	no		mg/kg bw/day	S
**Oral**							
Exposure / Dose	accidental		S	accidental			S
**Intake**							
Exposure per task / Acute Dose	0,010530551	mg/kg bw	O		0,022076095	mg/kg bw	O
Exposure per day / Chronic Dose	0,003000486	mg/kg bw/day	O		0,006290175	mg/kg bw/day	O

Disposal	Task D: not applicable						

Scenario 12: cleaning/disinfection: drain cleaner (liquid)

Scenario description

				Comments
Name of product	disinfection cleaner used for drain cleaning		S	
Physical state product (liquid/solid)	liquid		S	
Density product	1	g/cm^3	D	
Concentration of active substance in product	1	%	S	*Frame Formulation*
Concentration of active substance in product (mg/l)	10000	mg/l	O	
User	Consumer		S	
Bystander	Children		S	
Temperature	20	°C	D	*room temperature*

Mixing & Loading

Task A: not applicable

Application

Task B: pouring the cleaner into the drain

Number of tasks per year	4	tasks/yr	S	*every three month*
Duration of task	1	min/task	S	
Quantity of product used per task	100	ml/task	S	
Quantity of active substance used per task	1000	mg/task	O	
Model inhalation exposure	Exposure to vapour / Fugacity concept (equilibrium between product/solution and air, limited to vapour pressure)			
Room volume	1	m^3	S	*cloud around user*
REM Surface (treated per task), release area	*0,00159*	*m^2/task*	*S*	*drain of bathtub or shower*
Model dermal exposure	Direct dermal contact			
Contact area between product and skin	0,0168	m^2	S	*Fingertips*
Model oral exposure	accidental			

Post application phase

Task C: residence time + flushing

Number of tasks per year	4	tasks/yr	S	*every three month*
Duration of task	30	min/task	S	*Bref 111 Tips*
Quantity of product used per task	100	ml/task	S	
Quantity of active substance used per task	1000	mg/task	O	
Model inhalation exposure	Exposure to vapour / Fugacity concept (equilibrium between product/solution and air, limited to vapour pressure)			
Room volume	10	m^3	S	*bathroom*
REM Surface (treated per task), release area	*0,00159*	*m^2/task*	*S*	*drain of bathtub or shower*
Model dermal exposure	Direct dermal contact			
Contact area between product and skin	0	m^2	S	*no direct contact*
Contact area between product and skin children	0	m^2	S	*no direct contact*
Model oral exposure	accidental			

Disposal

Task D: not applicable

Summary Results Exposure

	User	Consumer		Bystander	Children	
Highest potential exposure acute (all amount used is absorbed)		16,66666667	mg/kg bw		120,4819277	mg/kg bw
Highest potential exposure chronic (all amount used is absorbed)		0,182648402	mg/kg bw/day		1,320349893	mg/kg bw/day
Highest potential concentration in air		2,04729321	mg/m^3		2,04729321	mg/m^3
Inhalation acute		0,010825501	mg/kg bw		0,022694423	mg/kg bw
Inhalation chronic		0,000118636	mg/kg bw/day		0,000248706	mg/kg bw/day
Dermal acute		0,28	mg/kg bw		0	mg/kg bw
Dermal chronic		0,003068493	mg/kg bw/day		0	mg/kg bw/day
Oral acute	accidental			accidental		
Oral chronic	accidental			accidental		
Intake acute		0,290825501	mg/kg bw		0,022694423	mg/kg bw
Daily intake chronic		0,003187129	mg/kg bw/day		0,000248706	mg/kg bw/day

U = Undefined, D = Default, S = Set, O = Output, REM = Remark, not used in calculation

Scenario 12: cleaning/disinfection: drain cleaner (liquid)

Results / Output

Mixing & Loading	Task A: not applicable					

Application	Task B: pouring the cleaner into the drain					
	Primary exposure	User		Secondary exposure	Bystander	
**Inhalation**						
Concentration of potential exposure	2,04729321	mg/m^3	O	2,04729321	mg/m^3	O
Inhalation	0,028434628	mg/min	O	0,008246042	mg/min	O
Absorption per task	0,021325971	mg/task	O	0,006184532	mg/task	O
Exposure per task / Acute Dose	0,000355433	mg/kg bw	O	0,000745124	mg/kg bw	O
Exposure per day / Chronic Dose	3,89515E-06	mg/kg bw/day	O	8,16575E-06	mg/kg bw/day	O
**Dermal**						
Contact area between product and skin	0,0168	m^2	S			
Volume of contact	1,68	ml	O			
Quantity of contact (mg)	16,8	mg	O			
Dermal load (mg/cm^2)	0,1	mg/cm^2	O			
Absorption (mg)	16,8	mg	O			
Exposure per task / Acute Dose	0,28	mg/kg bw	O	no		S
Exposure per day / Chronic Dose	0,003068493	mg/kg bw/day	O	no		S
**Oral**						
Exposure / Dose	accidental		S	accidental		S
**Intake**						
Exposure per task / Acute Dose	0,280355433	mg/kg bw	O	0,000745124	mg/kg bw	O
Exposure per day / Chronic Dose	0,003072388	mg/kg bw/day	O	8,16575E-06	mg/kg bw/day	O

Post application phase	Task C: residence time + flushing					
	Primary exposure	User		Secondary exposure	Bystander	
**Inhalation**						
Concentration of potential exposure	2,010253014	mg/m^3	O	2,010253014	mg/m^3	O
Inhalation	0,027920181	mg/min	O	0,008096852	mg/min	O
Absorption per task	0,628204067	mg/task	O	0,182179179	mg/task	O
Exposure per task / Acute Dose	0,010470068	mg/kg bw	O	0,021949299	mg/kg bw	O
Exposure per day / Chronic Dose	0,00011474	mg/kg bw/day	O	0,00024054	mg/kg bw/day	O
**Dermal**						
Exposure per task / Acute Dose	no	mg/kg bw	S	no	mg/kg bw	S
Exposure per day / Chronic Dose	no	mg/kg bw/day	S	no	mg/kg bw/day	S
**Oral**						
Exposure / Dose	accidental		S	accidental		S
**Intake**						
Exposure per task / Acute Dose	0,010470068	mg/kg bw	O	0,021949299	mg/kg bw	O
Exposure per day / Chronic Dose	0,00011474	mg/kg bw/day	O	0,00024054	mg/kg bw/day	O

Disposal	Task D: not applicable					

U = Undefined, D = Default, S = Set, O = Output, REM = Remark, not used in calculation

Scenario 13: cleaning/disinfection: swimming pool water treatment

Scenario description

				Comments
Name of product	disinfection cleaner		S	
Physical state product (liquid/solid)	liquid		S	
Density product	1	g/cm^3	D	
Concentration of active substance in product	1	%	S	*Frame Formulation*
Concentration of active substance in product (mg/l)	10000	mg/l	O	
User	Consumer		S	
Bystander	Children		S	
Temperature	20	°C	D	*room temperature*
**Mixing & Loading**	Task A: simple dilution with water in skimmer			
Number of tasks per year	52	tasks/yr	S	*once a week*
Duration of task	1	min/task	S	
Quantity of product used per task	100	ml/task	S	
Quantity of water used per task	2	L/task	S	*water/volume of skimmer*
Quantity of active substance used per task	1000	mg/task	O	
Concentration of active substance in skimmer	500	mg/l	O	
Model inhalation exposure	Exposure to vapour / Fugacity concept (equilibrium between skimmer solution and air, limited to vapour pressure)			
Room volume	1	m^3	S	
REM Evaporation from mixture, release area concentrate	*0,002*	m^2	*S*	*bottle diameter 5 cm [TNsG 2002 p. 252]*
REM Evaporation from mixture, release area skimmer	*???*	m^2	*U*	*surface of skimmer*
Model dermal exposure	Direct dermal contact			
Contact area between product (concentrate) and skin	0,0168	m^2	S	*Fingertips*
Model oral exposure	accidental			
**Application**	Task B: not applicable			
**Post application phase**	Task C: residence time, swimming			
Number of tasks per year	365	tasks/yr	S	*once a day*
Duration of task	15	min/task	S	
Quantity of water in swimming pool	90	m^3	S	*water of swimming pool (6 x 10 x 1.5 m)*
Quantity of active substance used per task	1000	mg/task	O	
Concentration of active substance in swimming pool water	0,011111111	mg/l	O	
Model inhalation exposure	Exposure to vapour / Fugacity concept (equilibrium between swimming pool water and air, limited to vapour pressure)			
REM Evaporation from mixture, release area swimming pool	*60*	m^2	*S*	*6 x 10 m*
Room volume (above water surface)	60	m^3	S	*6 x 10 x 1 m*
Model dermal exposure	Direct dermal contact			
Contact area between swimming pool water and skin	1,94	m^2	S	*Whole body*
Contact area between swimming pool water and skin children	0,3925	m^2	S	*Whole body Children*
Model oral exposure	Direct oral ingestion			
Volume of swimming pool water swallowed	20	ml	S	
**Disposal**	Task D: not applicable			

Summary Results Exposure

	User	Consumer		Bystander	Children
Highest potential exposure acute (all amount used is absorbed)	16,66666667	mg/kg bw		120,4819277	mg/kg bw
Highest potential exposure chronic (all amount used is absorbed)	2,374429224	mg/kg bw/day		17,16454861	mg/kg bw/day
Highest potential concentration in air	0,10256414	mg/m^3		0,10256414	mg/m^3
Inhalation acute	1,78122E-05	mg/kg bw		3,73413E-05	mg/kg bw
Inhalation chronic	2,54272E-06	mg/kg bw/day		5,33052E-06	mg/kg bw/day
Dermal acute	0,280035926	mg/kg bw		5,25435E-05	mg/kg bw
Dermal chronic	0,039926337	mg/kg bw/day		5,25435E-05	mg/kg bw/day
Oral acute	3,7037E-06	mg/kg bw		2,67738E-05	mg/kg bw
Oral chronic	3,7037E-06	mg/kg bw/day		2,67738E-05	mg/kg bw/day
Intake acute	0,280057442	mg/kg bw		0,000116659	mg/kg bw
Daily intake chronic	0,039932583	mg/kg bw/day		8,46478E-05	mg/kg bw/day

U = Undefined, D = Default, S = Set, O = Output, REM = Remark, not used in calculation

Scenario 13: cleaning/disinfection: swimming pool water treatment

Results / Output

**Mixing & Loading**	Task A: simple dilution with water in skimmer					
	**Primary exposure**	**User**		**Secondary exposure**	**Bystander**	
**Inhalation**						
Concentration of potential exposure	0,10256414	mg/m^3	O	0,10256414	mg/m^3	O
Inhalation	0,001424502	mg/min	O	0,000413106	mg/min	O
Absorption per task	0,001068376	mg/task	O	0,000309829	mg/task	O
Exposure per task / Acute Dose	1,78063E-05	mg/kg bw	O	3,73288E-05	mg/kg bw	O
Exposure per day / Chronic Dose	2,53678E-06	mg/kg bw/day	O	5,31808E-06	mg/kg bw/day	O
**Dermal**						
Contact area between product (concentrate) and skin	0,0168	m^2	S			
Volume of contact	1,68	ml	O			
Quantity of contact (mg)	16,8	mg	O			
Dermal load (mg/cm^2)	0,1	mg/cm^2	O			
Absorption (mg)	16,8	mg	O			
Exposure per task / Acute Dose	0,28	mg/kg bw	O	no		S
Exposure per day / Chronic Dose	0,039890411	mg/kg bw/day	O	no		S
**Oral**						
Exposure / Dose	accidental		S	accidental		S
**Intake**						
Exposure per task / Acute Dose	0,280017806	mg/kg bw	O	3,73288E-05	mg/kg bw	O
Exposure per day / Chronic Dose	0,039892948	mg/kg bw/day	O	5,31808E-06	mg/kg bw/day	O

**Application**	Task B: not applicable

**Post application phase**	Task C: residence time, swimming					
	**Primary exposure**	**User**		**Secondary exposure**	**Bystander**	
**Inhalation**						
Concentration of potential exposure	2,27944E-06	mg/m^3	O	2,27944E-06	mg/m^3	O
Inhalation	3,16588E-08	mg/min	O	9,18106E-09	mg/min	O
Absorption per task	3,56162E-07	mg/task	O	1,03287E-07	mg/task	O
Exposure per task / Acute Dose	5,93603E-09	mg/kg bw	O	1,24442E-08	mg/kg bw	O
Exposure per day / Chronic Dose	5,93603E-09	mg/kg bw/day	O	1,24442E-08	mg/kg bw/day	O
**Dermal**						
Contact area between swimming pool water and skin	1,94	m^2	S	0,3925	m^2	S
Volume of contact	194	ml	O	39,25	ml	O
Quantity of contact (mg)	0,002155556	mg	O	0,000436111	mg	O
Dermal load (mg/cm^2)	1,11111E-07	mg/cm^2	O	1,11111E-07	mg/cm^2	O
Absorption (mg)	0,002155556	mg	O	0,000436111	mg	O
Exposure per task / Acute Dose	3,59259E-05	mg/kg bw	O	5,25435E-05	mg/kg bw	O
Exposure per day / Chronic Dose	3,59259E-05	mg/kg bw/day	O	5,25435E-05	mg/kg bw/day	O
**Oral**						
Absorption per task	0,000222222	mg/task	O	0,000222222	mg/task	O
Exposure per task / Acute Dose	3,7037E-06	mg/kg bw	O	2,67738E-05	mg/kg bw	O
Exposure per day / Chronic Dose	3,7037E-06	mg/kg bw/day	O	2,67738E-05	mg/kg bw/day	O
**Intake**						
Exposure per task / Acute Dose	3,96356E-05	mg/kg bw	O	7,93297E-05	mg/kg bw	O
Exposure per day / Chronic Dose	3,96356E-05	mg/kg bw/day	O	7,93297E-05	mg/kg bw/day	O

**Disposal**	Task D: not applicable

U = Undefined, D = Default, S = Set, O = Output, REM = Remark, not used in calculation

Scenario 14: cleaning/disinfection: textiles, machine wash using laundry compact (liquid/gel)

Scenario description

				Comments
Name of product	laundry compact liquid		S	
Physical state product (liquid/solid)	liquid		S	
Density product	1	g/cm^3	D	
Concentration of active substance in product	1	%	S	*Frame Formulation*
Concentration of active substance in product (mg/l)	10000	mg/l	O	
User	Consumer		S	
Bystander	Children		S	
Temperature	20	°C	D	*room temperature*
**Mixing & Loading**	Task A: fill in			
Number of tasks per year	520	tasks/yr	S	*ten times a week, max [AISE (2002) cited in TGD (2003)]*
Duration of task	1	min/task	S	*< 1 min [AISE (2002) cited in TGD (2003)]*
Quantity of product used per task	140	g/task	S	*max [AISE (2002) cited in TGD (2003)]*
Quantity of active substance used per task	1400	mg/task	O	
Volume of product used per task	140	ml/task	O	
Model inhalation exposure	Exposure to vapour / Fugacity concept (equilibrium between product/solution and air, limited to vapour pressure)			
Room volume	1	m^3	S	*cloud around user*
REM Evaporation from mixture, release area	*0,002*	*m^2*	*S*	*bottle diameter 5 cm [TNsG 2002 p. 252]*
Model dermal exposure	Direct dermal contact			
Contact area between product (concentrate) and skin	0,0168	m^2	S	*Fingertips*
Model oral exposure	accidental			
**Application**	Task B: no inhalative or dermal contact during washing procedure -> not applicable			
**Post application phase**	Task C: wearing of clothes			
Number of tasks per year	365	tasks/yr	S	*once a day*
Duration of task	720	min/task	S	*12 hours*
Weight fraction of product deposited on fabric	5	%	S	*HERA (worst case assumption)*
Fabric density	20	mg/cm^2	S	*HERA (given for all cotton)*
Total weight of fabric	1	kg	S	*HERA (Estimation)*
Model inhalation exposure	Exposure to vapour / Instantaneous release (limited to vapour pressure)			
Room volume	1	m^3	S	*cloud around user*
Model dermal exposure	Indirect dermal contact			
Contact area between textile and skin	1,94	m^2	S	*Whole body, HERA: 1.76 m^2*
Contact area between textile and skin children	0,3925	m^2	S	*Whole body Children*
Weight fraction transferred from textile to skin, migration factor	1	%	S	*HERA (Vermeire et al. 1993)*
Model oral exposure	accidental			
**Disposal**	Task D: not applicable			

Summary Results Exposure

	User	Consumer		Bystander	Children	
Highest potential exposure acute (all amount used is absorbed)		23,33333333	mg/kg bw		168,6746988	mg/kg bw
Highest potential exposure chronic (all amount used is absorbed)		33,24200913	mg/kg bw/day		240,3036805	mg/kg bw/day
Highest potential concentration in air		70	mg/m^3		70	mg/m^3
Inhalation acute		1,167022308	mg/kg bw		8,4344805	mg/kg bw
Inhalation chronic		1,167173333	mg/kg bw/day		8,434797108	mg/kg bw/day
Dermal acute		0,284526667	mg/kg bw		0,006620482	mg/kg bw
Dermal chronic		0,403430776	mg/kg bw/day		0,006620482	mg/kg bw/day
Oral acute	accidental			accidental		
Oral chronic	accidental			accidental		
Intake acute		1,447022308	mg/kg bw		8,4344805	mg/kg bw
Daily intake chronic		1,566077443	mg/kg bw/day		8,434797108	mg/kg bw/day

U = Undefined, D = Default, S = Set, O = Output, REM = Remark, not used in calculation

Scenario 14: cleaning/disinfection: textiles, machine wash using laundry compact (liquid/gel)

Results / Output

Mixing & Loading	Task A: fill in					
	**Primary exposure**	**User**		**Secondary exposure**	**Bystander**	
**Inhalation**						
Concentration of potential exposure	2,048491457	mg/m^3	O	2,048491457	mg/m^3	O
Inhalation	0,02845127	mg/min	O	0,008250868	mg/min	O
Absorption per task	0,021338453	mg/task	O	0,006188151	mg/task	O
Exposure per task / Acute Dose	0,000355641	mg/kg bw	O	0,00074556	mg/kg bw	O
Exposure per day / Chronic Dose	0,000506666	mg/kg bw/day	O	0,001062168	mg/kg bw/day	O
**Dermal**						
Contact area between product (concentrate) and skin	0,0168	m^2	S			
Volume of contact	1,68	ml	O			
Quantity of contact (mg)	16,8	mg	O			
Dermal load (mg/cm^2)	0,1	mg/cm^2	O			
Absorption (mg)	16,8	mg	O			
Exposure per task / Acute Dose	0,28	mg/kg bw	O	no		S
Exposure per day / Chronic Dose	0,39890411	mg/kg bw/day	O	no		S
**Oral**						
Exposure / Dose	accidental		S	accidental		S
**Intake**						
Exposure per task / Acute Dose	0,280355641	mg/kg bw	O	0,00074556	mg/kg bw	O
Exposure per day / Chronic Dose	0,399410776	mg/kg bw/day	O	0,001062168	mg/kg bw/day	O

Application	Task B: no inhalative or dermal contact during washing procedure -> not applicable

Post application phase	Task C: wearing of clothes					
	**Primary exposure**	**User**		**Secondary exposure**	**Bystander**	
**Inhalation**						
Concentration of potential exposure	70	mg/m^3	O	70	mg/m^3	O
Inhalation	0,972222222	mg/min	O	0,281944444	mg/min	O
Absorption per task	70	mg/task	S	70	mg/task	S
Exposure per task / Acute Dose	1,166666667	mg/kg bw	O	8,43373494	mg/kg bw	O
Exposure per day / Chronic Dose	1,166666667	mg/kg bw/day	O	8,43373494	mg/kg bw/day	O
**Dermal**						
Contact area between textile and skin	1,94	m^2	S	0,3925	m^2	S
Dermal load (mg/cm^2)	0,0014	mg/cm^2	O	0,0014	mg/cm^2	O
Absorption (mg)	0,2716	mg	O	0,05495	mg	O
Exposure per task / Acute Dose	0,004526667	mg/kg bw	O	0,006620482	mg/kg bw	O
Exposure per day / Chronic Dose	0,004526667	mg/kg bw/day	O	0,006620482	mg/kg bw/day	O
**Oral**						
Exposure / Dose	accidental		S	accidental		S
**Intake**						
Exposure per task / Acute Dose	1,166666667	mg/kg bw	S	8,43373494	mg/kg bw	S
Exposure per day / Chronic Dose	1,166666667	mg/kg bw/day	O	8,43373494	mg/kg bw/day	O

Disposal	Task D: not applicable

U = Undefined, D = Default, S = Set, O = Output, REM = Remark, not used in calculation

Scenario 14b: cleaning/disinfection: textiles, machine wash using laundry additives (liquid)

Scenario description

				Comments
Name of product	hygiene laundry rinse		S	
Physical state product (liquid/solid)	liquid		S	
Density product	1	g/cm^3	D	
Concentration of active substance in product	1	%	S	*Frame Formulation*
Concentration of active substance in product (mg/l)	10000	mg/l	O	
User	Consumer		S	
Bystander	Children		S	
Temperature	20	°C	D	*room temperature*
**Mixing & Loading**	Task A: fill in			
Number of tasks per year	208	tasks/yr	S	*four times a week, max [AISE (2002) cited in TGD (2003)]*
Duration of task	1	min/task	S	*< 1 min [AISE (2002) cited in TGD (2003)]*
Quantity of product used per task	100	g/task	S	*max [AISE (2002) cited in TGD (2003)]*
Quantity of active substance used per task	1000	mg/task	O	
Volume of product used per task	100	ml/task	O	
Model inhalation exposure	Exposure to vapour / Fugacity concept (equilibrium between product/solution and air, limited to vapour pressure)			
Room volume	1	m^3	S	*cloud around user*
REM Evaporation from mixture, release area	*0,002*	*m^2*	*S*	*bottle diameter 5 cm [TNsG 2002 p. 252]*
Model dermal exposure	Direct dermal contact			
Contact area between product (concentrate) and skin	0,0168	m^2	S	*Fingertips*
Model oral exposure	accidental			
**Application**	Task B: no inhalative or dermal contact during washing procedure -> not applicable			
**Post application phase**	Task C: wearing of clothes			
Number of tasks per year	208	tasks/yr	S	*four times a week*
Duration of task	720	min/task	S	*12 hours*
Weight fraction of product deposited on fabric	5	%	S	*HERA (worst case assumption)*
Fabric density	20	mg/cm^2	S	*HERA (given for all cotton)*
Total weight of fabric	1	kg	S	*HERA (Estimation)*
Model inhalation exposure	Exposure to vapour / Instantaneous release (limited to vapour pressure)			
Room volume	1	m^3	S	*cloud around user*
Model dermal exposure	Indirect dermal contact			
Contact area between textile and skin	1,94	m^2	S	*Whole body, HERA: 1.76 m^2*
Contact area between textile and skin children	0,3925	m^2	S	*Whole body Children*
Weight fraction transferred from textile to skin, migration factor	1	%	S	*HERA (Vermeire et al. 1993)*
Model oral exposure	accidental			
**Disposal**	Task D: not applicable			

Summary Results Exposure

	**User**	**Consumer**		**Bystander**	**Children**	
Highest potential exposure acute (all amount used is absorbed)		16,66666667	mg/kg bw		120,4819277	mg/kg bw
Highest potential exposure chronic (all amount used is absorbed)		9,497716895	mg/kg bw/day		68,65819442	mg/kg bw/day
Highest potential concentration in air		50	mg/m^3		50	mg/m^3
Inhalation acute		0,833688766	mg/kg bw		6,02484151	mg/kg bw
Inhalation chronic		0,475088393	mg/kg bw/day		3,43333434	mg/kg bw/day
Dermal acute		0,283233333	mg/kg bw		0,004728916	mg/kg bw
Dermal chronic		0,161404201	mg/kg bw/day		0,002694834	mg/kg bw/day
Oral acute	accidental			accidental		
Oral chronic	accidental			accidental		
Intake acute		1,113688766	mg/kg bw		6,02484151	mg/kg bw
Daily intake chronic		0,634650037	mg/kg bw/day		3,43333434	mg/kg bw/day

U = Undefined, D = Default, S = Set, O = Output, REM = Remark, not used in calculation

Scenario 14b: cleaning/disinfection: textiles, machine wash using laundry additives (liquid)

Results / Output

Mixing & Loading	Task A: fill in					
	**Primary exposure**	**User**		**Secondary exposure**	**Bystander**	
**Inhalation**						
Concentration of potential exposure	2,04729321	mg/m^3	O	2,04729321	mg/m^3	O
Inhalation	0,028434628	mg/min	O	0,008246042	mg/min	O
Absorption per task	0,021325971	mg/task	O	0,006184532	mg/task	O
Exposure per task / Acute Dose	0,000355433	mg/kg bw	O	0,000745124	mg/kg bw	O
Exposure per day / Chronic Dose	0,000202548	mg/kg bw/day	O	0,000424619	mg/kg bw/day	O
**Dermal**						
Contact area between product (concentrate) and skin	0,0168	m^2	S			
Volume of contact	1,68	ml	O			
Quantity of contact (mg)	16,8	mg	O			
Dermal load (mg/cm^2)	0,1	mg/cm^2	O			
Absorption (mg)	16,8	mg	O			
Exposure per task / Acute Dose	0,28	mg/kg bw	O	no		S
Exposure per day / Chronic Dose	0,159561644	mg/kg bw/day	O	no		S
**Oral**						
Exposure / Dose	accidental		S	accidental		S
**Intake**						
Exposure per task / Acute Dose	0,280355433	mg/kg bw	O	0,000745124	mg/kg bw	O
Exposure per day / Chronic Dose	0,159764192	mg/kg bw/day	O	0,000424619	mg/kg bw/day	O

Application	Task B: no inhalative or dermal contact during washing procedure -> not applicable

Post application phase	Task C: wearing of clothes					
	**Primary exposure**	**User**		**Secondary exposure**	**Bystander**	
**Inhalation**						
Concentration of potential exposure	50	mg/m^3	O	50	mg/m^3	O
Inhalation	0,694444444	mg/min	O	0,201388889	mg/min	O
Absorption per task	50	mg/task	S	50	mg/task	S
Exposure per task / Acute Dose	0,833333333	mg/kg bw	O	6,024096386	mg/kg bw	O
Exposure per day / Chronic Dose	0,474885845	mg/kg bw/day	O	3,432909721	mg/kg bw/day	O
**Dermal**						
Contact area between textile and skin	1,94	m^2	S	0,3925	m^2	S
Dermal load (mg/cm^2)	0,001	mg/cm^2	O	0,001	mg/cm^2	O
Absorption (mg)	0,194	mg	O	0,03925	mg	O
Exposure per task / Acute Dose	0,003233333	mg/kg bw	O	0,004728916	mg/kg bw	O
Exposure per day / Chronic Dose	0,001842557	mg/kg bw/day	O	0,002694834	mg/kg bw/day	O
**Oral**						
Exposure / Dose	accidental		S	accidental		S
**Intake**						
Exposure per task / Acute Dose	0,833333333	mg/kg bw	S	6,024096386	mg/kg bw	S
Exposure per day / Chronic Dose	0,474885845	mg/kg bw/day	O	3,432909721	mg/kg bw/day	O

Disposal	Task D: not applicable

U = Undefined, D = Default, S = Set, O = Output, REM = Remark, not used in calculation

Scenario 15: cleaning/disinfection: textiles, hand wash using laundry compact (liquid/gel)

Scenario description

			Comments
Name of product	laundry compact liquid	S	
Physical state product (liquid/solid)	liquid	S	
Density product	1 g/cm^3	D	
Concentration of active substance in product	1 %	S	*Frame Formulation*
Concentration of active substance in product (mg/l)	10000 mg/l	O	
User	Consumer	S	
Bystander	Children	S	
Temperature	20 °C	D	*room temperature*

Mixing & Loading

	Task A: simple dilution with water in vessel		
Number of tasks per year	520 tasks/yr	S	*ten times a week, max [AISE (2002) cited in TGD (2003)]*
Duration of task	1 min/task	S	
Quantity of water used per task	20 L/task	S	*50% of sink volume (40x40x25 cm)*
Quantity of product used per task	200 g/task	S	*0.1 - 1% [AISE (2002) cited in TGD (2003)]*
Quantity of active substance used per task	2000 mg/task	O	
Concentration of active substance in washing solution	100 mg/l	O	
Model inhalation exposure	Exposure to vapour / Fugacity concept (equilibrium between washing solution and air, limited to vapour pressure)		
Room volume	1 m^3	S	*cloud around user*
REM Evaporation from mixture, release area concentrate	*0,002 m^2*	*S*	*bottle diameter 5 cm [TNsG 2002 p. 252]*
REM Evaporation from mixture, release area washing solution	*0,16 m^2*	*S*	*surface vessel (40 x 40 cm)*
Model dermal exposure	Direct dermal contact		
Contact area between product (concentrate) and skin	0,0168 m^2	S	*Fingertips*
Model oral exposure	accidental		

Application

	Task B: washing the textiles		
Number of tasks per year	520 tasks/yr	S	*ten times a week, max [AISE (2002) cited in TGD (2003)]*
Duration of task	10 min/task	S	*[AISE (2002) cited in TGD (2003)]*
Quantity of water used per task	20 L/task	S	*50% of sink volume (40x40x25 cm)*
Quantity of product used per task	200 g/task	S	*0.1 - 1% [AISE (2002) cited in TGD (2003)]*
Quantity of active substance used per task	2000 mg/task	O	
Concentration of active substance in washing solution	100 mg/l	O	
Model inhalation exposure	Exposure to vapour / Fugacity concept (equilibrium between washing solution and air, limited to vapour pressure)		
Room volume	10 m^3	S	*Bathroom*
REM Evaporation from mixture, release area washing solution	*0,16 m^2*	*S*	*surface vessel (40 x 40 cm)*
Model dermal exposure	Direct dermal contact		
Contact area between washing solution and skin	0,198 m^2	S	*Forearms, Hands*
Model oral exposure	accidental		

Post application phase

	Task C: wearing of clothes		
Number of tasks per year	365 tasks/yr	S	*once a day*
Duration of task	720 min/task	S	*12 hours*
Weight fraction of product deposited on fabric	5 %	S	*HERA (worst case assumption)*
Fabric density	20 mg/cm^2	S	*HERA (given for all cotton)*
Total weight of fabric	1 kg	S	*HERA (Estimation)*
Model inhalation exposure	Exposure to vapour / Instantaneous release (limited to vapour pressure)		
Room volume	1 m^3	S	*cloud around user*
Model dermal exposure	Indirect dermal contact		
Contact area between textile and skin	1,94 m^2	S	*Whole body, HERA: 1.76 m^2*
Contact area between textile and skin children	0,3925 m^2	S	*Whole body Children*
Weight fraction transferred from textile to skin, migration factor	1 %	S	*HERA (Vermeire et al. 1993)*
Model oral exposure	accidental		

Disposal

	Task D: excessive washing solution is disposed to the main drainage		
Number of tasks per year	520 tasks/yr	S	*ten times a week, max [AISE (2002) cited in TGD (2003)]*
Duration of task	1 min/task	S	
Quantity of water disposed per task	20 L/task	S	*50% sink 40x40x25 cm*
Quantity of product disposed per task	190 g/task	S	*0.1 - 1% [AISE (2002) cited in TGD (2003)]*
Quantity of active substance disposed per task	1900 mg/task	S	
Concentration of active substance in washing solution	100 mg/l	O	
Model inhalation exposure	Exposure to vapour / Fugacity concept (equilibrium between washing solution and air, limited to vapour pressure)		
Room volume	1 m^3	S	
REM Evaporation from mixture, release area washing solution	*0,16 m^2*	*S*	*surface vessel (40 x 40 cm)*
Model dermal exposure	Direct dermal contact		
Contact area between washing solution and skin	0,198 m^2	S	*Hands, Forearms*
Model oral exposure	accidental		

Summary Results Exposure

	**User**	**Consumer**	**Bystander**	**Children**
Highest potential exposure acute (all amount used is absorbed)		33,33333333 mg/kg bw		240,9638554 mg/kg bw
Highest potential exposure chronic (all amount used is absorbed)		47,48858447 mg/kg bw/day		343,2909721 mg/kg bw/day
Highest potential concentration in air		100 mg/m^3		100 mg/m^3
Inhalation acute		1,666709224 mg/kg bw		12,04828199 mg/kg bw
Inhalation chronic		1,666727297 mg/kg bw/day		12,04831988 mg/kg bw/day
Dermal acute		0,352466667 mg/kg bw		0,009457831 mg/kg bw
Dermal chronic		0,499398174 mg/kg bw/day		0,009457831 mg/kg bw/day
Oral acute	accidental		accidental	
Oral chronic	accidental		accidental	
Intake acute		2,012709224 mg/kg bw		12,04828199 mg/kg bw
Daily intake chronic		2,159658804 mg/kg bw/day		12,04831988 mg/kg bw/day

U = Undefined, D = Default, S = Set, O = Output, REM = Remark, not used in calculation

Scenario 15: cleaning/disinfection: textiles, hand wash using laundry compact (liquid/gel)

Results / Output

Mixing & Loading	Task A: simple dilution with water in vessel					
	**Primary exposure**	**User**		**Secondary exposure**	**Bystander**	
**Inhalation**						
Concentration of potential exposure	0,020514722	mg/m^3	O	0,020514722	mg/m^3	O
Inhalation	0,000284927	mg/min	O	8,26287E-05	mg/min	O
Absorption per task	0,000213695	mg/task	O	6,19716E-05	mg/task	O
Exposure per task / Acute Dose	3,56158E-06	mg/kg bw	O	7,46645E-06	mg/kg bw	O
Exposure per day / Chronic Dose	5,07404E-06	mg/kg bw/day	O	1,06371E-05	mg/kg bw/day	O
**Dermal**						
Contact area between product (concentrate) and skin	0,0168	m^2	S			
Volume of contact	1,68	ml	O			
Quantity of contact (mg)	16,8	mg	O			
Dermal load (mg/cm^2)	0,1	mg/cm^2	O			
Absorption (mg)	16,8	mg	O			
Exposure per task / Acute Dose	0,28	mg/kg bw	O	no		S
Exposure per day / Chronic Dose	0,39890411	mg/kg bw/day	O	no		S
**Oral**						
Exposure / Dose	accidental		S	accidental		S
**Intake**						
Exposure per task / Acute Dose	0,280003562	mg/kg bw	O	7,46645E-06	mg/kg bw	O
Exposure per day / Chronic Dose	0,398909184	mg/kg bw/day	O	1,06371E-05	mg/kg bw/day	O

Application	Task B: washing the textiles					
	**Primary exposure**	**User**		**Secondary exposure**	**Bystander**	
**Inhalation**						
Concentration of potential exposure	0,020512828	mg/m^3	O	0,020512828	mg/m^3	O
Inhalation	0,0002849	mg/min	O	8,26211E-05	mg/min	O
Absorption per task	0,002136753	mg/task	O	0,000619658	mg/task	O
Exposure per task / Acute Dose	3,56125E-05	mg/kg bw	O	7,46576E-05	mg/kg bw	O
Exposure per day / Chronic Dose	5,07357E-05	mg/kg bw/day	O	0,000106362	mg/kg bw/day	O
**Dermal**						
Contact area between washing solution and skin	0,198	m^2	S			
Volume of contact	19,8	ml	O			
Quantity of contact (mg)	1,98	mg	O			
Dermal load (mg/cm^2)	0,001	mg/cm^2	O			
Absorption (mg)	1,98	mg	O			
Exposure per task / Acute Dose	0,033	mg/kg bw	O	no		S
Exposure per day / Chronic Dose	0,047013699	mg/kg bw/day	O	no		S
**Oral**						
Exposure / Dose	accidental		S	accidental		S
**Intake**						
Exposure per task / Acute Dose	0,033035613	mg/kg bw	O	7,46576E-05	mg/kg bw	O
Exposure per day / Chronic Dose	0,047064434	mg/kg bw/day	O	0,000106362	mg/kg bw/day	O

Post application phase	Task C: wearing of clothes					
	**Primary exposure**	**User**		**Secondary exposure**	**Bystander**	
**Inhalation**						
Concentration of potential exposure	100	mg/m^3	O	100	mg/m^3	O
Inhalation	1,388888889	mg/min	O	0,402777778	mg/min	O
Absorption per task	100	mg/task	S	100	mg/task	S
Exposure per task / Acute Dose	1,666666667	mg/kg bw	O	12,04819277	mg/kg bw	O
Exposure per day / Chronic Dose	1,666666667	mg/kg bw/day	O	12,04819277	mg/kg bw/day	O
**Dermal**						
Contact area between textile and skin	1,94	m^2	S	0,3925	m^2	S
Dermal load (mg/cm^2)	0,002	mg/cm^2	O	0,002	mg/cm^2	O
Absorption (mg)	0,388	mg	O	0,0785	mg	O
Exposure per task / Acute Dose	0,006466667	mg/kg bw	O	0,009457831	mg/kg bw	O
Exposure per day / Chronic Dose	0,006466667	mg/kg bw/day	O	0,009457831	mg/kg bw/day	O
**Oral**						
Exposure / Dose	accidental		S	accidental		S
**Intake**						
Exposure per task / Acute Dose	1,666666667	mg/kg bw	S	12,04819277	mg/kg bw	S
Exposure per day / Chronic Dose	1,666666667	mg/kg bw/day	O	12,04819277	mg/kg bw/day	O

Disposal	Task D: excessive washing solution is disposed to the main drainage					
	**Primary exposure**	**User**		**Secondary exposure**	**Bystander**	
**Inhalation**						
Concentration of potential exposure	0,019488986	mg/m^3	O	0,019488986	mg/m^3	O
Inhalation	0,00027068	mg/min	O	7,84973E-05	mg/min	O
Absorption per task	0,00020301	mg/task	O	5,8873E-05	mg/task	O
Exposure per task / Acute Dose	3,3835E-06	mg/kg bw	O	7,09313E-06	mg/kg bw	O
Exposure per day / Chronic Dose	4,82034E-06	mg/kg bw/day	O	1,01053E-05	mg/kg bw/day	O
**Dermal**						
Contact area between washing solution and skin	0,198	m^2	S			
Volume of contact	19,8	ml	O			
Quantity of contact (mg)	1,98	mg	O			
Dermal load (mg/cm^2)	0,001	mg/cm^2	O			
Absorption (mg)	1,98	mg	O			
Exposure per task / Acute Dose	0,033	mg/kg bw	O	no		S
Exposure per day / Chronic Dose	0,047013699	mg/kg bw/day	O	no		S
**Oral**						
Exposure / Dose	accidental		S	accidental		S
**Intake**						
Exposure per task / Acute Dose	0,033003384	mg/kg bw	O	7,09313E-06	mg/kg bw	O
Exposure per day / Chronic Dose	0,047018519	mg/kg bw/day	O	1,01053E-05	mg/kg bw/day	O

Scenario 15b: cleaning/disinfection: textiles, hand wash using laundry additive (liquid)

Scenario description

				Comments
Name of product	hygiene laundry rinse		S	
Physical state product (liquid/solid)	liquid		S	
Density product	1	g/cm³	D	
Concentration of active substance in product	1	%	S	*Frame Formulation*
Concentration of active substance in product (mg/l)	10000	mg/l	O	
User	Consumer		S	
Bystander	Children		S	
Temperature	20	°C	D	*room temperature*

Mixing & Loading

	Task A: simple dilution with water in vessel			
Number of tasks per year	208	tasks/yr	S	*four times a week, max [AISE (2002) cited in TGD (2003)]*
Duration of task	1	min/task	S	
Quantity of water used per task	20	L/task	S	*50% sink 40x40x25 cm*
Quantity of product used per task	200	g/task	S	*0.1 - 1% [AISE (2002) cited in TGD (2003)]*
Quantity of active substance used per task	2000	mg/task	O	
Concentration of active substance in washing solution	100	mg/l	O	
Model inhalation exposure	Exposure to vapour / Fugacity concept (equilibrium between washing solution and air, limited to vapour pressure)			
Room volume	1	m³	S	*cloud around user*
REM Evaporation from mixture, release area concentrate	*0,002*	*m²*	*S*	*bottle diameter 5 cm [TNsG 2002 p. 252]*
REM Evaporation from mixture, release area washing solution	*0,16*	*m²*	*S*	*surface vessel (40 x 40 cm)*
Model dermal exposure	Direct dermal contact			
Contact area between product (concentrate) and skin	0,0168	m²	S	*Fingertips*
Model oral exposure	accidental			

Application

	Task B: washing the textiles			
Number of tasks per year	208	tasks/yr	S	*four times a week, max [AISE (2002) cited in TGD (2003)]*
Duration of task	10	min/task	S	*[AISE (2002) cited in TGD (2003)]*
Quantity of water used per task	20	L/task	S	*50% of sink volume (40x40x25 cm)*
Quantity of product used per task	200	g/task	S	*0.1 - 1% [AISE (2002) cited in TGD (2003)]*
Quantity of active substance used per task	2000	mg/task	O	
Concentration of active substance in washing solution	100	mg/l	O	
Model inhalation exposure	Exposure to vapour / Fugacity concept (equilibrium between washing solution and air, limited to vapour pressure)			
Room volume	10	m³	S	*Bathroom*
REM Evaporation from mixture, release area washing solution	*0,16*	*m²*	*S*	*surface vessel (40 x 40 cm)*
Model dermal exposure	Direct dermal contact			
Contact area between washing solution and skin	0,198	m²	S	*Forearms, Hands*
Model oral exposure	accidental			

Post application phase

	Task C: wearing of clothes			
Number of tasks per year	208	tasks/yr	S	*four times a week*
Duration of task	720	min/task	S	*12 hours*
Weight fraction of product deposited on fabric	5	%	S	*HERA (worst case assumption)*
Fabric density	20	mg/cm²	S	*HERA (given for all cotton)*
Total weight of fabric	1	kg	S	*HERA (Estimation)*
Model inhalation exposure	Exposure to vapour / Instantaneous release (limited to vapour pressure)			
Room volume	1	m³	S	*cloud around user*
Model dermal exposure	Indirect dermal contact			
Contact area between textile and skin	1,94	m²	S	*Whole body, HERA: 1.76 m²*
Contact area between textile and skin children	0,3925	m²	S	*Whole body Children*
Weight fraction transferred from textile to skin, migration factor	1	%	S	*HERA (Vermeire et al. 1993)*
Model oral exposure	accidental			

Disposal

	Task D: excessive washing solution is disposed to the main drainage			
Number of tasks per year	208	tasks/yr	S	*four times a week, max [AISE (2002) cited in TGD (2003)]*
Duration of task	1	min/task	S	
Quantity of water disposed per task	20	L/task	S	*50% of sink volume (40x40x25 cm)*
Quantity of product disposed per task	190	g/task	S	0.1 - 1% [AISE (2002) cited in TGD (2003)]
Quantity of active substance disposed per task	1900	mg/task	S	
Concentration of active substance in washing solution	100	mg/l	O	
Model inhalation exposure	Exposure to vapour / Fugacity concept (equilibrium between washing solution and air, limited to vapour pressure)			
Room volume	1	m³	S	
REM Evaporation from mixture, release area washing solution	*0,16*	*m²*	*S*	*surface vessel (40 x 40 cm)*
Model dermal exposure	Direct dermal contact			
Contact area between washing solution and skin	0,198	m²	S	*Hands, Forearms*
Model oral exposure	accidental			

Summary Results Exposure

	User	Consumer		Bystander	Children	
Highest potential exposure acute (all amount used is absorbed)		33,33333333	mg/kg bw		240,9638554	mg/kg bw
Highest potential exposure chronic (all amount used is absorbed)		18,99543379	mg/kg bw/day		137,3163888	mg/kg bw/day
Highest potential concentration in air		100	mg/m³		100	mg/m³
Inhalation acute		1,666709224	mg/kg bw		12,04828199	mg/kg bw
Inhalation chronic		0,949795942	mg/kg bw/day		6,865870284	mg/kg bw/day
Dermal acute		0,352466667	mg/kg bw		0,009457831	mg/kg bw
Dermal chronic		0,200857717	mg/kg bw/day		0,005389668	mg/kg bw/day
Oral acute	accidental			accidental		
Oral chronic	accidental			accidental		
Intake acute		2,012709224	mg/kg bw		12,04828199	mg/kg bw
Daily intake chronic		1,146968544	mg/kg bw/day		6,865870284	mg/kg bw/day

U = Undefined, D = Default, S = Set, O = Output, REM = Remark, not used in calculation

Scenario 15b: cleaning/disinfection: textiles, hand wash using laundry additive (liquid)

Results / Output

Mixing & Loading	Task A: simple dilution with water in vessel					
	Primary exposure	User		Secondary exposure	Bystander	
**Inhalation**						
Concentration of potential exposure	0,020514722	mg/m^3	O	0,020514722	mg/m^3	O
Inhalation	0,000284927	mg/min	O	8,26287E-05	mg/min	O
Absorption per task	0,000213695	mg/task	O	6,19716E-05	mg/task	O
Exposure per task / Acute Dose	3,56158E-06	mg/kg bw	O	7,46645E-06	mg/kg bw	O
Exposure per day / Chronic Dose	2,02961E-06	mg/kg bw/day	O	4,25486E-06	mg/kg bw/day	O
**Dermal**						
Contact area between product (concentrate) and skin	0,0168	m^2	S			
Volume of contact	1,68	ml	O			
Quantity of contact (mg)	16,8	mg	O			
Dermal load (mg/cm^2)	0,1	mg/cm^2	O			
Absorption (mg)	16,8	mg	O			
Exposure per task / Acute Dose	0,28	mg/kg bw	O	no		S
Exposure per day / Chronic Dose	0,159561644	mg/kg bw/day	O	no		S
**Oral**						
Exposure / Dose	accidental		S	accidental		S
**Intake**						
Exposure per task / Acute Dose	0,280003562	mg/kg bw	O	7,46645E-06	mg/kg bw	O
Exposure per day / Chronic Dose	0,159563673	mg/kg bw/day	O	4,25486E-06	mg/kg bw/day	O

Application	Task B: washing the textiles					
	Primary exposure	User		Secondary exposure	Bystander	
**Inhalation**						
Concentration of potential exposure	0,020512828	mg/m^3	O	0,020512828	mg/m^3	O
Inhalation	0,0002849	mg/min	O	8,26211E-05	mg/min	O
Absorption per task	0,002136753	mg/task	O	0,000619658	mg/task	O
Exposure per task / Acute Dose	3,56125E-05	mg/kg bw	O	7,46576E-05	mg/kg bw	O
Exposure per day / Chronic Dose	2,02943E-05	mg/kg bw/day	O	4,25446E-05	mg/kg bw/day	O
**Dermal**						
Contact area between washing solution and skin	0,198	m^2	S			
Volume of contact	19,8	ml	O			
Quantity of contact (mg)	1,98	mg	O			
Dermal load (mg/cm^2)	0,001	mg/cm^2	O			
Absorption (mg)	1,98	mg	O			
Exposure per task / Acute Dose	0,033	mg/kg bw	O	no		S
Exposure per day / Chronic Dose	0,018805479	mg/kg bw/day	O	no		S
**Oral**						
Exposure / Dose	accidental		S	accidental		S
**Intake**						
Exposure per task / Acute Dose	0,033035613	mg/kg bw	O	7,46576E-05	mg/kg bw	O
Exposure per day / Chronic Dose	0,018825774	mg/kg bw/day	O	4,25446E-05	mg/kg bw/day	O

Post application phase	Task C: wearing of clothes					
	Primary exposure	User		Secondary exposure	Bystander	
**Inhalation**						
Concentration of potential exposure	100	mg/m^3	O	100	mg/m^3	O
Inhalation	1,388888889	mg/min	O	0,402777778	mg/min	O
Absorption per task	100	mg/task	S	100	mg/task	S
Exposure per task / Acute Dose	1,666666667	mg/kg bw	O	12,04819277	mg/kg bw	O
Exposure per day / Chronic Dose	0,949771689	mg/kg bw/day	O	6,865819442	mg/kg bw/day	O
**Dermal**						
Contact area between textile and skin	1,94	m^2	S	0,3925	m^2	S
Dermal load (mg/cm^2)	0,002	mg/cm^2	O	0,002	mg/cm^2	O
Absorption (mg)	0,388	mg	O	0,0785	mg	O
Exposure per task / Acute Dose	0,006466667	mg/kg bw	O	0,009457831	mg/kg bw	O
Exposure per day / Chronic Dose	0,003685114	mg/kg bw/day	O	0,005389668	mg/kg bw/day	O
**Oral**						
Exposure / Dose	accidental		S	accidental		S
**Intake**						
Exposure per task / Acute Dose	1,666666667	mg/kg bw	S	12,04819277	mg/kg bw	S
Exposure per day / Chronic Dose	0,949771689	mg/kg bw/day	O	6,865819442	mg/kg bw/day	O

Disposal	Task D: excessive washing solution is disposed to the main drainage					
	Primary exposure	User		Secondary exposure	Bystander	
**Inhalation**						
Concentration of potential exposure	0,019488986	mg/m^3	O	0,019488986	mg/m^3	O
Inhalation	0,00027068	mg/min	O	7,84973E-05	mg/min	O
Absorption per task	0,00020301	mg/task	O	5,8873E-05	mg/task	O
Exposure per task / Acute Dose	3,3835E-06	mg/kg bw	O	7,09313E-06	mg/kg bw	O
Exposure per day / Chronic Dose	1,92813E-06	mg/kg bw/day	O	4,04211E-06	mg/kg bw/day	O
**Dermal**						
Contact area between washing solution and skin	0,198	m^2	S			
Volume of contact	19,8	ml	O			
Quantity of contact (mg)	1,98	mg	O			
Dermal load (mg/cm^2)	0,001	mg/cm^2	O			
Absorption (mg)	1,98	mg	O			
Exposure per task / Acute Dose	0,033	mg/kg bw	O	no		S
Exposure per day / Chronic Dose	0,018805479	mg/kg bw/day	O	no		S
**Oral**						
Exposure / Dose	accidental		S	accidental		S
**Intake**						
Exposure per task / Acute Dose	0,033003384	mg/kg bw	O	7,09313E-06	mg/kg bw	O
Exposure per day / Chronic Dose	0,018807408	mg/kg bw/day	O	4,04211E-06	mg/kg bw/day	O

U = Undefined, D = Default, S = Set, O = Output, REM = Remark, not used in calculation

Scenario 16: textiles, wearing clothes with antimicrobial protection

Scenario description

				Comments
Name of product	antimicrobial protection		S	
User	Consumer		S	
User 2	Children		S	
Temperature	20	°C	D	*room temperature*

Mixing & Loading

Task A: not applicable

Application

Task B: not applicable

Post application phase

Task C: wearing of clothes

Number of tasks per year	52	tasks/yr	S	*once a week*
Duration of task	720	min/task	S	*12 hours*
Loading of fabric	500	mg/kg	S	
Fabric density	20	mg/cm^2	S	*HERA (given for all cotton)*
Total weight of fabric	1	kg	S	*HERA (Estimation)*
Model inhalation exposure	Exposure to vapour / Instantaneous release (limited to vapour pressure)			
Room volume	1	m^3	S	*cloud around user*
Model dermal exposure	Indirect dermal contact			
Contact area between textile and skin	1,94	m^2	S	*Whole body, HERA: 1.76 m^2*
Contact area between textile and skin children	0,3925	m^2	S	*Whole body Children*
Weight fraction transferred from textile to skin, migration factor	1	%	S	*HERA (Vermeire et al. 1993)*
Model oral exposure	accidental			

Disposal

Task D: not applicable

Summary Results Exposure

	User	Consumer		User 2	Children	
Highest potential exposure acute (all amount used is absorbed)		8,333333333	mg/kg bw		60,24096386	mg/kg bw
Highest potential exposure chronic (all amount used is absorbed)		1,187214612	mg/kg bw/day		8,582274303	mg/kg bw/day
Highest potential concentration in air		205,1493218	mg/m^3		205,1493218	mg/m^3
Inhalation acute		8,333333333	mg/kg bw		53,75900903	mg/kg bw
Inhalation chronic		1,187214612	mg/kg bw/day		7,658817725	mg/kg bw/day
Dermal acute		0,032333333	mg/kg bw		0,047289157	mg/kg bw
Dermal chronic		0,004606393	mg/kg bw/day		0,006737085	mg/kg bw/day
Oral acute	accidental			accidental		
Oral chronic	accidental			accidental		
Intake acute		8,333333333	mg/kg bw		53,80629819	mg/kg bw
Daily intake chronic		1,187214612	mg/kg bw/day		7,665554811	mg/kg bw/day

U = Undefined, D = Default, S = Set, O = Output, REM = Remark, not used in calculation

Scenario 16: textiles, wearing clothes with antimicrobial protection

Results / Output

**Mixing & Loading**	Task A: not applicable					

**Application**	Task B: not applicable					

**Post application phase**	Task C: wearing of clothes					
	**Primary exposure**	**User**		**Secondary exposure**	**Bystander**	
**Inhalation**						
Concentration of potential exposure	205,1493218	mg/m^3	S	205,1493218	mg/m^3	S
Inhalation	2,849296137	mg/min	O	0,82629588	mg/min	O
Absorption per task	500	mg/task	S	446,199775	mg/task	O
Exposure per task / Acute Dose	8,333333333	mg/kg bw	O	53,75900903	mg/kg bw	O
Exposure per day / Chronic Dose	1,187214612	mg/kg bw/day	O	7,658817725	mg/kg bw/day	O
**Dermal**						
Contact area between textile and skin	1,94	m^2	S	0,3925	m^2	S
Dermal load (mg/cm^2)	0,01	mg/cm^2	O	0,01	mg/cm^2	O
Absorption (mg)	1,94	mg	O	0,3925	mg	O
Exposure per task / Acute Dose	0,032333333	mg/kg bw	O	0,047289157	mg/kg bw	O
Exposure per day / Chronic Dose	0,004606393	mg/kg bw/day	O	0,006737085	mg/kg bw/day	O
**Oral**						
Exposure / Dose	accidental		S	accidental		S
**Intake**						
Exposure per task / Acute Dose	8,333333333	mg/kg bw	S	53,80629819	mg/kg bw	O
Exposure per day / Chronic Dose	1,187214612	mg/kg bw/day	O	7,665554811	mg/kg bw/day	O

**Disposal**	Task D: not applicable					

U = Undefined, D = Default, S = Set, O = Output, REM = Remark, not used in calculation

Scenario 17: cleaning/disinfection: machine dishwashing (tablet, powder)

Scenario description				*Comments*
Name of product	diswashing powder		S	
Physical state product (liquid/solid)	solid		S	
Density product	1	g/cm^3	D	
Concentration of active substance in product	1	%	S	*Frame Formulation*
Concentration of active substance in product (mg/l)	10000	mg/l	O	
User	Consumer		S	
Bystander	Children		S	
Temperature	20	°C	D	*room temperature*
**Mixing & Loading**	Task A: fill in			
Number of tasks per year	365	tasks/yr	S	*once a day, max [AISE (2002) cited in TGD (2003)]*
Duration of task	1	min/task	S	*< 1 min [AISE (2002) cited in TGD (2003)]]*
Quantity of product used per task	40	g/task	S	*max [AISE (2002) cited in TGD (2003)]*
Quantity of active substance used per task	400	mg/task	O	
Volume of product used per task	40	ml/task	O	
Model inhalation exposure	Exposure to vapour / Instantaneous release (limited to vapour pressure)			
Room volume	1	m^3	S	*cloud around user*
REM Evaporation from mixture, release area	*0,002*	*m^2*	*S*	*bottle diameter 5 cm [TNsG 2002 p. 252]*
Model dermal exposure	Direct dermal contact			
Contact area between product and skin	0,0168	m^2	S	*Fingertips*
Contact amount between active substance and skin	0,4	mg/task	S	*0.1% of whole amount used [see HERA Perborate]*
Model oral exposure	accidental			
**Application**	Task B: no inhalative or dermal contact during dishwashing procedure -> not applicable			
**Post application phase**	Task C: Substance deposited on surface of dishes, then swallowed via food or drink			
Number of tasks per year	365	tasks/yr	S	*HERA*
Wash cycles per wash	4	-	S	*HERA (Miele 2002): 3-4 wash cycles*
Amount of water per wash cycle	4,8	L	S	*HERA (Miele 2002): 4.6-4.8 Litres*
Amount of wash-solution transferred to next wash cycle	0,6	L	S	*HERA (Miele 2002): 0.5-0.6 Litres*
Concentration of active substance in wash-solution (last cycle)	0,162760417	mg/l	O	
Model inhalation exposure	Exposure to vapour / negligible compared with oral exposure			
Model dermal exposure	Direct dermal contact / negligible compared with oral exposure			
Model oral exposure	Indirect oral ingestion			
Amount of liquor remaining on surface	5,5E-04	ml/cm^2	S	*HERA (O.J. France 1990)*
Load of active substance on surface of article	9,0E-08	mg/cm^2	O	
Area of dishes/eating utensils in daily contact with food	5400	cm^2	S	*HERA (O.J. France 1990)*
Weight fraction transferred from article and ingested	100	%	S	*HERA (worst-case assumption)*
**Disposal**	Task D: not applicable			

Summary Results Exposure

	User	Consumer		Bystander	Children	
Highest potential exposure acute (all amount used is absorbed)		6,666666667	mg/kg bw		48,19277108	mg/kg bw
Highest potential exposure chronic (all amount used is absorbed)		6,666666667	mg/kg bw/day		48,19277108	mg/kg bw/day
Highest potential concentration in air		205,1493218	mg/m^3		205,1493218	mg/m^3
Inhalation acute		0,035616202	mg/kg bw		0,07466529	mg/kg bw
Inhalation chronic		0,035616202	mg/kg bw/day		0,07466529	mg/kg bw/day
Dermal acute		0,006666667	mg/kg bw		0	mg/kg bw
Dermal chronic		0,006666667	mg/kg bw/day		0	mg/kg bw/day
Oral acute		8,05664E-06	mg/kg bw		5,82408E-05	mg/kg bw
Oral chronic		8,05664E-06	mg/kg bw/day		5,82408E-05	mg/kg bw/day
Intake acute		0,042290925	mg/kg bw		0,074723531	mg/kg bw
Daily intake chronic		0,042290925	mg/kg bw/day		0,074723531	mg/kg bw/day

U = Undefined, D = Default, S = Set, O = Output, REM = Remark, not used in calculation

Scenario 17: cleaning/disinfection: machine dishwashing (tablet, powder)

Results / Output

**Mixing & Loading**	Task A: fill in					
**Inhalation**						
Concentration of potential exposure	205,1493218	mg/m^3	S	205,1493218	mg/m^3	S
Inhalation	2,849296137	mg/min	O	0,82629588	mg/min	O
Absorption per task	2,136972102	mg/task	O	0,61972191	mg/task	O
Exposure per task / Acute Dose	0,035616202	mg/kg bw	O	0,07466529	mg/kg bw	O
Exposure per day / Chronic Dose	0,035616202	mg/kg bw/day	O	0,07466529	mg/kg bw/day	O
**Dermal**						
Contact area between product and skin	0,0168	m^2	S			
Quantity of contact	0,4	mg	S			
Dermal load (mg/cm^2)	0,002380952	mg/cm^2	O			
Absorption (mg)	0,4	mg	O			
Exposure per task / Acute Dose	0,006666667	mg/kg bw	O	no		S
Exposure per day / Chronic Dose	0,006666667	mg/kg bw/day	O	no		S
**Oral**						
Exposure / Dose	accidental		S	accidental		S
**Intake**						
Exposure per task / Acute Dose	0,042282868	mg/kg bw	O	0,07466529	mg/kg bw	O
Exposure per day / Chronic Dose	0,042282868	mg/kg bw/day	O	0,07466529	mg/kg bw/day	O

**Application**	Task B: no inhalative or dermal contact during dishwashing procedure -> not applicable

**Post application phase**	Task C: Substance deposited on surface of dishes, then swallowed via food or drink					
	**Primary exposure**	**User**		**Secondary exposure**	**Bystander**	
**Inhalation**						
Exposure per task / Acute Dose	negligible	mg/kg bw	S	negligible	mg/kg bw	S
Exposure per day / Chronic Dose	negligible	mg/kg bw/day	S	negligible	mg/kg bw/day	S
**Dermal**						
Exposure per task / Acute Dose	negligible	mg/kg bw	S	negligible	mg/kg bw	S
Exposure per day / Chronic Dose	negligible	mg/kg bw/day	S	negligible	mg/kg bw/day	S
**Oral**						
Absorption per task	0,000483398	mg/task	O	0,000483398	mg/task	O
Exposure per task / Acute Dose	8,05664E-06	mg/kg bw	O	5,82408E-05	mg/kg bw	O
Exposure per day / Chronic Dose	8,05664E-06	mg/kg bw/day	O	5,82408E-05	mg/kg bw/day	O
**Intake**						
Exposure per task / Acute Dose	8,05664E-06	mg/kg bw	O	5,82408E-05	mg/kg bw	O
Exposure per day / Chronic Dose	8,05664E-06	mg/kg bw/day	O	5,82408E-05	mg/kg bw/day	O

**Disposal**	Task D: not applicable

U = Undefined, D = Default, S = Set, O = Output, REM = Remark, not used in calculation

Scenario 17b: cleaning/disinfection: machine dishwashing (liquid)

Scenario description

				Comments
Name of product	diswashing liquid		S	
Physical state product (liquid/solid)	liquid		S	
Density product	1	g/cm^3	D	
Concentration of active substance in product	1	%	S	*Frame Formulation*
Concentration of active substance in product (mg/l)	10000	mg/l	O	
User	Consumer		S	
Bystander	Children		S	
Temperature	20	°C	D	*room temperature*
**Mixing & Loading**	Task A: fill in			
Number of tasks per year	365	tasks/yr	S	*once a day, max [AISE (2002) cited in TGD (2003)]*
Duration of task	1	min/task	S	*< 1 min [AISE (2002) cited in TGD (2003)]]*
Quantity of product used per task	40	g/task	S	*max [AISE (2002) cited in TGD (2003)]*
Quantity of active substance used per task	400	mg/task	O	
Volume of product used per task	40	ml/task	O	
Model inhalation exposure	Exposure to vapour / Fugacity concept (equilibrium between product/solution and air, limited to vapour pressure)			
Room volume	1	m^3	S	*cloud around user*
REM Evaporation from mixture, release area	*0,002*	*m^2*	*S*	*bottle diameter 5 cm [TNsG 2002 p. 252]*
Model dermal exposure	Direct dermal contact			
Contact area between product and skin	0,0168	m^2	S	*Fingertips*
Model oral exposure	accidental			
**Application**	Task B: no inhalative or dermal contact during dishwashing procedure -> not applicable			
**Post application phase**	Task C: Substance deposited on surface of dishes, then swallowed via food or drink			
Number of tasks per year	365	tasks/yr	S	*HERA*
Wash cycles per wash	4	-	S	*HERA (Miele 2002): 3-4 wash cycles*
Amount of water per wash cycle	4,8	L	S	*HERA (Miele 2002): 4.6-4.8 Litres*
Amount of wash-solution transferred to next wash cycle	0,6	L	S	*HERA (Miele 2002): 0.5-0.6 Litres*
Concentration of active substance in wash-solution (last cycle)	0,162760417	mg/l	O	
Model inhalation exposure	Exposure to vapour / negligible compared with oral exposure			
Model dermal exposure	Direct dermal contact / negligible compared with oral exposure			
Model oral exposure	Indirect oral ingestion			
Amount of liquor remaining on surface	5,5E-04	ml/cm^2	S	*HERA (O.J. France 1990)*
Load of active substance on surface of article	9,0E-08	mg/cm^2	O	
Area of dishes/eating utensils in daily contact with food	5400	cm^2	S	*HERA (O.J. France 1990)*
Weight fraction transferred from article and ingested	100	%	S	*HERA (worst-case assumption)*
**Disposal**	Task D: not applicable			

Summary Results Exposure

	User	Consumer		Bystander	Children
Highest potential exposure acute (all amount used is absorbed)		6,666666667	mg/kg bw	48,19277108	mg/kg bw
Highest potential exposure chronic (all amount used is absorbed)		6,666666667	mg/kg bw/day	48,19277108	mg/kg bw/day
Highest potential concentration in air		2,041025344	mg/m^3	2,041025344	mg/m^3
Inhalation acute		0,000354345	mg/kg bw	0,000742843	mg/kg bw
Inhalation chronic		0,000354345	mg/kg bw/day	0,000742843	mg/kg bw/day
Dermal acute		0,28	mg/kg bw	0	mg/kg bw
Dermal chronic		0,28	mg/kg bw/day	0	mg/kg bw/day
Oral acute		8,05664E-06	mg/kg bw	5,82408E-05	mg/kg bw
Oral chronic		8,05664E-06	mg/kg bw/day	5,82408E-05	mg/kg bw/day
Intake acute		0,280362401	mg/kg bw	0,000801084	mg/kg bw
Daily intake chronic		0,280362401	mg/kg bw/day	0,000801084	mg/kg bw/day

U = Undefined, D = Default, S = Set, O = Output, REM = Remark, not used in calculation

Scenario 17b: cleaning/disinfection: machine dishwashing (liquid)

Results / Output

Mixing & Loading	Task A: fill in					
**Inhalation**						
Concentration of potential exposure	2,041025344	mg/m³	O	2,041025344	mg/m³	O
Inhalation	0,028347574	mg/min	O	0,008220797	mg/min	O
Absorption per task	0,021260681	mg/task	O	0,006165597	mg/task	O
Exposure per task / Acute Dose	0,000354345	mg/kg bw	O	0,000742843	mg/kg bw	O
Exposure per day / Chronic Dose	0,000354345	mg/kg bw/day	O	0,000742843	mg/kg bw/day	O
**Dermal**						
Contact area between product and skin	0,0168	m²	S			
Volume of contact	1,68	ml	O			
Quantity of contact	16,8	mg	O			
Dermal load (mg/cm²)	0,1	mg/cm²	O			
Absorption (mg)	16,8	mg	O			
Exposure per task / Acute Dose	0,28	mg/kg bw	O	no		S
Exposure per day / Chronic Dose	0,28	mg/kg bw/day	O	no		S
**Oral**						
Exposure / Dose	accidental		S	accidental		S
**Intake**						
Exposure per task / Acute Dose	0,280354345	mg/kg bw	O	0,000742843	mg/kg bw	O
Exposure per day / Chronic Dose	0,280354345	mg/kg bw/day	O	0,000742843	mg/kg bw/day	O

Application	Task B: no inhalative or dermal contact during dishwashing procedure -> not applicable

Post application phase	Task C: Substance deposited on surface of dishes, then swallowed via food or drink					
	**Primary exposure**	**User**		**Secondary exposure**	**Bystander**	
**Inhalation**						
Exposure per task / Acute Dose	negligible	mg/kg bw	S	negligible	mg/kg bw	S
Exposure per day / Chronic Dose	negligible	mg/kg bw/day	S	negligible	mg/kg bw/day	S
**Dermal**						
Exposure per task / Acute Dose	negligible	mg/kg bw	S	negligible	mg/kg bw	S
Exposure per day / Chronic Dose	negligible	mg/kg bw/day	S	negligible	mg/kg bw/day	S
**Oral**						
Absorption per task	0,000483398	mg/task	O	0,000483398	mg/task	O
Exposure per task / Acute Dose	8,05664E-06	mg/kg bw	O	5,82408E-05	mg/kg bw	O
Exposure per day / Chronic Dose	8,05664E-06	mg/kg bw/day	O	5,82408E-05	mg/kg bw/day	O
**Intake**						
Exposure per task / Acute Dose	8,05664E-06	mg/kg bw	O	5,82408E-05	mg/kg bw	O
Exposure per day / Chronic Dose	8,05664E-06	mg/kg bw/day	O	5,82408E-05	mg/kg bw/day	O

Disposal	Task D: not applicable

U = Undefined, D = Default, S = Set, O = Output, REM = Remark, not used in calculation

Scenario 17c: cleaning/disinfection: hand dishwashing (liquid concentrate)

Scenario description

				Comments
Name of product	diswashing liquid		S	
Physical state product (liquid/solid)	liquid		S	
Density product	1	g/cm^3	D	
Concentration of active substance in product	1	%	S	*Frame Formulation*
Concentration of active substance in product (mg/l)	10000	mg/l	O	
User	Consumer		S	
Bystander	Children		S	
Temperature	20	°C	D	*room temperature*
**Mixing & Loading**	Task A: simple dilution with water in the sink			
Number of tasks per year	1095	tasks/yr	S	*three times a day, max [AISE (2002) cited in TGD (2003)]*
Duration of task	1	min/task	S	
Quantity of water used per task	20	L/task	S	*50% sink 40x40x25 cm*
Quantity of product used per task	20	g/task	S	*max 5 g/5 L/task [AISE (2002) cited in TGD (2003)]*
Quantity of active substance used per task	200	mg/task	O	
Concentration of active substance in dishwashing water	10	mg/l	O	
Model inhalation exposure	Exposure to vapour / Fugacity concept (equilibrium between dishwashing solution and air, limited to vapour pressure)			
Room volume	1	m^3	S	*cloud around user*
REM Evaporation from mixture, release area concentrate	*0,00002*	*m^2*	*S*	*bottle diameter 0.5 cm*
REM Evaporation from mixture, release area dishwashing water	*0,16*	*m^2*	*S*	*surface sink 40x40 cm*
Model dermal exposure	Direct dermal contact			
Contact area between product (concentrate) and skin	0,0168	m^2	S	*Fingertips*
Model oral exposure	accidental			
**Application**	Task B: washing-up dishes			
Number of tasks per year	1095	tasks/yr	S	*three times a day, max [AISE (2002) cited in TGD (2003)]*
Duration of task	45	min/task	S	*max [AISE (2002) cited in TGD (2003)]*
Quantity of water used per task	20	L/task	S	*50% sink 40x40x25 cm*
Quantity of product used per task	20	g/task	S	max 5 g/5 L/task [AISE (2002) cited in TGD (2003)]
Quantity of active substance used per task	200	mg/task	O	
Concentration of active substance in dishwashing water	10	mg/l	O	
Model inhalation exposure	Exposure to vapour / Fugacity concept (equilibrium between dishwashing solution and air, limited to vapour pressure)			
Room volume	15	m^3	S	*Kitchen*
REM Evaporation from mixture, release area dishwashing water	*0,16*	*m^2*	*S*	*surface sink 40x40 cm*
Model dermal exposure	Direct dermal contact			
Contact area between dishwashing water and skin	0,198	m^2	S	*Forearms, Hands [HERA]*
Model oral exposure	accidental			
**Post application phase**	Task C: Substance deposited on surface of dishes, then swallowed via food or drink			
Number of tasks per year	365	tasks/yr	S	*HERA*
Concentration of active substance in dishwashing water	10	mg/l	O	
Model inhalation exposure	Exposure to vapour / negligible compared with oral exposure			
Model dermal exposure	Direct dermal contact / negligible compared with oral exposure			
Model oral exposure	Indirect oral ingestion			
Amount of liquor remaining on surface	5,5E-04	ml/cm^2	S	*HERA (O.J. France 1990)*
Percent of liquor left after rinsing	100	%	S	*no rinsing*
Load of active substance on surface of article	0,0000055	mg/cm^2	O	
Area of dishes/eating utensils in daily contact with food	5400	cm^2	S	*HERA (O.J. France 1990)*
Weight fraction transferred from article and ingested	100	%	S	*HERA (worst-case assumption)*
**Disposal**	Task D: not applicable			

Summary Results Exposure

	User	Consumer		Bystander	Children	
Highest potential exposure acute (all amount used is absorbed)		3,333333333	mg/kg bw		24,09638554	mg/kg bw
Highest potential exposure chronic (all amount used is absorbed)		10	mg/kg bw/day		72,28915663	mg/kg bw/day
Highest potential concentration in air		0,002051472	mg/m^3		0,002051472	mg/m^3
Inhalation acute		1,6381E-05	mg/kg bw		3,43409E-05	mg/kg bw
Inhalation chronic		4,9143E-05	mg/kg bw/day		0,000103023	mg/kg bw/day
Dermal acute		0,2833	mg/kg bw		0	mg/kg bw
Dermal chronic		0,8499	mg/kg bw/day		0	mg/kg bw/day
Oral acute		0,000495	mg/kg bw		0,003578313	mg/kg bw
Oral chronic		0,000495	mg/kg bw/day		0,003578313	mg/kg bw/day
Intake acute		0,283811381	mg/kg bw		0,003612654	mg/kg bw
Daily intake chronic		0,850444143	mg/kg bw/day		0,003681336	mg/kg bw/day

U = Undefined, D = Default, S = Set, O = Output, REM = Remark, not used in calculation

Scenario 17c: cleaning/disinfection: hand dishwashing (liquid concentrate)

Results / Output

**Mixing & Loading**	Task A: simple dilution with water in the sink					
	**Primary exposure**	**User**		**Secondary exposure**	**Bystander**	
**Inhalation**						
Concentration of potential exposure	0,002051472	mg/m^3	O	0,002051472	mg/m^3	O
Inhalation	2,84927E-05	mg/min	O	8,26287E-06	mg/min	O
Absorption per task	2,13695E-05	mg/task	O	6,19716E-06	mg/task	O
Exposure per task / Acute Dose	3,56158E-07	mg/kg bw	O	7,46645E-07	mg/kg bw	O
Exposure per day / Chronic Dose	1,06848E-06	mg/kg bw/day	O	2,23994E-06	mg/kg bw/day	O
**Dermal**						
Contact area between product (concentrate) and skin	0,0168	m^2	S			
Volume of contact	1,68	ml	O			
Quantity of contact	16,8	mg	O			
Dermal load (mg/cm^2)	0,1	mg/cm^2	O			
Absorption (mg)	16,8	mg	O			
Exposure per task / Acute Dose	0,28	mg/kg bw	O	no		S
Exposure per day / Chronic Dose	0,84	mg/kg bw/day	O	no		S
**Oral**						
Exposure / Dose	accidental		S	accidental		S
**Intake**						
Exposure per task / Acute Dose	0,280000356	mg/kg bw	O	7,46645E-07	mg/kg bw	O
Exposure per day / Chronic Dose	0,840001068	mg/kg bw/day	O	2,23994E-06	mg/kg bw/day	O

**Application**	Task B: washing-up dishes					
	**Primary exposure**	**User**		**Secondary exposure**	**Bystander**	
**Inhalation**						
Concentration of potential exposure	0,002051178	mg/m^3	O	0,002051178	mg/m^3	O
Inhalation	2,84886E-05	mg/min	O	8,26169E-06	mg/min	O
Absorption per task	0,00096149	mg/task	O	0,000278832	mg/task	O
Exposure per task / Acute Dose	1,60248E-05	mg/kg bw	O	3,35942E-05	mg/kg bw	O
Exposure per day / Chronic Dose	4,80745E-05	mg/kg bw/day	O	0,000100783	mg/kg bw/day	O
**Dermal**						
Contact area between dishwashing water and skin	0,198	m^2	S			
Volume of contact	19,8	ml	O			
Quantity of contact	0,198	mg	O			
Dermal load (mg/cm^2)	0,0001	mg/cm^2	O			
Absorption (mg)	0,198	mg	O			
Exposure per task / Acute Dose	0,0033	mg/kg bw	O	no		S
Exposure per day / Chronic Dose	0,0099	mg/kg bw/day	O	no		S
**Oral**						
Exposure / Dose	accidental		S	accidental		S
**Intake**						
Exposure per task / Acute Dose	0,003316025	mg/kg bw	O	3,35942E-05	mg/kg bw	O
Exposure per day / Chronic Dose	0,009948074	mg/kg bw/day	O	0,000100783	mg/kg bw/day	O

**Post application phase**	Task C: Substance deposited on surface of dishes, then swallowed via food or drink					
	**Primary exposure**	**User**		**Secondary exposure**	**Bystander**	
**Inhalation**						
Exposure per task / Acute Dose	negligible	mg/kg bw	S	negligible	mg/kg bw	S
Exposure per day / Chronic Dose	negligible	mg/kg bw/day	S	negligible	mg/kg bw/day	S
**Dermal**						
Exposure per task / Acute Dose	negligible	mg/kg bw	S	negligible	mg/kg bw	S
Exposure per day / Chronic Dose	negligible	mg/kg bw/day	S	negligible	mg/kg bw/day	S
**Oral**						
Absorption per task	0,0297	mg/task	O	0,0297	mg/task	O
Exposure per task / Acute Dose	0,000495	mg/kg bw	O	0,003578313	mg/kg bw	O
Exposure per day / Chronic Dose	0,000495	mg/kg bw/day	O	0,003578313	mg/kg bw/day	O
**Intake**						
Exposure per task / Acute Dose	0,000495	mg/kg bw	O	0,003578313	mg/kg bw	O
Exposure per day / Chronic Dose	0,000495	mg/kg bw/day	O	0,003578313	mg/kg bw/day	O

**Disposal**	Task D: not applicable

U = Undefined, D = Default, S = Set, O = Output, REM = Remark, not used in calculation

Scenario 18: personal care: whole body rinse-off

Scenario description

				Comments
Name of product	shower gel		S	
Physical state product (liquid/solid)	liquid		S	
Density product	1	g/cm^3	D	
Concentration of active substance in product	1	%	S	*Frame Formulation*
Concentration of active substance in product (mg/l)	10000	mg/l	O	
User	Consumer		S	
User 2	Children		S	
Temperature	20	°C	D	*room temperature*

Mixing & Loading

Task A: not applicable

Application

Task B: shampooing under shower, washing with shower gel

Number of tasks per year	365	tasks/yr	S	*once per day [TGD (2003)]*
Duration of task	4	min/task	S	*ConsExpo*
Quantity of product used per task	12	g/task	S	*shampoo [TGD (2003)], shower gel 5 g but twice a day*
Volume of product used per task	12	ml/task	O	
Quantity of active substance used per task	120	mg/task	O	
Model inhalation exposure	Exposure to vapour / Instantaneous release (limited to vapour pressure)			
Room volume	1	m^3	S	*cloud around user*
Model dermal exposure	Direct dermal contact			
Contact area between product and skin	1,94	m^2	S	*Whole body*
Contact area between product and skin children	0,3925	m^2	S	*Whole body children*
Contact amount between product and skin	12	g/task	S	*Whole amount used*
Model oral exposure	accidental			

Post application phase

Task C: rinse-off product -> not applicable

Disposal

Task D: not applicable

Summary Results Exposure

	User	Consumer		User 2	Children	
Highest potential exposure acute (all amount used is absorbed)		2	mg/kg bw		14,45783133	mg/kg bw
Highest potential exposure chronic (all amount used is absorbed)		2	mg/kg bw/day		14,45783133	mg/kg bw/day
Highest potential concentration in air		120	mg/m^3		120	mg/m^3
Inhalation acute		0,083333333	mg/kg bw		0,174698795	mg/kg bw
Inhalation chronic		0,083333333	mg/kg bw/day		0,174698795	mg/kg bw/day
Dermal acute		2	mg/kg bw		14,45783133	mg/kg bw
Dermal chronic		2	mg/kg bw/day		14,45783133	mg/kg bw/day
Oral acute	accidental			accidental		
Oral chronic	accidental			accidental		
Intake acute		2	mg/kg bw		14,45783133	mg/kg bw
Daily intake chronic		2	mg/kg bw/day		14,45783133	mg/kg bw/day

U = Undefined, D = Default, S = Set, O = Output, REM = Remark, not used in calculation

Scenario 18: personal care: whole body rinse-off

Results / Output

Mixing & Loading	Task A: not applicable					

**Application**	Task B: shampooing under shower, washing with shower gel					
	**Primary exposure**	**User**		**Secondary exposure**	**Bystander**	
**Inhalation**						
Concentration of potential exposure	120	mg/m³	O	120	mg/m³	O
Inhalation	1,666666667	mg/min	O	0,483333333	mg/min	O
Absorption per task	5	mg/task	O	1,45	mg/task	O
Exposure per task / Acute Dose	0,083333333	mg/kg bw	O	0,174698795	mg/kg bw	O
Exposure per day / Chronic Dose	0,083333333	mg/kg bw/day	O	0,174698795	mg/kg bw/day	O
**Dermal**						
Contact area between product and skin	1,94	m^2	S	0,3925	m^2	S
Quantity of contact (mg)	120	mg	S	120	mg	S
Dermal load (mg/cm²)	0,006185567	mg/cm^2	O	0,030573248	mg/cm^2	O
Absorption (mg)	120	mg	O	120	mg	O
Exposure per task / Acute Dose	2	mg/kg bw	O	14,45783133	mg/kg bw	O
Exposure per day / Chronic Dose	2	mg/kg bw/day	O	14,45783133	mg/kg bw/day	O
**Oral**						
Exposure / Dose	accidental		S	accidental		S
**Intake**						
Exposure per task / Acute Dose	2	mg/kg bw	S	14,45783133	mg/kg bw	S
Exposure per day / Chronic Dose	2	mg/kg bw/day	O	14,45783133	mg/kg bw/day	O

Post application phase	Task C: rinse-off product -> not applicable

Disposal	Task D: not applicable

Scenario 19: personal care: whole body leave-on

Scenario description

				Comments
Name of product	body lotion		S	
Physical state product (liquid/solid)	liquid		S	
Density product	1	g/cm^3	D	
Concentration of active substance in product	1	%	S	*Frame Formulation*
Concentration of active substance in product (mg/l)	10000	mg/l	O	
User	Consumer		S	
User 2	Children		S	
Temperature	20	°C	D	*room temperature*

Mixing & Loading

Task A: not applicable

Application + Post application phase

Task B: rubbing in body lotion + leave-on

Number of tasks per year	730	tasks/yr	S	*twice per day [TGD (2003), ConsExpo]*
Duration of task	720	min/task	S	*ConsExpo: 720 min*
Quantity of product used per task	7,5	g/task	S	*[TGD (2003)], ConsExpo 8 g*
Volume of product used per task	7,5	ml/task	O	
Quantity of active substance used per task	75	mg/task	O	
Model inhalation exposure	Exposure to vapour / Instantaneous release (limited to vapour pressure)			
Room volume	1	m^3	S	*cloud around user*
Model dermal exposure	Direct dermal contact			
Contact area between product and skin	1,94	m^2	S	*Whole body*
Contact area between product and skin children	0,3925	m^2	S	*Whole body children*
Contact amount between product and skin	7,5	g/task	S	*Whole amount used*
Model oral exposure	accidental			

Disposal

Task D: not applicable

Summary Results Exposure

	User	Consumer		User 2	Children	
Highest potential exposure acute (all amount used is absorbed)		1,25	mg/kg bw		9,036144578	mg/kg bw
Highest potential exposure chronic (all amount used is absorbed)		2,5	mg/kg bw/day		18,07228916	mg/kg bw/day
Highest potential concentration in air		75	mg/m^3		75	mg/m^3
Inhalation acute		1,25	mg/kg bw		9,036144578	mg/kg bw
Inhalation chronic		2,5	mg/kg bw/day		18,07228916	mg/kg bw/day
Dermal acute		1,25	mg/kg bw		9,036144578	mg/kg bw
Dermal chronic		2,5	mg/kg bw/day		18,07228916	mg/kg bw/day
Oral acute	accidental			accidental		
Oral chronic	accidental			accidental		
Intake acute		1,25	mg/kg bw		9,036144578	mg/kg bw
Daily intake chronic		2,5	mg/kg bw/day		18,07228916	mg/kg bw/day

U = Undefined, D = Default, S = Set, O = Output, REM = Remark, not used in calculation

Scenario 19: personal care: whole body leave-on

Results / Output

**Mixing & Loading**	Task A: not applicable					

**Application + Post application phase**	Task B: rubbing in body lotion + leave-on					
	**Primary exposure**	**User**		**Secondary exposure**	**Bystander**	
**Inhalation**						
Concentration of potential exposure	75	mg/m^3	O	75	mg/m^3	O
Inhalation	1,041666667	mg/min	O	0,302083333	mg/min	O
Absorption per task	75	mg/task	S	75	mg/task	S
Exposure per task / Acute Dose	1,25	mg/kg bw	O	9,036144578	mg/kg bw	O
Exposure per day / Chronic Dose	2,5	mg/kg bw/day	O	18,07228916	mg/kg bw/day	O
**Dermal**						
Contact area between product and skin	1,94	m^2	S	0,3925	m^2	S
Quantity of contact (mg)	75	mg	S	75	mg	S
Dermal load (mg/cm^2)	0,003865979	mg/cm^2	O	0,01910828	mg/cm^2	O
Absorption (mg)	75	mg	O	75	mg	O
Exposure per task / Acute Dose	1,25	mg/kg bw	O	9,036144578	mg/kg bw	O
Exposure per day / Chronic Dose	2,5	mg/kg bw/day	O	18,07228916	mg/kg bw/day	O
**Oral**						
Exposure / Dose	accidental		S	accidental		S
**Intake**						
Exposure per task / Acute Dose	1,25	mg/kg bw	S	9,036144578	mg/kg bw	S
Exposure per day / Chronic Dose	2,5	mg/kg bw/day	O	18,07228916	mg/kg bw/day	O

**Disposal**	Task D: not applicable					

U = Undefined, D = Default, S = Set, O = Output, REM = Remark, not used in calculation

Scenario 20: personal care: head leave-on

Scenario description

				Comments
Name of product	hair gel, make-up, after shave		S	
Physical state product (liquid/solid)	liquid		S	
Density product	1	g/cm^3	D	
Concentration of active substance in product	1	%	S	*Frame Formulation*
Concentration of active substance in product (mg/l)	10000	mg/l	O	
User	Consumer		S	
Bystander	Children		S	
Temperature	20	°C	D	*room temperature*
**Mixing & Loading**	Task A: not applicable			
**Application**	Task B: styling hair with gel			
Number of tasks per year	730	tasks/yr	S	*twice per day*
Duration of task	5	min/task	S	
Quantity of product used per task	5	g/task	S	*hair styling products [TGD (2003)]*
Volume of product used per task	5	ml/task	O	
Quantity of active substance used per task	50	mg/task	O	
Model inhalation exposure	Exposure to vapour / Instantaneous release (limited to vapour pressure)			
Room volume	1	m^3	S	*cloud around user*
Model dermal exposure	Direct dermal contact			
Contact area between product and skin	0,202	m^2	S	*Head (face) + Hands*
Contact amount between product and skin	5	g/task	S	*Whole amount used*
Model oral exposure	accidental			
**Post application phase**	Task C: leave on			
Number of tasks per year	730	tasks/yr	S	*twice per day*
Duration of task	720	min/task	S	*12 hours, ConsExpo: 24 hours*
Quantity of active substance used per task	50	mg/task	O	
Model inhalation exposure	Exposure to vapour / Instantaneous release (limited to vapour pressure)			
Room volume	1	m^3	S	*cloud around user*
Model dermal exposure	Direct dermal contact			
Contact area between product and skin	0	m^2	S	*all product is supposed to be absorbed during application*
Contact area between product and skin children	0,01040125	m^2	S	*50% Hands children*
Model oral exposure	accidental			
**Disposal**	Task D: not applicable			

Summary Results Exposure

	User		Consumer	Bystander		Children
Highest potential exposure acute (all amount used is absorbed)		0,833333333	mg/kg bw		6,024096386	mg/kg bw
Highest potential exposure chronic (all amount used is absorbed)		1,666666667	mg/kg bw/day		12,04819277	mg/kg bw/day
Highest potential concentration in air		50	mg/m^3		50	mg/m^3
Inhalation acute		0,833333333	mg/kg bw		6,024096386	mg/kg bw
Inhalation chronic		1,666666667	mg/kg bw/day		12,04819277	mg/kg bw/day
Dermal acute		0,833333333	mg/kg bw		1,253162651	mg/kg bw
Dermal chronic		1,666666667	mg/kg bw/day		2,506325301	mg/kg bw/day
Oral acute	accidental			accidental		
Oral chronic	accidental			accidental		
Intake acute		0,833333333	mg/kg bw		6,024096386	mg/kg bw
Daily intake chronic		1,666666667	mg/kg bw/day		12,04819277	mg/kg bw/day

U = Undefined, D = Default, S = Set, O = Output, REM = Remark, not used in calculation

Scenario 20: personal care: head leave-on

Results / Output

Mixing & Loading	Task A: not applicable					

Application	Task B: styling hair with gel					
	**Primary exposure**	**User**		**Secondary exposure**	**Bystander**	
**Inhalation**						
Concentration of potential exposure	50	mg/m^3	O	50	mg/m^3	O
Inhalation	0,694444444	mg/min	O	0,201388889	mg/min	O
Absorption per task	2,604166667	mg/task	O	0,755208333	mg/task	O
Exposure per task / Acute Dose	0,043402778	mg/kg bw	O	0,090988956	mg/kg bw	O
Exposure per day / Chronic Dose	0,086805556	mg/kg bw/day	O	0,181977912	mg/kg bw/day	O
**Dermal**						
Contact area between product and skin	0,202	m^2	S			
Quantity of contact (mg)	50	mg	S			
Dermal load (mg/cm^2)	0,024752475	mg/cm^2	O			
Absorption (mg)	50	mg	O			
Exposure per task / Acute Dose	0,833333333	mg/kg bw	O	no		S
Exposure per day / Chronic Dose	1,666666667	mg/kg bw/day	O	no		S
**Oral**						
Exposure / Dose	accidental		S	accidental		S
**Intake**						
Exposure per task / Acute Dose	0,833333333	mg/kg bw	S	0,090988956	mg/kg bw	O
Exposure per day / Chronic Dose	1,666666667	mg/kg bw/day	O	0,181977912	mg/kg bw/day	O

Post application phase	Task C: leave on					
	**Primary exposure**	**User**		**Secondary exposure**	**Bystander**	
**Inhalation**						
Concentration of potential exposure	50	mg/m^3	O	50	mg/m^3	O
Inhalation	0,694444444	mg/min	O	0,201388889	mg/min	O
Absorption per task	50	mg/task	S	50	mg/task	S
Exposure per task / Acute Dose	0,833333333	mg/kg bw	O	6,024096386	mg/kg bw	O
Exposure per day / Chronic Dose	1,666666667	mg/kg bw/day	O	12,04819277	mg/kg bw/day	O
**Dermal**						
Contact area between product and skin				0,01040125	m^2	S
Volume of contact				1,040125	ml	O
Quantity of contact (mg)				10,40125	mg	O
Dermal load (mg/cm^2)				0,1	mg/cm^2	O
Absorption (mg)				10,40125	mg	O
Exposure per task / Acute Dose	no	mg/kg bw	S	1,253162651	mg/kg bw	O
Exposure per day / Chronic Dose	no	mg/kg bw/day	S	2,506325301	mg/kg bw/day	O
**Oral**						
Exposure / Dose	accidental		S	accidental		S
**Intake**						
Exposure per task / Acute Dose	0,833333333	mg/kg bw	O	6,024096386	mg/kg bw	S
Exposure per day / Chronic Dose	1,666666667	mg/kg bw/day	O	12,04819277	mg/kg bw/day	O

Disposal	Task D: not applicable					

Scenario 21: personal care: hands leave-on

Scenario description

				Comments
Name of product	hand cream		S	
Physical state product (liquid/solid)	liquid		S	
Density product	1	g/cm^3	D	
Concentration of active substance in product	1	%	S	*Frame Formulation*
Concentration of active substance in product (mg/l)	10000	mg/l	O	
User	Consumer		S	
Bystander	Children		S	
Temperature	20	°C	D	*room temperature*

Mixing & Loading

Task A: not applicable

Application + Post application phase

Task B: hand disinfection, hand creaming + leave-on

Number of tasks per year	730	tasks/yr	S	*twice per day, ConsExpo: hand cream*
Duration of task	720	min/task	S	*ConsExpo: hand cream 720 min*
Quantity of product used per task	1,7	g/task	S	*ConsExpo: hand cream, 840 mg [TGD (2003)] general purpose*
Volume of product used per task	1,7	ml/task	O	
Quantity of active substance used per task	17	mg/task	O	
Model inhalation exposure	Exposure to vapour / Instantaneous release (limited to vapour pressure)			
Room volume	1	m^3	S	*cloud around user*
Model dermal exposure	Direct dermal contact			
Contact area between product and skin	0,084	m^2	S	*Hands*
Contact amount between product and skin	1,7	g/task	S	*Whole amount used*
Model oral exposure	accidental			

Disposal

Task D: not applicable

Summary Results Exposure

	User		Consumer	Bystander		Children
Highest potential exposure acute (all amount used is absorbed)		0,283333333	mg/kg bw		2,048192771	mg/kg bw
Highest potential exposure chronic (all amount used is absorbed)		0,566666667	mg/kg bw/day		4,096385542	mg/kg bw/day
Highest potential concentration in air		17	mg/m^3		17	mg/m^3
Inhalation acute		0,283333333	mg/kg bw		2,048192771	mg/kg bw
Inhalation chronic		0,566666667	mg/kg bw/day		4,096385542	mg/kg bw/day
Dermal acute		0,283333333	mg/kg bw		0	mg/kg bw
Dermal chronic		0,566666667	mg/kg bw/day		0	mg/kg bw/day
Oral acute	accidental			accidental		
Oral chronic	accidental			accidental		
Intake acute		0,283333333	mg/kg bw		2,048192771	mg/kg bw
Daily intake chronic		0,566666667	mg/kg bw/day		4,096385542	mg/kg bw/day

U = Undefined, D = Default, S = Set, O = Output, REM = Remark, not used in calculation

Scenario 21: personal care: hands leave-on

Results / Output

**Mixing & Loading**	Task A: not applicable					

**Application + Post application phase**	Task B: hand disinfection, hand creaming + leave-on					
	**Primary exposure**	**User**		**Secondary exposure**	**Bystander**	
**Inhalation**						
Concentration of potential exposure	17	mg/m^3	O	17	mg/m^3	O
Inhalation	0,236111111	mg/min	O	0,068472222	mg/min	O
Absorption per task	17	mg/task	S	17	mg/task	S
Exposure per task / Acute Dose	0,283333333	mg/kg bw	O	2,048192771	mg/kg bw	O
Exposure per day / Chronic Dose	0,566666667	mg/kg bw/day	O	4,096385542	mg/kg bw/day	O
**Dermal**						
Contact area between product and skin	0,084	m^2	S			
Quantity of contact (mg)	17	mg	S			
Dermal load (mg/cm^2)	0,020238095	mg/cm^2	O			
Absorption (mg)	17	mg	O			
Exposure per task / Acute Dose	0,283333333	mg/kg bw	O	no		S
Exposure per day / Chronic Dose	0,566666667	mg/kg bw/day	O	no		S
**Oral**						
Exposure / Dose	accidental		S	accidental		S
**Intake**						
Exposure per task / Acute Dose	0,283333333	mg/kg bw	S	2,048192771	mg/kg bw	O
Exposure per day / Chronic Dose	0,566666667	mg/kg bw/day	O	4,096385542	mg/kg bw/day	O

**Disposal**	Task D: not applicable

U = Undefined, D = Default, S = Set, O = Output, REM = Remark, not used in calculation

Scenario 22: personal care: (ready for-use) spray (deo spray, hair spray)

Scenario description

				Comments
Name of product	hair spray		S	
Physical state product (liquid/solid)	liquid		S	
Density product	1	g/cm^3	D	
Concentration of active substance in product	1	%	S	*Frame Formulation*
Concentration of active substance in product (mg/l)	10000	mg/l	O	
User	Consumer		S	
Bystander	Children		S	
Temperature	20	°C	D	*room temperature*

Mixing & Loading

Task A: not applicable

Application

Task B: spraying onto head + deposit time

Number of tasks per year	365	tasks/yr	S	*once per day, ConsExpo: 438*
Duration of task	5	min/task	S	*ConsExpo: hair: 0.24 min spray and 5 min exposure*
Quantity of product used per task	10	g/task	S	*hair spray [TGD (2003)], deo 3.0 g/task*
Volume of product used per task	10	ml/task	O	
Quantity of active substance used per task	100	mg/task	O	
Model inhalation exposure	Exposure to aerosols / Instantaneous release			
Room volume	1	m^3	S	*cloud around user*
Model dermal exposure	Direct dermal contact			
Contact area between product and skin	0,555	m^2	S	*Head (x2) + upper extremities*
Contact amount between product and skin	10	g/task	S	*Whole amount used*
Contact area between product and skin children	0	m^2	S	*no direct contact*
Model oral exposure	accidental			

Post application phase

Task C: leave-on (drying/residence time)

Number of tasks per year	365	tasks/yr	S	*once per day, ConsExpo: 438*
Duration of task	720	min/task	S	*12 hours, ConsExpo 16 hours dermal*
Quantity of active substance used per task	100	mg/task	O	
Model inhalation exposure	Exposure to vapour / Instantaneous release (limited to vapour pressure)			
Room volume	1	m^3	S	*cloud around user*
Model dermal exposure	Direct dermal contact			
Contact area between product and skin	0,555	m^2	S	*Head (x2) + upper extremities*
Contact amount between product and skin adult	0	g/task	S	*all product is supposed to be absorbed during application*
Contact area between product and skin children	0,0208025	m^2	S	*Hands children*
Model oral exposure	accidental			

Disposal

Task D: not applicable

Summary Results Exposure

	User	Consumer		Bystander	Children	
Highest potential exposure acute (all amount used is absorbed)		1,666666667	mg/kg bw		12,04819277	mg/kg bw
Highest potential exposure chronic (all amount used is absorbed)		1,666666667	mg/kg bw/day		12,04819277	mg/kg bw/day
Highest potential concentration in air		100	mg/m^3		100	mg/m^3
Inhalation acute		1,666666667	mg/kg bw		12,04819277	mg/kg bw
Inhalation chronic		1,666666667	mg/kg bw/day		12,04819277	mg/kg bw/day
Dermal acute		1,666666667	mg/kg bw		2,506325301	mg/kg bw
Dermal chronic		1,666666667	mg/kg bw/day		2,506325301	mg/kg bw/day
Oral acute	accidental			accidental		
Oral chronic	accidental			accidental		
Intake acute		1,666666667	mg/kg bw		12,04819277	mg/kg bw
Daily intake chronic		1,666666667	mg/kg bw/day		12,04819277	mg/kg bw/day

U = Undefined, D = Default, S = Set, O = Output, REM = Remark, not used in calculation

Scenario 22: personal care: (ready for-use) spray (deo spray, hair spray)

Results / Output

**Mixing & Loading**	Task A: not applicable					

**Application**	Task B: spraying onto head + deposit time					
	**Primary exposure**	**User**		**Secondary exposure**	**Bystander**	
**Inhalation**						
Concentration of potential exposure	100	mg/m^3	O	100	mg/m^3	O
Inhalation	1,388888889	mg/min	O	0,402777778	mg/min	O
Absorption per task	5,208333333	mg/task	O	1,510416667	mg/task	O
Exposure per task / Acute Dose	0,086805556	mg/kg bw	O	0,181977912	mg/kg bw	O
Exposure per day / Chronic Dose	0,086805556	mg/kg bw/day	O	0,181977912	mg/kg bw/day	O
**Dermal**						
Contact area between product and skin	0,555	m^2	S			
Volume of contact	55,5	ml	O			
Quantity of contact (mg)	100	mg	S			
Dermal load (mg/cm^2)	0,018018018	mg/cm^2	O			
Absorption (mg)	100	mg	O			
Exposure per task / Acute Dose	1,666666667	mg/kg bw	O	no		S
Exposure per day / Chronic Dose	1,666666667	mg/kg bw/day	O	no		S
**Oral**						
Exposure / Dose	accidental		S	accidental		S
**Intake**						
Exposure per task / Acute Dose	1,666666667	mg/kg bw	S	0,181977912	mg/kg bw	O
Exposure per day / Chronic Dose	1,666666667	mg/kg bw/day	O	0,181977912	mg/kg bw/day	O

**Post application phase**	Task C: leave-on (drying/residence time)					
	**Primary exposure**	**User**		**Secondary exposure**	**Bystander**	
**Inhalation**						
Concentration of potential exposure	100	mg/m^3	O	100	mg/m^3	O
Inhalation	1,388888889	mg/min	O	0,402777778	mg/min	O
Absorption per task	100	mg/task	S	100	mg/task	S
Exposure per task / Acute Dose	1,666666667	mg/kg bw	O	12,04819277	mg/kg bw	O
Exposure per day / Chronic Dose	1,666666667	mg/kg bw/day	O	12,04819277	mg/kg bw/day	O
**Dermal**						
Contact area between product and skin	0,555	m^2	S	0,0208025	m^2	S
Volume of contact	0	ml	S	2,08025	ml	O
Quantity of contact (mg)	0	mg	S	20,8025	mg	O
Dermal load (mg/cm^2)	0	mg/cm^2	O	0,1	mg/cm^2	O
Absorption (mg)	0	mg	O	20,8025	mg	O
Exposure per task / Acute Dose	0	mg/kg bw	O	2,506325301	mg/kg bw	O
Exposure per day / Chronic Dose	0	mg/kg bw/day	O	2,506325301	mg/kg bw/day	O
**Oral**						
Exposure / Dose	accidental		S	accidental		S
**Intake**						
Exposure per task / Acute Dose	1,666666667	mg/kg bw	O	12,04819277	mg/kg bw	S
Exposure per day / Chronic Dose	1,666666667	mg/kg bw/day	O	12,04819277	mg/kg bw/day	O

**Disposal**	Task D: not applicable					

U = Undefined, D = Default, S = Set, O = Output, REM = Remark, not used in calculation

Scenario 23: personal care: wet tissues

Scenario description

				Comments
Name of product	wet tissue, fresh-up towel		S	
Physical state product (liquid/solid)	liquid/solid		S	
Concentration of active substance in product	1	g/wipe	S	*Frame Formulation*
User	Consumer		S	
User 2	Children		S	
Temperature	20	°C	D	*room temperature*

Mixing & Loading

Task A: not applicable

Application

Task B: wiping bottom + drying time (leave-on)

Number of tasks per year	720	tasks/yr	S	*twice per day*
Duration of task	720	min/task	S	*application + leave-on*
Quantity of wipes used per task	2	wipes/task	S	
Quantity of active substance used per task	2000	mg/task	O	
Model inhalation exposure	Exposure to vapour / Instantaneous release (limited to vapour pressure)			
Room volume	2,5	m^3	S	*Toilet*
Room volume children	16	m^3	S	*Children Room with diaper changing table*
Model dermal exposure	Direct dermal contact			
Contact area between product and skin	0,084	m^2	S	*Bottom (no data), estimated approx Hands*
Contact area between product and skin children	0,0208025	m^2	S	*Bottom Children (no data), estimated approx. Hands*
Contact amount between active substance and skin	1000	mg/task	S	*50% of whole amount used*
Model oral exposure	accidental			

Post application phase

Task C: not applicable

Disposal

Task D: not applicable

Summary Results Exposure

	User		Consumer	User 2		Children
Highest potential exposure acute (all amount used is absorbed)		33,33333333	mg/kg bw		240,9638554	mg/kg bw
Highest potential exposure chronic (all amount used is absorbed)		65,75342466	mg/kg bw/day		475,3259614	mg/kg bw/day
Highest potential concentration in air		205,1493218	mg/m^3		125	mg/m^3
Inhalation acute		25,64366523	mg/kg bw		32,7560241	mg/kg bw
Inhalation chronic		50,58476429	mg/kg bw/day		64,61462288	mg/kg bw/day
Dermal acute		16,66666667	mg/kg bw		120,4819277	mg/kg bw
Dermal chronic		32,87671233	mg/kg bw/day		237,6629807	mg/kg bw/day
Oral acute	accidental			accidental		
Oral chronic	accidental			accidental		
Intake acute		33,33333333	mg/kg bw		153,2379518	mg/kg bw
Daily intake chronic		65,75342466	mg/kg bw/day		302,2776036	mg/kg bw/day

U = Undefined, D = Default, S = Set, O = Output, REM = Remark, not used in calculation

Scenario 23: personal care: wet tissues

Results / Output

Mixing & Loading	Task A: not applicable					

Application	Task B: wiping bottom + drying time (leave-on)					
	**Primary exposure**	**User**		**Secondary exposure**	**Bystander**	
**Inhalation**						
Concentration of potential exposure	205,1493218	mg/m^3	S	125	mg/m^3	O
Inhalation	2,849296137	mg/min	O	0,503472222	mg/min	O
Absorption per task	1538,619914	mg/task	O	271,875	mg/task	O
Exposure per task / Acute Dose	25,64366523	mg/kg bw	O	32,7560241	mg/kg bw	O
Exposure per day / Chronic Dose	50,58476429	mg/kg bw/day	O	64,61462288	mg/kg bw/day	O
**Dermal**						
Contact area between product and skin	0,084	m^2	S	0,0208025	m^2	S
Quantity of contact (mg)	1000	mg	S	1000	mg	S
Dermal load (mg/cm^2)	1,19047619	mg/cm^2	O	4,80711453	mg/cm^2	O
Absorption (mg)	1000	mg	O	1000	mg	O
Exposure per task / Acute Dose	16,66666667	mg/kg bw	O	120,4819277	mg/kg bw	O
Exposure per day / Chronic Dose	32,87671233	mg/kg bw/day	O	237,6629807	mg/kg bw/day	O
**Oral**						
Exposure / Dose	accidental		S	accidental		S
**Intake**						
Exposure per task / Acute Dose	33,33333333	mg/kg bw	S	153,2379518	mg/kg bw	O
Exposure per day / Chronic Dose	65,75342466	mg/kg bw/day	O	302,2776036	mg/kg bw/day	O

Post application phase	Task C: not applicable

Disposal	Task D: not applicable

U = Undefined, D = Default, S = Set, O = Output, REM = Remark, not used in calculation

Scenario 24: personal care: hands/feet using (ready for-use) spray

Scenario description

				Comments
Name of product	foot spray		S	
Physical state product (liquid/solid)	liquid		S	
Density product	1	g/cm^3	D	
Concentration of active substance in product	1	%	S	*Frame Formulation*
Concentration of active substance in product (mg/l)	10000	mg/l	O	
User	Consumer		S	
Bystander	Children		S	*no direct application*
Temperature	20	°C	D	*room temperature*

Mixing & Loading

Task A: not applicable

Application

Task B: spraying onto feet (or hands) + desposit time of aerosols

Number of tasks per year	90	tasks/yr	S	*[ConsExpo: foot cream-anti fungal (antiperspirant 730 tasks)]*
Duration of task	2	min/task	S	*20 s spraying + rest deposit*
Surface (treated per task)	0,112	m^2/task	S	*Feet*
Quantity of product used per m^2	50	ml/m^2	D	
Quantity of product used per task	5,6	ml/task	O	
Quantity of active substance used per task	56	mg/task	O	
Model inhalation exposure	Exposure to aerosols / Instantaneous release			
Room volume	1	m^3	S	*cloud around user*
Model dermal exposure	Direct dermal contact			
Contact area between product and skin	0,31	m^2	S	*Forearms, Hands, Feet*
Contact area between product and skin children	0	m^2	S	*no direct contact*
Model oral exposure	accidental			

Post application phase

Task C: leave-on (drying/residence time)

Number of tasks per year	90	tasks/yr	S	*[ConsExpo: foot cream-anti fungal (antiperspirant 730 tasks)]*
Duration of task	720	min/task	S	*ConsExpo 12 hours*
Quantity of active substance used per task	56	mg/task	O	
Model inhalation exposure	Exposure to vapour / Instantaneous release (limited to vapour pressure)			
Room volume	1	m^3	0	*cloud around user*
Model dermal exposure	Direct dermal contact			
Contact area between product and skin	0,31	m^2	S	*Forearms, Hands, Feet*
Contact area between product and skin children	0	m^2	S	*no direct contact*
Contact amount between product and skin	0	g/task	S	*all product is supposed to be absorbed during application*
Model oral exposure	accidental			

Disposal

Task D: not applicable

Summary Results Exposure

	User		Consumer	Bystander		Children
Highest potential exposure acute (all amount used is absorbed)		0,933333333	mg/kg bw		6,746987952	mg/kg bw
Highest potential exposure chronic (all amount used is absorbed)		0,230136986	mg/kg bw/day		1,663640865	mg/kg bw/day
Highest potential concentration in air		56	mg/m^3		56	mg/m^3
Inhalation acute		0,019444444	mg/kg bw		0,040763052	mg/kg bw
Inhalation chronic		0,004794521	mg/kg bw/day		0,010051164	mg/kg bw/day
Dermal acute		0,933333333	mg/kg bw		0	mg/kg bw
Dermal chronic		0,230136986	mg/kg bw/day		0	mg/kg bw/day
Oral acute	accidental			accidental		
Oral chronic	accidental			accidental		
Intake acute		0,933333333	mg/kg bw		1,182128514	mg/kg bw
Daily intake chronic		0,230136986	mg/kg bw/day		0,291483743	mg/kg bw/day

U = Undefined, D = Default, S = Set, O = Output, REM = Remark, not used in calculation

Scenario 24: personal care: hands/feet using (ready for-use) spray

Results / Output

**Mixing & Loading**	Task A: not applicable					

**Application**	Task B: spraying onto feet (or hands) + desposit time of aerosols					
	**Primary exposure**	**User**		**Secondary exposure**	**Bystander**	
**Inhalation**						
Concentration of potential exposure	56	mg/m^3	O	56	mg/m^3	O
Inhalation	0,777777778	mg/min	O	0,225555556	mg/min	O
Absorption per task	1,166666667	mg/task	O	0,338333333	mg/task	O
Exposure per task / Acute Dose	0,019444444	mg/kg bw	O	0,040763052	mg/kg bw	O
Exposure per day / Chronic Dose	0,004794521	mg/kg bw/day	O	0,010051164	mg/kg bw/day	O
**Dermal**						
Contact area between product and skin	0,31	m^2	S			
Volume of contact	31	ml	O			
Quantity of contact (mg)	56	mg	S			
Dermal load (mg/cm^2)	0,018064516	mg/cm^2	O			
Absorption (mg)	56	mg	O			
Exposure per task / Acute Dose	0,933333333	mg/kg bw	O	no		S
Exposure per day / Chronic Dose	0,230136986	mg/kg bw/day	O	no		S
**Oral**						
Exposure / Dose	accidental		S	accidental		S
**Intake**						
Exposure per task / Acute Dose	0,933333333	mg/kg bw	S	0,040763052	mg/kg bw	O
Exposure per day / Chronic Dose	0,230136986	mg/kg bw/day	O	0,010051164	mg/kg bw/day	O

**Post application phase**	Task C: leave-on (drying/residence time)					
	**Primary exposure**	**User**		**Secondary exposure**	**Bystander**	
**Inhalation**						
Concentration of potential exposure	56	mg/m^3	O	56	mg/m^3	O
Inhalation	0,777777778	mg/min	O	0,225555556	mg/min	O
Absorption per task	32,66666667	mg/task	O	9,473333333	mg/task	O
Exposure per task / Acute Dose	0,544444444	mg/kg bw	O	1,141365462	mg/kg bw	O
Exposure per day / Chronic Dose	0,134246575	mg/kg bw/day	S	0,28143258	mg/kg bw/day	O
**Dermal**						
Contact area between product and skin	0,31	m^2	S			
Quantity of contact (mg)	0	mg	S			
Dermal load (mg/cm^2)	0	mg/cm^2	O			
Absorption (mg)	0	mg	O			
Exposure per task / Acute Dose	0	mg/kg bw	O	no	mg/kg bw	S
Exposure per day / Chronic Dose	0	mg/kg bw/day	O	no	mg/kg bw/day	S
**Oral**						
Exposure / Dose	accidental		S	accidental		S
**Intake**						
Exposure per task / Acute Dose	0,544444444	mg/kg bw	O	1,141365462	mg/kg bw	O
Exposure per day / Chronic Dose	0,134246575	mg/kg bw/day	O	0,28143258	mg/kg bw/day	O

**Disposal**	Task D: not applicable					

U = Undefined, D = Default, S = Set, O = Output, REM = Remark, not used in calculation

Scenario 25: personal care: toothpaste

Scenario description

				Comments
Name of product	antiseptic tooth paste		S	
Physical state product (liquid/solid)	liquid		S	
Density product	1	g/cm^3	D	
Concentration of active substance in product	1	%	S	*Frame Formulation*
Concentration of active substance in product (mg/l)	10000	mg/l	O	
User	Consumer		S	
User 2	Children		S	
Temperature	20	°C	D	*room temperature*

Mixing & Loading

Task A: not applicable

Application

Task B: brushing the teeth

Number of tasks per year	730	tasks/yr	S	*twice a day [TGD (2003), ConsExpo 4]*
Duration of task	10	min/task	S	
Quantity of product used per task	1,4	g/task	S	*typical [TGD (2003)]*
Quantity of active substance used per task	14	mg/task	O	
Model inhalation exposure	Exposure to vapour / negligible compared with oral exposure			
Model dermal exposure	Direct dermal contact / negligible compared with oral exposure			
Model oral exposure	Direct oral ingestion			
Fraction of product ingested adult	5,714285714	%	S	*amount ingested product: 0.08 g [ConsExpo 4]*
Fraction of product ingested child	37,85714286	%	S	*amount ingested product: 0.53 g [2.5 years, ConsExpo 4]*

Post application phase

Task C: not applicable

Disposal

Task D: not applicable

Summary Results Exposure

	User	Consumer	User 2	Children
Highest potential exposure acute (all amount used is absorbed)	0,233333333	mg/kg bw	1,686746988	mg/kg bw
Highest potential exposure chronic (all amount used is absorbed)	0,466666667	mg/kg bw/day	3,373493976	mg/kg bw/day
Highest potential concentration in air	not available	mg/m^3	not available	mg/m^3
Inhalation acute	negligible	mg/kg bw	negligible	mg/kg bw
Inhalation chronic	negligible	mg/kg bw/day	negligible	mg/kg bw/day
Dermal acute	negligible	mg/kg bw	negligible	mg/kg bw
Dermal chronic	negligible	mg/kg bw/day	negligible	mg/kg bw/day
Oral acute	0,013333333	mg/kg bw	0,638554217	mg/kg bw
Oral chronic	0,026666667	mg/kg bw/day	1,277108434	mg/kg bw/day
Intake acute	0,013333333	mg/kg bw	0,638554217	mg/kg bw
Daily intake chronic	0,026666667	mg/kg bw/day	1,277108434	mg/kg bw/day

U = Undefined, D = Default, S = Set, O = Output, REM = Remark, not used in calculation

Scenario 25: personal care: toothpaste

Results / Output

Mixing & Loading	Task A: not applicable					

Application	Task B: brushing the teeth					
	**Primary exposure**	**User**		**Secondary exposure**	**Bystander**	
**Inhalation**						
Exposure per task / Acute Dose	negligible	mg/kg bw	S	negligible	mg/kg bw	S
Exposure per day / Chronic Dose	negligible	mg/kg bw/day	S	negligible	mg/kg bw/day	S
**Dermal**						
Exposure per task / Acute Dose	negligible	mg/kg bw	S	negligible	mg/kg bw	S
Exposure per day / Chronic Dose	negligible	mg/kg bw/day	S	negligible	mg/kg bw/day	S
**Oral**						
Absorption per task	0,8	mg/task	O	5,3	mg/task	O
Exposure per task / Acute Dose	0,013333333	mg/kg bw	O	0,638554217	mg/kg bw	O
Exposure per day / Chronic Dose	0,026666667	mg/kg bw/day	O	1,277108434	mg/kg bw/day	O
**Intake**						
Exposure per task / Acute Dose	0,013333333	mg/kg bw	O	0,638554217	mg/kg bw	O
Exposure per day / Chronic Dose	0,026666667	mg/kg bw/day	O	1,277108434	mg/kg bw/day	O

Post application phase	Task C: not applicable

Disposal	Task D: not applicable

U = Undefined, D = Default, S = Set, O = Output, REM = Remark, not used in calculation

Scenario 25b: personal care: mouth wash

Scenario description

				Comments
Name of product	antiseptic mouth wash		S	
Physical state product (liquid/solid)	liquid		S	
Density product	1	g/cm^3	D	
Concentration of active substance in product	1	%	S	*Frame Formulation*
Concentration of active substance in product (mg/l)	10000	mg/l	O	
User	Consumer		S	
User 2	Children		S	
Temperature	20	°C	D	*room temperature*

Mixing & Loading

Task A: not applicable

Application

Task B: using mouth wash

Number of tasks per year	1825	tasks/yr	S	*1-5 / day [TGD (2003), ConsExpo 4: 1460 tasks/yr]*
Duration of task	5	min/task	S	
Quantity of product used per task	10	g/task	S	*typical [TGD (2003)]*
Quantity of active substance used per task	100	mg/task	O	
Model inhalation exposure	Exposure to vapour / negligible compared with oral exposure			
Model dermal exposure	Direct dermal contact / negligible compared with oral exposure			
Model oral exposure	Direct oral ingestion			
Fraction of product ingested adult	10	%	S	*amount ingested product: 1 g [ConsExpo 4]*
Fraction of product ingested child	10	%	S	

Post application phase

Task C: not applicable

Disposal

Task D: not applicable

Summary Results Exposure

	User	Consumer	User 2	Children
Highest potential exposure acute (all amount used is absorbed)	1,666666667	mg/kg bw	12,04819277	mg/kg bw
Highest potential exposure chronic (all amount used is absorbed)	8,333333333	mg/kg bw/day	60,24096386	mg/kg bw/day
Highest potential concentration in air	not available	mg/m^3	not available	mg/m^3
Inhalation acute	negligible	mg/kg bw	negligible	mg/kg bw
Inhalation chronic	negligible	mg/kg bw/day	negligible	mg/kg bw/day
Dermal acute	negligible	mg/kg bw	negligible	mg/kg bw
Dermal chronic	negligible	mg/kg bw/day	negligible	mg/kg bw/day
Oral acute	0,166666667	mg/kg bw	1,204819277	mg/kg bw
Oral chronic	0,833333333	mg/kg bw/day	6,024096386	mg/kg bw/day
Intake acute	0,166666667	mg/kg bw	1,204819277	mg/kg bw
Daily intake chronic	0,833333333	mg/kg bw/day	6,024096386	mg/kg bw/day

U = Undefined, D = Default, S = Set, O = Output, REM = Remark, not used in calculation

Scenario 25b: personal care: mouth wash

Results / Output

Mixing & Loading	Task A: not applicable					

Application	Task B: using mouth wash					
	**Primary exposure**	**User**		**Secondary exposure**	**Bystander**	
**Inhalation**						
Exposure per task / Acute Dose	negligible	mg/kg bw	S	negligible	mg/kg bw	S
Exposure per day / Chronic Dose	negligible	mg/kg bw/day	S	negligible	mg/kg bw/day	S
**Dermal**						
Exposure per task / Acute Dose	negligible	mg/kg bw	S	negligible	mg/kg bw	S
Exposure per day / Chronic Dose	negligible	mg/kg bw/day	S	negligible	mg/kg bw/day	S
**Oral**						
Absorption per task	10	mg/task	O	10	mg/task	O
Exposure per task / Acute Dose	0,166666667	mg/kg bw	O	1,204819277	mg/kg bw	O
Exposure per day / Chronic Dose	0,833333333	mg/kg bw/day	O	6,024096386	mg/kg bw/day	O
**Intake**						
Exposure per task / Acute Dose	0,166666667	mg/kg bw	O	1,204819277	mg/kg bw	O
Exposure per day / Chronic Dose	0,833333333	mg/kg bw/day	O	6,024096386	mg/kg bw/day	O

Post application phase	Task C: not applicable

Disposal	Task D: not applicable

Scenario 25c: personal care: lip stick

Scenario description

				Comments
Name of product	preservative in lip stick		S	
Physical state product (liquid/solid)	liquid		S	
Density product	1	g/cm^3	D	
Concentration of active substance in product	1	%	S	*Frame Formulation*
Concentration of active substance in product (mg/l)	10000	mg/l	O	
User	Consumer		S	
User 2	Children		S	
Temperature	20	°C	D	*room temperature*

Mixing & Loading

Task A: not applicable

Application

Task B: using lip stick

Number of tasks per year	2190	tasks/yr	S	*2-6 / day [TGD (2003), ConsExpo 4: 1460 tasks/yr]*
Duration of task	2	min/task	S	
Quantity of product used per task	0,01	g/task	S	*typical [TGD (2003)]*
Quantity of active substance used per task	0,1	mg/task	O	
Model inhalation exposure	Exposure to vapour / negligible compared with oral exposure			
Model dermal exposure	Direct dermal contact / negligible compared with oral exposure			
Model oral exposure	Direct oral ingestion			
Fraction of product ingested adult	100	%	S	*amount ingested product: 0.01 g [ConsExpo 4]*
Fraction of product ingested child	100	%	S	

Post application phase

Task C: not applicable

Disposal

Task D: not applicable

Summary Results Exposure

	User	Consumer	User 2	Children
Highest potential exposure acute (all amount used is absorbed)	0,001666667	mg/kg bw	0,012048193	mg/kg bw
Highest potential exposure chronic (all amount used is absorbed)	0,01	mg/kg bw/day	0,072289157	mg/kg bw/day
Highest potential concentration in air	not available	mg/m^3	not available	mg/m^3
Inhalation acute	negligible	mg/kg bw	negligible	mg/kg bw
Inhalation chronic	negligible	mg/kg bw/day	negligible	mg/kg bw/day
Dermal acute	negligible	mg/kg bw	negligible	mg/kg bw
Dermal chronic	negligible	mg/kg bw/day	negligible	mg/kg bw/day
Oral acute	0,001666667	mg/kg bw	0,012048193	mg/kg bw
Oral chronic	0,01	mg/kg bw/day	0,072289157	mg/kg bw/day
Intake acute	0,001666667	mg/kg bw	0,012048193	mg/kg bw
Daily intake chronic	0,01	mg/kg bw/day	0,072289157	mg/kg bw/day

U = Undefined, D = Default, S = Set, O = Output, REM = Remark, not used in calculation

Scenario 25c: personal care: lip stick

Results / Output

Mixing & Loading	Task A: not applicable					

Application	Task B: using lip stick					
	**Primary exposure**	**User**		**Secondary exposure**	**Bystander**	
**Inhalation**						
Exposure per task / Acute Dose	negligible	mg/kg bw	S	negligible	mg/kg bw	S
Exposure per day / Chronic Dose	negligible	mg/kg bw/day	S	negligible	mg/kg bw/day	S
**Dermal**						
Exposure per task / Acute Dose	negligible	mg/kg bw	S	negligible	mg/kg bw	S
Exposure per day / Chronic Dose	negligible	mg/kg bw/day	S	negligible	mg/kg bw/day	S
**Oral**						
Absorption per task	0,1	mg/task	O	0,1	mg/task	O
Exposure per task / Acute Dose	0,001666667	mg/kg bw	O	0,012048193	mg/kg bw	O
Exposure per day / Chronic Dose	0,01	mg/kg bw/day	O	0,072289157	mg/kg bw/day	O
**Intake**						
Exposure per task / Acute Dose	0,001666667	mg/kg bw	O	0,012048193	mg/kg bw	O
Exposure per day / Chronic Dose	0,01	mg/kg bw/day	O	0,072289157	mg/kg bw/day	O

Post application phase	Task C: not applicable

Disposal	Task D: not applicable

Scenario 26: home improvement: painting, latex paint

Scenario description

				Comments
Name of product	latex paint		S	
Physical state product (liquid/solid)	liquid		S	
Density product	1,5	g/cm^3	S	*SDS Alpina latex paint*
Concentration of active substance in product	1	%	S	*Frame Formulation*
Concentration of active substance in product (mg/l)	15000	mg/l	O	
Packing unit used	10	L	S	*Technical Inf. Alpina latex paint: 2.5, 5 or 10 Liter*
User	Consumer		S	
Bystander	Children		S	
Temperature	20	°C	D	*room temperature*

Mixing & Loading

Task A: not applicable

Application

Task B: painting living room

Number of tasks per year	3	tasks/yr	S	*three days per year*
Duration of task	480	min/task	S	*8 hours*
Surface (treated per task)	71,04	m^2/task	S	*4,8 x 4.8 x 2.5 m, 4 walls and ceiling*
Quantity of product used per m^2	125	ml/m^2	S	*Technical Inf. Alpina latex paint*
Quantity of product used per task	8880	ml/task	O	
Quantity of active substance used per task	133200	mg/task	O	
Model inhalation exposure	Exposure to vapour / Instantaneous release (limited to vapour pressure)			
Room volume	58	m^3	S	*Living room*
Model dermal exposure	Direct dermal contact			
Contact area between product and skin	0,258033333	m^2	S	*Forearms, Hands, 33% Head, 10% Lower legs*
Contact area between product and skin children	0,14601	m^2	S	*Hands, Arms + Head children*
Model oral exposure	accidental			

Post application phase

Task C: drying time

Number of tasks per year	3	tasks/yr	S	*three days per year*
Duration of task	720	min/task	S	*12 hours*
Quantity of active substance used per task	133200	mg/task	O	
Model inhalation exposure	Exposure to vapour / Instantaneous release (limited to vapour pressure)			
Room volume	58	m^3	S	*Living room*
Model dermal exposure	Direct dermal contact			
Contact area between product and skin	0	m^2	S	*no contact*
Contact area between product and skin children	0	m^2	S	*no contact*
Model oral exposure	accidental			

Disposal

Task D: excessive paint is disposed to the main drainage, cleaning the bucket and brushes

Number of tasks per year	3	tasks/yr	S	*three days per year*
Duration of task	15	min/task	S	
Volume of product disposed per task	1,12	L/task	S	
Quantity of active substance disposed per task	16800	mg/task	O	
Model inhalation exposure	Exposure to vapour / Fugacity concept (equilibrium between ready-for-use paint and air, limited to vapour pressure)			
Room volume	1	m^3	S	*cloud around user*
REM Evaporation from mixture, release area product	*0,070685835*	*m^2*	*S*	*bucket diameter 30 cm*
Model dermal exposure	Direct dermal contact			
Contact area between product and skin	0,198	m^2	S	*Hands, Forearms*
Model oral exposure	accidental			

Summary Results Exposure

	User	Consumer	Bystander	Children
Highest potential exposure acute (all amount used is absorbed)	2500	mg/kg bw	18072,28916	mg/kg bw
Highest potential exposure chronic (all amount used is absorbed)	20,54794521	mg/kg bw/day	148,5393629	mg/kg bw/day
Highest potential concentration in air	205,1493218	mg/m^3	205,1493218	mg/m^3
Inhalation acute	42,74745423	mg/kg bw	89,615145	mg/kg bw
Inhalation chronic	0,351348939	mg/kg bw/day	0,736562836	mg/kg bw/day
Dermal acute	11,40083333	mg/kg bw	26,3873494	mg/kg bw
Dermal chronic	0,093705479	mg/kg bw/day	0,216882324	mg/kg bw/day
Oral acute	accidental		accidental	
Oral chronic	accidental		accidental	
Intake acute	54,14828756	mg/kg bw	116,0024944	mg/kg bw
Daily intake chronic	0,445054418	mg/kg bw/day	0,953445159	mg/kg bw/day

U = Undefined, D = Default, S = Set, O = Output, REM = Remark, not used in calculation

Scenario 26: home improvement: painting, latex paint

Results / Output

**Mixing & Loading**	Task A: not applicable					

**Application**	Task B: painting living room					
	**Primary exposure**	**User**		**Secondary exposure**	**Bystander**	
**Inhalation**						
Concentration of potential exposure	205,1493218	mg/m^3	S	205,1493218	mg/m^3	S
Inhalation	2,849296137	mg/min	O	0,82629588	mg/min	O
Absorption per task	1025,746609	mg/task	O	297,4665167	mg/task	O
Exposure per task / Acute Dose	17,09577682	mg/kg bw	O	35,83933936	mg/kg bw	O
Exposure per day / Chronic Dose	0,140513234	mg/kg bw/day	O	0,294569913	mg/kg bw/day	O
**Dermal**						
Contact area between product and skin	0,258033333	m^2	S	0,14601	m^2	S
Volume of contact	25,80333333	ml	O	14,601	ml	O
Quantity of contact	387,05	mg	O	219,015	mg	O
Dermal load (mg/cm^2)	0,15	mg/cm^2	O	0,15	mg/cm^2	O
Absorption (mg)	387,05	mg	O	219,015	mg	O
Exposure per task / Acute Dose	6,450833333	mg/kg bw	O	26,3873494	mg/kg bw	O
Exposure per day / Chronic Dose	0,053020548	mg/kg bw/day	O	0,216882324	mg/kg bw/day	O
**Oral**						
Exposure / Dose	accidental		S	accidental		S
**Intake**						
Exposure per task / Acute Dose	23,54661015	mg/kg bw	O	62,22668875	mg/kg bw	O
Exposure per day / Chronic Dose	0,193533782	mg/kg bw/day	O	0,511452236	mg/kg bw/day	O

**Post application phase**	Task C: drying time					
	**Primary exposure**	**User**		**Secondary exposure**	**Bystander**	
**Inhalation**						
Concentration of potential exposure	205,1493218	mg/m^3	S	205,1493218	mg/m^3	S
Inhalation	2,849296137	mg/min	O	0,82629588	mg/min	O
Absorption per task	1538,619914	mg/task	O	446,199775	mg/task	O
Exposure per task / Acute Dose	25,64366523	mg/kg bw	O	53,75900903	mg/kg bw	O
Exposure per day / Chronic Dose	0,210769851	mg/kg bw/day	O	0,441854869	mg/kg bw/day	O
**Dermal**						
Exposure per task / Acute Dose	no	mg/kg bw	S	no	mg/kg bw	S
Exposure per day / Chronic Dose	no	mg/kg bw/day	S	no	mg/kg bw/day	S
**Oral**						
Exposure / Dose	accidental		S	accidental		S
**Intake**						
Exposure per task / Acute Dose	25,64366523	mg/kg bw	O	53,75900903	mg/kg bw	O
Exposure per day / Chronic Dose	0,210769851	mg/kg bw/day	O	0,441854869	mg/kg bw/day	O

**Disposal**	Task D: excessive paint is disposed to the main drainage, cleaning the bucket and brushes					
	**Primary exposure**	**User**		**Secondary exposure**	**Bystander**	
**Inhalation**						
Concentration of potential exposure	3,076676276	mg/m^3	O	3,076676276	mg/m^3	O
Inhalation	0,042731615	mg/min	O	0,012392168	mg/min	O
Absorption per task	0,480730668	mg/task	O	0,139411894	mg/task	O
Exposure per task / Acute Dose	0,008012178	mg/kg bw	O	0,016796614	mg/kg bw	O
Exposure per day / Chronic Dose	6,58535E-05	mg/kg bw/day	O	0,000138054	mg/kg bw/day	O
**Dermal**						
Contact area between product and skin	0,198	m^2	S			
Volume of contact	19,8	ml	O			
Quantity of contact	297	mg	O			
Dermal load (mg/cm^2)	0,15	mg/cm^2	O			
Absorption (mg)	297	mg	O			
Exposure per task / Acute Dose	4,95	mg/kg bw	O	no		S
Exposure per day / Chronic Dose	0,040684932	mg/kg bw/day	O	no		S
**Oral**						
Exposure / Dose	accidental		S	accidental		S
**Intake**						
Exposure per task / Acute Dose	4,958012178	mg/kg bw	O	0,016796614	mg/kg bw	O
Exposure per day / Chronic Dose	0,040750785	mg/kg bw/day	O	0,000138054	mg/kg bw/day	O

U = Undefined, D = Default, S = Set, O = Output, REM = Remark, not used in calculation

Scenario 26b: home improvement: painting, latex paint + anti mould additive

Scenario description

				Comments
Name of product	anti mould additive		S	
Physical state product (liquid/solid)	liquid		S	
Density product	1	g/cm^3	D	
Concentration of active substance in product (concentrate)	1	%	S	*Frame Formulation*
Concentration of active substance in product (concentrate) (mg/l)	10000	mg/l	O	
User	Consumer		S	
Bystander	Children		S	
Temperature	20	°C	D	*room temperature*

Mixing & Loading

	Task A: simple mixing the additive with the paint			
Number of tasks per year	3	tasks/yr	S	*three days per year*
Duration of task	1,333333333	min/task	S	*80 sec. [mixing spray, TNsG 2002 p. 252]*
Volume of product used per task	250	ml/task	S	*25 ml/l paint, Frame formulation*
Quantity of water used per task	10	L/task	S	*bucket of latex paint,Tech. Inf. Alpina latex paint: 2.5, 5 or 10 L*
Quantity of product used per task	250	g/task	O	
Quantity of active substance used per task	2500	mg/task	S	
Concentration of active substance in ready-for-use paint	250	mg/l	O	
Model inhalation exposure	Exposure to vapour / Fugacity concept (equilibrium between ready-for-use paint and air, limited to vapour pressure)			
Room volume	1	m^3	S	*cloud around user*
REM Evaporation from mixture, release area concentrate	*0,002*	*m^2*	*S*	*bottle diameter 5 cm [TNsG 2002 p. 252]*
REM Evaporation from mixture, release area ready-for-use paint	*0,070685835*	*m^2*	*S*	*bucket diameter 30 cm*
Model dermal exposure	Direct dermal contact			
Contact area between concentrate and skin	0,0168	m^2	S	*Fingertips*
Model oral exposure	accidental			

Application

	Task B: painting living room			
Number of tasks per year	3	tasks/yr	S	*three days per year*
Duration of task	480	min/task	S	*8 hours*
Surface (treated per task)	71,04	m^2/task	S	*4,8 x 4.8 x 2.5 m, 4 walls and ceiling*
Quantity of ready-for-use paint used per m^2	125	ml/m^2	S	*Technical Inf. Alpina latex paint*
Quantity of ready-for-use paint used per task	8880	ml/task	O	
Concentration of active substance in ready-for-use paint	250	mg/l	O	
Quantity of active substance used per task	2220	mg/task	O	
Model inhalation exposure	Exposure to vapour / Instantaneous release (limited to vapour pressure)			
Room volume	58	m^3	S	*Living room*
Model dermal exposure	Direct dermal contact			
Contact area between ready-for-use paint and skin	0,258033333	m^2	S	*Forearms, Hands, 33% Head, 10% Lower legs*
Contact area between ready-for-use paint and skin children	0,14601	m^2	S	*Hands, Arms + Head children*
Model oral exposure	accidental			

Post application phase

	Task C: drying time			
Number of tasks per year	3	tasks/yr	S	*three days per year*
Duration of task	720	min/task	S	*12 hours*
Concentration of active substance in ready-for-use paint	250	mg/l	O	
Quantity of active substance used per task	2220	mg/task	O	
Model inhalation exposure	Exposure to vapour / Instantaneous release (limited to vapour pressure)			
Room volume	58	m^3	S	*Living room*
Model dermal exposure	Direct dermal contact			
Contact area between ready-for-use paint and skin	0	m^2	S	*no contact*
Contact area between ready-for-use paint and skin children	0	m^2	S	*no contact*
Model oral exposure	accidental			

Disposal

	Task D: excessive ready-for-use solution is disposed to the main drainage, cleaning the bucket and brushes			
Number of tasks per year	3	tasks/yr	S	*three days per year*
Duration of task	15	min/task	S	
Volume of ready-for-use paint disposed per task	1,12	L/task	S	
Concentration of active substance in ready-for-use paint	250	mg/l	O	
Quantity of active substance disposed per task	280	mg/task	O	
Model inhalation exposure	Exposure to vapour / Fugacity concept (equilibrium between ready-for-use paint and air, limited to vapour pressure)			
Room volume	1	m^3	S	*cloud around user*
REM Evaporation from mixture, release area ready-for-use paint	*0,070685835*	*m^2*	*S*	*bucket diameter 30 cm*
Model dermal exposure	Direct dermal contact			
Contact area between ready-for-use paint and skin	0,198	m^2	S	*Hands, Forearms*
Model oral exposure	accidental			

Summary Results Exposure

	User	Consumer		Bystander	Children	
Highest potential exposure acute (all amount used is absorbed)		41,66666667	mg/kg bw		301,2048193	mg/kg bw
Highest potential exposure chronic (all amount used is absorbed)		0,342465753	mg/kg bw/day		2,475656049	mg/kg bw/day
Highest potential concentration in air		38,27586207	mg/m^3		38,27586207	mg/m^3
Inhalation acute		7,974283339	mg/kg bw		16,7171723	mg/kg bw
Inhalation chronic		0,065542055	mg/kg bw/day		0,137401416	mg/kg bw/day
Dermal acute		0,470013889	mg/kg bw		0,439789157	mg/kg bw
Dermal chronic		0,003863128	mg/kg bw/day		0,003614705	mg/kg bw/day
Oral acute	accidental			accidental		
Oral chronic	accidental			accidental		
Intake acute		8,444297228	mg/kg bw		17,15696146	mg/kg bw
Daily intake chronic		0,069405183	mg/kg bw/day		0,141016122	mg/kg bw/day

U = Undefined, D = Default, S = Set, O = Output, REM = Remark, not used in calculation

Scenario 26b: home improvement: painting, latex paint + anti mould additive

Results / Output

Mixing & Loading	Task A: simple mixing the additive with the paint					
	**Primary exposure**	**User**		**Secondary exposure**	**Bystander**	
**Inhalation**						
Concentration of potential exposure	0,051286278	mg/m^3	O	0,051286278	mg/m^3	O
Inhalation	0,000712309	mg/min	O	0,00020657	mg/min	O
Absorption per task	0,000712309	mg/task	O	0,00020657	mg/task	O
Exposure per task / Acute Dose	1,18718E-05	mg/kg bw	O	2,48879E-05	mg/kg bw	O
Exposure per day / Chronic Dose	9,75766E-08	mg/kg bw/day	O	2,04558E-07	mg/kg bw/day	O
**Dermal**						
Contact area between product (concentrate) and skin	0,0168	m^2	S			
Volume of contact	1,68	ml	O			
Quantity of contact	16,8	mg	O			
Dermal load (mg/cm^2)	0,1	mg/cm^2	O			
Absorption (mg)	16,8	mg	O			
Exposure per task / Acute Dose	0,28	mg/kg bw	O	no	mg/kg bw	S
Exposure per day / Chronic Dose	0,00230137	mg/kg bw/day	O	no	mg/kg bw/day	S
**Oral**						
Exposure / Dose	accidental		S	accidental		S
**Intake**						
Exposure per task / Acute Dose	0,280011872	mg/kg bw	O	2,48879E-05	mg/kg bw	O
Exposure per day / Chronic Dose	0,002301467	mg/kg bw/day	O	2,04558E-07	mg/kg bw/day	O

Application	Task B: painting living room					
	**Primary exposure**	**User**		**Secondary exposure**	**Bystander**	
**Inhalation**						
Concentration of potential exposure	38,27586207	mg/m^3	O	38,27586207	mg/m^3	O
Inhalation	0,531609195	mg/min	O	0,154166667	mg/min	O
Absorption per task	191,3793103	mg/task	O	55,5	mg/task	O
Exposure per task / Acute Dose	3,189655172	mg/kg bw	O	6,686746988	mg/kg bw	O
Exposure per day / Chronic Dose	0,026216344	mg/kg bw/day	O	0,054959564	mg/kg bw/day	O
**Dermal**						
Contact area between ready-for-use paint and skin	0,258033333	m^2	S	0,14601	m^2	S
Volume of contact	25,80333333	ml	O	14,601	ml	O
Quantity of contact	6,450833333	mg	O	3,65025	mg	O
Dermal load (mg/cm^2)	0,0025	mg/cm^2	O	0,0025	mg/cm^2	O
Absorption (mg)	6,450833333	mg	O	3,65025	mg	O
Exposure per task / Acute Dose	0,107513889	mg/kg bw	O	0,439789157	mg/kg bw	O
Exposure per day / Chronic Dose	0,000883676	mg/kg bw/day	O	0,003614705	mg/kg bw/day	O
**Oral**						
Exposure / Dose	accidental		S	accidental		S
**Intake**						
Exposure per task / Acute Dose	3,297169061	mg/kg bw	O	7,126536145	mg/kg bw	O
Exposure per day / Chronic Dose	0,02710002	mg/kg bw/day	O	0,05857427	mg/kg bw/day	O

Post application phase	Task C: drying time					
	**Primary exposure**	**User**		**Secondary exposure**	**Bystander**	
**Inhalation**						
Concentration of potential exposure	38,27586207	mg/m^3	O	38,27586207	mg/m^3	O
Inhalation	0,531609195	mg/min	O	0,154166667	mg/min	O
Absorption per task	287,0689655	mg/task	O	83,25	mg/task	O
Exposure per task / Acute Dose	4,784482759	mg/kg bw	O	10,03012048	mg/kg bw	O
Exposure per day / Chronic Dose	0,039324516	mg/kg bw/day	O	0,082439346	mg/kg bw/day	O
**Dermal**						
Exposure per task / Acute Dose	no	mg/kg bw	S	no	mg/kg bw	S
Exposure per day / Chronic Dose	no	mg/kg bw/day	S	no	mg/kg bw/day	S
**Oral**						
Exposure / Dose	accidental		S	accidental		S
**Intake**						
Exposure per task / Acute Dose	4,784482759	mg/kg bw	O	10,03012048	mg/kg bw	O
Exposure per day / Chronic Dose	0,039324516	mg/kg bw/day	O	0,082439346	mg/kg bw/day	O

Disposal	Task D: excessive ready-for-use solution is disposed to the main drainage, cleaning the bucket and brushes					
	**Primary exposure**	**User**		**Secondary exposure**	**Bystander**	
**Inhalation**						
Concentration of potential exposure	0,051277938	mg/m^3	O	0,051277938	mg/m^3	O
Inhalation	0,000712194	mg/min	O	0,000206536	mg/min	O
Absorption per task	0,008012178	mg/task	O	0,002323532	mg/task	O
Exposure per task / Acute Dose	0,000133536	mg/kg bw	O	0,000279944	mg/kg bw	O
Exposure per day / Chronic Dose	1,09756E-06	mg/kg bw/day	O	2,30091E-06	mg/kg bw/day	O
**Dermal**						
Contact area between ready-for-use paint and skin	0,198	m^2	S			
Volume of contact	19,8	ml	O			
Quantity of contact	4,95	mg	O			
Dermal load (mg/cm^2)	0,0025	mg/cm^2	O			
Absorption (mg)	4,95	mg	O			
Exposure per task / Acute Dose	0,0825	mg/kg bw	O	no		S
Exposure per day / Chronic Dose	0,000678082	mg/kg bw/day	O	no		S
**Oral**						
Exposure / Dose	accidental		S	accidental		S
**Intake**						
Exposure per task / Acute Dose	0,082633536	mg/kg bw	O	0,000279944	mg/kg bw	O
Exposure per day / Chronic Dose	0,00067918	mg/kg bw/day	O	2,30091E-06	mg/kg bw/day	O

Scenario 27: home improvement: varnish (water-based)

Scenario description

				Comments
Name of product	dispersion varnish		S	
Physical state product (liquid/solid)	liquid		S	
Density product	1,2	g/cm^3	S	*Technical Information Brillux Lacryl Lack*
Concentration of active substance in product	1	%	S	*Frame Formulation*
Concentration of active substance in product (mg/l)	12000	mg/l	O	
Packing unit used	2,5	L	S	*Tech. Inf. Brillux Lacryl Lack 375 ml, 750 ml, 2.5 L*
User	Consumer		S	
Bystander	Children		S	
Temperature	20	°C	D	*room temperature*

Mixing & Loading

Task A: not applicable

Application

Task B: varnishing a radiator or window frame

Number of tasks per year	3	tasks/yr	S	*three days per year*
Duration of task	480	min/task	S	*8 hours*
Surface (treated per task)	19,2	m^2/task	S	*radiator (800 mm x 2000 mm) x 2 (front and back) x 6/day*
Quantity of product used per m^2	130	ml/m^2	S	*Technical Information Brillux Lacryl Lack ca. 110- 130 ml/m^2*
Quantity of product used per task	2496	ml/task	O	
Quantity of active substance used per task	29952	mg/task	O	
Model inhalation exposure	Exposure to vapour / Instantaneous release (limited to vapour pressure)			
Room volume	58	m^3	S	*Living room*
Model dermal exposure	Direct dermal contact			
Contact area between product and skin	0,198	m^2	S	*Forearms, Hands*
Contact area between product and skin children	0,0208025	m^2	S	*Hands children*
Model oral exposure	accidental			

Post application phase

Task C: drying time

Number of tasks per year	3	tasks/yr	S	*three days per year*
Duration of task	720	min/task	S	*12 hours*
Quantity of active substance used per task	29952	mg/task	O	
Model inhalation exposure	Exposure to vapour / Instantaneous release (limited to vapour pressure)			
Room volume	58	m^3	S	*Living room*
Model dermal exposure	Direct dermal contact			
Contact area between product and skin	0	m^2	S	*no contact*
Contact area between product and skin children	0	m^2	S	*no contact*
Model oral exposure	accidental			

Disposal

Task D: excessive ready-for-use varnish is disposed to the main drainage, cleaning the bucket and brushes

Number of tasks per year	3	tasks/yr	S	*three days per year*
Duration of task	15	min/task	S	
Volume of product disposed per task	0,004	L/task	S	
Quantity of active substance disposed per task	48	mg/task	O	
Model inhalation exposure	Exposure to vapour / Fugacity concept (equilibrium between ready-for-use paint and air, limited to vapour pressure)			
Room volume	1	m^3	S	*cloud around user*
Evaporation from mixture, release area product	*0,070685835*	*m^2*	*S*	*bucket diameter 30 cm*
Model dermal exposure	Direct dermal contact			
Contact area between product and skin	0,198	m^2	S	*Hands, Forearms*
Model oral exposure	accidental			

Summary Results Exposure

	User	Consumer		Bystander	Children	
Highest potential exposure acute (all amount used is absorbed)		500	mg/kg bw		3614,457831	mg/kg bw
Highest potential exposure chronic (all amount used is absorbed)		4,109589041	mg/kg bw/day		29,70787259	mg/kg bw/day
Highest potential concentration in air		205,1493218	mg/m^3		205,1493218	mg/m^3
Inhalation acute		42,74554021	mg/kg bw		89,61113248	mg/kg bw
Inhalation chronic		0,351333207	mg/kg bw/day		0,736529856	mg/kg bw/day
Dermal acute		4,76	mg/kg bw		3,007590361	mg/kg bw
Dermal chronic		0,039123288	mg/kg bw/day		0,024719921	mg/kg bw/day
Oral acute	accidental			accidental		
Oral chronic	accidental			accidental		
Intake acute		47,49944205	mg/kg bw		92,61872284	mg/kg bw
Daily intake chronic		0,390406373	mg/kg bw/day		0,761249777	mg/kg bw/day

U = Undefined, D = Default, S = Set, O = Output, REM = Remark, not used in calculation

Scenario 27: home improvement: varnish (water-based)

Results / Output

Mixing & Loading	Task A: not applicable					

Application	Task B: varnishing a radiator or window frame					
	Primary exposure	User		Secondary exposure	Bystander	
**Inhalation**						
Concentration of potential exposure	205,1493218	mg/m^3	S	205,1493218	mg/m^3	S
Inhalation	2,849296137	mg/min	O	0,82629588	mg/min	O
Absorption per task	1025,746609	mg/task	O	297,4665167	mg/task	O
Exposure per task / Acute Dose	17,09577682	mg/kg bw	O	35,83933936	mg/kg bw	O
Exposure per day / Chronic Dose	0,140513234	mg/kg bw/day	O	0,294569913	mg/kg bw/day	O
**Dermal**						
Contact area between product and skin	0,198	m^2	S	0,0208025	m^2	S
Volume of contact	19,8	ml	O	2,08025	ml	O
Quantity of contact	237,6	mg	O	24,963	mg	O
Dermal load (mg/cm^2)	0,12	mg/cm^2	O	0,12	mg/cm^2	O
Absorption (mg)	237,6	mg	O	24,963	mg	O
Exposure per task / Acute Dose	3,96	mg/kg bw	O	3,007590361	mg/kg bw	O
Exposure per day / Chronic Dose	0,032547945	mg/kg bw/day	O	0,024719921	mg/kg bw/day	O
**Oral**						
Exposure / Dose	accidental		S	accidental		S
**Intake**						
Exposure per task / Acute Dose	21,05577682	mg/kg bw	O	38,84692972	mg/kg bw	O
Exposure per day / Chronic Dose	0,173061179	mg/kg bw/day	O	0,319289833	mg/kg bw/day	O

Post application phase	Task C: drying time					
	Primary exposure	User		Secondary exposure	Bystander	
**Inhalation**						
Concentration of potential exposure	205,1493218	mg/m^3	S	205,1493218	mg/m^3	S
Inhalation	2,849296137	mg/min	O	0,82629588	mg/min	O
Absorption per task	1538,619914	mg/task	O	446,199775	mg/task	O
Exposure per task / Acute Dose	25,64366523	mg/kg bw	O	53,75900903	mg/kg bw	O
Exposure per day / Chronic Dose	0,210769851	mg/kg bw/day	O	0,441854869	mg/kg bw/day	O
**Dermal**						
Exposure per task / Acute Dose	no	mg/kg bw	S	no	mg/kg bw	S
Exposure per day / Chronic Dose	no	mg/kg bw/day	S	no	mg/kg bw/day	S
**Oral**						
Exposure / Dose	accidental		S	accidental		S
**Intake**						
Exposure per task / Acute Dose	25,64366523	mg/kg bw	O	53,75900903	mg/kg bw	O
Exposure per day / Chronic Dose	0,210769851	mg/kg bw/day	O	0,441854869	mg/kg bw/day	O

Disposal	Task D: excessive ready-for-use varnish is disposed to the main drainage, cleaning the bucket and brushes					
	Primary exposure	User		Secondary exposure	Bystander	
**Inhalation**						
Concentration of potential exposure	2,341692695	mg/m^3	O	2,341692695	mg/m^3	O
Inhalation	0,03252351	mg/min	O	0,009431818	mg/min	O
Absorption per task	0,365889484	mg/task	O	0,10610795	mg/task	O
Exposure per task / Acute Dose	0,006098158	mg/kg bw	O	0,01278409	mg/kg bw	O
Exposure per day / Chronic Dose	5,01218E-05	mg/kg bw/day	O	0,000105075	mg/kg bw/day	O
**Dermal**						
Contact area between product and skin	0,198	m^2	S			
Volume of contact	19,8	ml	O			
Quantity of contact	48	mg	S			
Dermal load (mg/cm^2)	0,024242424	mg/cm^2	O			
Absorption (mg)	48	mg	O			
Exposure per task / Acute Dose	0,8	mg/kg bw	O	no		S
Exposure per day / Chronic Dose	0,006575342	mg/kg bw/day	O	no		S
**Oral**						
Exposure / Dose	accidental		S	accidental		S
**Intake**						
Exposure per task / Acute Dose	0,8	mg/kg bw	S	0,01278409	mg/kg bw	O
Exposure per day / Chronic Dose	0,006575342	mg/kg bw/day	O	0,000105075	mg/kg bw/day	O

U = Undefined, D = Default, S = Set, O = Output, REM = Remark, not used in calculation

Scenario 28: home improvement: wallpaper paste anti mould additive

Scenario description

				Comments
Name of product	anti-mould additive for wallpaper paste		S	
Physical state product (liquid/solid)	liquid		S	
Density product	1	g/cm^3	D	
Concentration of active substance in product	1	%	S	*Frame Formulation*
Concentration of active substance in product (mg/l)	10000	mg/l	O	
User	Consumer		S	
Bystander	Children		S	
Temperature	20	°C	D	*room temperature*

Mixing & Loading

	Task A: simple mixing the powder and the additive with water			
Number of tasks per year	3	tasks/yr	S	*three days per year*
Duration of task	1,333333333	min/task	S	*80 sec. [mixing spray TNsG 2002 p. 252]*
Quantity of product used per task	750	g/task	S	*for 3 packages wallpaper paste (3x200 g)*
Quantity of water used per task	12	L/task	S	*Technical Inf. 3 packages sufficient for ca. 78 qm*
Quantity of active substance used per task	7500	mg/task	S	
Concentration of active substance in ready-for-use paste	625	mg/l	O	
Model inhalation exposure	Exposure to vapour / Fugacity concept (equilibrium between ready-for-use paste and air, limited to vapour pressure)			
Room volume	1	m^3	S	*cloud around user*
REM Evaporation from mixture, release area powder	*??*	*m^2*	*U*	
REM Evaporation from mixture, release area ready-for-use paste	*0,070685835*	*m^2*	*S*	*bucket diameter 30 cm*
Model dermal exposure	Direct dermal contact			
Contact area between product and skin	0,0168	m^2	S	*Fingertips*
Model oral exposure	accidental			

Application

	Task B: wallpaper living room			
Number of tasks per year	3	tasks/yr	S	*three days per year*
Duration of task	480	min/task	S	*8 hours*
Surface (treated per task)	71,04	m^2/task	S	*4,8 x 4.8 x 2.5 m, 4 walls and ceiling*
Quantity of ready-for-use paste used per m^2	153	ml/m^2	S	
Quantity of ready-for-use paste used per task	10869,12	ml/task	O	
Concentration of active substance in ready-for-use paste	625	mg/l	O	
Quantity of active substance used per task	6793,2	mg/task	O	
Model inhalation exposure	Exposure to vapour / Instantaneous release (limited to vapour pressure)			
Room volume	58	m^3	S	*Living room*
Model dermal exposure	Direct dermal contact			
Contact area between ready-for-use paste and skin	0,258033333	m^2	S	*Forearms, Hands, 33% Head, 10% Lower legs*
Contact area between ready-for-use paste and skin children	0,14601	m^2	S	*Hands, Arms + Head children*
Model oral exposure	accidental			

Post application phase

	Task C: drying time			
Number of tasks per year	3	tasks/yr	S	*three days per year*
Duration of task	720	min/task	S	*12 hours*
Concentration of active substance in ready-for-use paste	625	mg/l	O	
Quantity of active substance used per task	6793,2	mg/task	O	
Model inhalation exposure	Exposure to vapour / Instantaneous release (limited to vapour pressure)			
Room volume	58	m^3	S	*Living room*
Model dermal exposure	Direct dermal contact			
Contact area between ready-for-use paste and skin	0	m^2	S	*no contact*
Contact area between ready-for-use paste and skin children	0	m^2	S	*no contact*
Model oral exposure	accidental			

Disposal

	Task D: excessive ready-for-use paste is disposed to the main drainage, cleaning the bucket and brushes			
Number of tasks per year	3	tasks/yr	S	*three days per year*
Duration of task	15	min/task	S	
Volume of ready-for-use paste disposed per task	1,13088	L/task	S	
Concentration of active substance in ready-for-use paste	625	mg/l	O	
Quantity of active substance disposed per task	706,8	mg/task	O	
Model inhalation exposure	Exposure to vapour / Fugacity concept (equilibrium between ready-for-use paste and air, limited to vapour pressure)			
Room volume	1	m^3	S	*cloud around user*
REM Evaporation from mixture, release area ready-for-use paste	*0,070685835*	*m^2*	*S*	*bucket diameter 30 cm*
Model dermal exposure	Direct dermal contact			
Contact area between ready-for-use paste and skin	0,198	m^2	S	*Hands, Forearms*
Model oral exposure	accidental			

Summary Results Exposure

	User	Consumer		Bystander	Children	
Highest potential exposure acute (all amount used is absorbed)		125	mg/kg bw		903,6144578	mg/kg bw
Highest potential exposure chronic (all amount used is absorbed)		1,02739726	mg/kg bw/day		7,426968147	mg/kg bw/day
Highest potential concentration in air		117,1241379	mg/m^3		117,1241379	mg/m^3
Inhalation acute		24,40122559	mg/kg bw		51,15437654	mg/kg bw
Inhalation chronic		0,200558019	mg/kg bw/day		0,42044693	mg/kg bw/day
Dermal acute		0,755034722	mg/kg bw		1,099472892	mg/kg bw
Dermal chronic		0,006205765	mg/kg bw/day		0,009036763	mg/kg bw/day
Oral acute	accidental			accidental		
Oral chronic	accidental			accidental		
Intake acute		25,15626031	mg/kg bw		52,25384943	mg/kg bw
Daily intake chronic		0,206763783	mg/kg bw/day		0,429483694	mg/kg bw/day

Scenario 28: home improvement: wallpaper paste anti mould additive

Results / Output

**Mixing & Loading**	Task A: simple mixing the powder and the additive with water					
	**Primary exposure**	**User**		**Secondary exposure**	**Bystander**	
**Inhalation**						
Concentration of potential exposure	0,128216134	mg/m^3	O	0,128216134	mg/m^3	O
Inhalation	0,00178078	mg/min	O	0,000516426	mg/min	O
Absorption per task	0,00178078	mg/task	O	0,000516426	mg/task	O
Exposure per task / Acute Dose	2,96797E-05	mg/kg bw	O	6,222E-05	mg/kg bw	O
Exposure per day / Chronic Dose	2,43942E-07	mg/kg bw/day	O	5,11397E-07	mg/kg bw/day	O
**Dermal**						
Contact area between product (powder) and skin	0,0168	m^2	S			
Volume of contact	1,68	ml	O			
Quantity of contact (mg)	16,8	mg	O			
Dermal load (mg/cm^2)	0,1	mg/cm^2	O			
Absorption (mg)	16,8	mg	O			
Exposure per task / Acute Dose	0,28	mg/kg bw	O	no	mg/kg bw	S
Exposure per day / Chronic Dose	0,00230137	mg/kg bw/day	O	no	mg/kg bw/day	S
**Oral**						
Exposure / Dose	accidental		S	accidental		S
**Intake**						
Exposure per task / Acute Dose	0,28002968	mg/kg bw	O	6,222E-05	mg/kg bw	O
Exposure per day / Chronic Dose	0,002301614	mg/kg bw/day	O	5,11397E-07	mg/kg bw/day	O

**Application**	Task B: wallpaper living room					
	**Primary exposure**	**User**		**Secondary exposure**	**Bystander**	
**Inhalation**						
Concentration of potential exposure	117,1241379	mg/m^3	O	117,1241379	mg/m^3	O
Inhalation	1,626724138	mg/min	O	0,47175	mg/min	O
Absorption per task	585,6206897	mg/task	O	169,83	mg/task	O
Exposure per task / Acute Dose	9,760344828	mg/kg bw	O	20,46144578	mg/kg bw	O
Exposure per day / Chronic Dose	0,080222012	mg/kg bw/day	O	0,168176267	mg/kg bw/day	O
**Dermal**						
Contact area between ready-for-use paste and skin	0,258033333	m^2	S	0,14601	m^2	S
Volume of contact	25,80333333	ml	O	14,601	ml	O
Quantity of contact	16,12708333	mg	O	9,125625	mg	O
Dermal load (mg/cm^2)	0,00625	mg/cm^2	O	0,00625	mg/cm^2	O
Absorption (mg)	16,12708333	mg	O	9,125625	mg	O
Exposure per task / Acute Dose	0,268784722	mg/kg bw	O	1,099472892	mg/kg bw	O
Exposure per day / Chronic Dose	0,002209189	mg/kg bw/day	O	0,009036763	mg/kg bw/day	O
**Oral**						
Exposure / Dose	accidental		S	accidental		S
**Intake**						
Exposure per task / Acute Dose	10,02912955	mg/kg bw	O	21,56091867	mg/kg bw	O
Exposure per day / Chronic Dose	0,082431202	mg/kg bw/day	O	0,17721303	mg/kg bw/day	O

**Post application phase**	Task C: drying time					
	**Primary exposure**	**User**		**Secondary exposure**	**Bystander**	
**Inhalation**						
Concentration of potential exposure	117,1241379	mg/m^3	O	117,1241379	mg/m^3	O
Inhalation	1,626724138	mg/min	O	0,47175	mg/min	O
Absorption per task	878,4310345	mg/task	O	254,745	mg/task	O
Exposure per task / Acute Dose	14,64051724	mg/kg bw	O	30,69216867	mg/kg bw	O
Exposure per day / Chronic Dose	0,120333018	mg/kg bw/day	O	0,2522644	mg/kg bw/day	O
**Dermal**						
Exposure per task / Acute Dose	no	mg/kg bw	S	no	mg/kg bw	S
Exposure per day / Chronic Dose	no	mg/kg bw/day	S	no	mg/kg bw/day	S
**Oral**						
Exposure / Dose	accidental		S	accidental		S
**Intake**						
Exposure per task / Acute Dose	14,64051724	mg/kg bw	O	30,69216867	mg/kg bw	O
Exposure per day / Chronic Dose	0,120333018	mg/kg bw/day	O	0,2522644	mg/kg bw/day	O

**Disposal**	Task D: excessive ready-for-use paste is disposed to the main drainage, cleaning the bucket and brushes					
	**Primary exposure**	**User**		**Secondary exposure**	**Bystander**	
**Inhalation**						
Concentration of potential exposure	0,128195071	mg/m^3	O	0,128195071	mg/m^3	O
Inhalation	0,001780487	mg/min	O	0,000516341	mg/min	O
Absorption per task	0,02003048	mg/task	O	0,005808839	mg/task	O
Exposure per task / Acute Dose	0,000333841	mg/kg bw	O	0,00069986	mg/kg bw	O
Exposure per day / Chronic Dose	2,7439E-06	mg/kg bw/day	O	5,75228E-06	mg/kg bw/day	O
**Dermal**						
Contact area between ready-for-use paste and skin	0,198	m^2	S			
Volume of contact	19,8	ml	O			
Quantity of contact	12,375	mg	O			
Dermal load (mg/cm^2)	0,00625	mg/cm^2	O			
Absorption (mg)	12,375	mg	O			
Exposure per task / Acute Dose	0,20625	mg/kg bw	O	no		S
Exposure per day / Chronic Dose	0,001695205	mg/kg bw/day	O	no		S
**Oral**						
Exposure / Dose	accidental		S	accidental		S
**Intake**						
Exposure per task / Acute Dose	0,206583841	mg/kg bw	O	0,00069986	mg/kg bw	O
Exposure per day / Chronic Dose	0,001697949	mg/kg bw/day	O	5,75228E-06	mg/kg bw/day	O

Scenario 29: home improvement: (ready-for-use) floor adhesive

Scenario description

				Comments
Name of product	latex adhesive		S	
Physical state product (liquid/solid)	liquid, paste		S	
Density product	1,5	g/cm^3	S	*SDS Ceresit Ceraflux Fliesenkleber*
Concentration of active substance in product	1	%	S	*Frame Formulation*
Concentration of active substance in product (mg/l)	15000	mg/l	O	
Packing unit	1, 3, 7 or 14	kg	S	*Technical Information Ceresit Cereflux*
User	Consumer		S	
Bystander	Children		S	
Temperature	20	°C	D	*room temperature*

Mixing & Loading

Task A: not applicable

Application

Task B: bottom gluing (tiling, laying cork)

Number of tasks per year	3	tasks/yr	S	*three days per year*
Duration of task	480	min/task	S	*8 hours*
Surface (treated per task)	23,04	m^2/task	S	*4,8 x 4.8 m*
Quantity of product used per m^2	2,4	kg/m^2	S	*Technical Information Ceresit Ceraflux 1.5 - 2.4 kg/m²*
Quantity of product used per task	55,296	kg/task	O	*4 buckets = 56 kg*
Quantity of active substance used per task	552960	mg/task	O	
Model inhalation exposure	Exposure to vapour / Instantaneous release (limited to vapour pressure)			
Room volume	58	m^3	S	*Living room*
Model dermal exposure	Direct dermal contact			
Contact area between product and skin	0,2187	m^2	S	*Forearms, Hands, 10% Lower legs*
Contact area between product and skin children	0,0208025	m^2	S	*Hands children*
Model oral exposure	accidental			

Post application phase

Task C: drying time

Number of tasks per year	3	tasks/yr	S	*three days per year*
Duration of task	720	min/task	S	*12 hours*
Quantity of active substance used per task	552960	mg/task	O	
Model inhalation exposure	Exposure to vapour / Instantaneous release (limited to vapour pressure)			
Room volume	58	m^3	S	*Living room*
Model dermal exposure	Direct dermal contact			
Contact area between product and skin	0	m^2	S	*no contact*
Contact area between product and skin children	0	m^2	S	*no contact*
Model oral exposure	accidental			

Disposal

Task D: not applicable

Summary Results Exposure

	User		Consumer	Bystander		Children
Highest potential exposure acute (all amount used is absorbed)		9216	mg/kg bw		66621,68675	mg/kg bw
Highest potential exposure chronic (all amount used is absorbed)		75,74794521	mg/kg bw/day		547,5755075	mg/kg bw/day
Highest potential concentration in air		205,1493218	mg/m^3		205,1493218	mg/m^3
Inhalation acute		42,73944205	mg/kg bw		89,59834839	mg/kg bw
Inhalation chronic		0,351283085	mg/kg bw/day		0,736424781	mg/kg bw/day
Dermal acute		5,4675	mg/kg bw		3,759487952	mg/kg bw
Dermal chronic		0,044938356	mg/kg bw/day		0,030899901	mg/kg bw/day
Oral acute	accidental			accidental		
Oral chronic	accidental			accidental		
Intake acute		48,20694205	mg/kg bw		93,35783634	mg/kg bw
Daily intake chronic		0,396221441	mg/kg bw/day		0,767324682	mg/kg bw/day

U = Undefined, D = Default, S = Set, O = Output, REM = Remark, not used in calculation

Scenario 29: home improvement: (ready-for-use) floor adhesive

Results / Output

**Mixing & Loading**	Task A: not applicable					

**Application**	Task B: bottom gluing (tiling, laying cork)					
	**Primary exposure**	**User**		**Secondary exposure**	**Bystander**	
**Inhalation**						
Concentration of potential exposure	205,1493218	mg/m^3	S	205,1493218	mg/m^3	S
Inhalation	2,849296137	mg/min	O	0,82629588	mg/min	O
Absorption per task	1025,746609	mg/task	O	297,4665167	mg/task	O
Exposure per task / Acute Dose	17,09577682	mg/kg bw	O	35,83933936	mg/kg bw	O
Exposure per day / Chronic Dose	0,140513234	mg/kg bw/day	O	0,294569913	mg/kg bw/day	O
**Dermal**						
Contact area between product and skin	0,2187	m^2	S	0,0208025	m^2	S
Volume of contact	21,87	ml	O	2,08025	ml	O
Quantity of contact	328,05	mg	O	31,20375	mg	O
Dermal load (mg/cm^2)	0,15	mg/cm^2	O	0,15	mg/cm^2	O
Absorption (mg)	328,05	mg	O	31,20375	mg	O
Exposure per task / Acute Dose	5,4675	mg/kg bw	O	3,759487952	mg/kg bw	O
Exposure per day / Chronic Dose	0,044938356	mg/kg bw/day	O	0,030899901	mg/kg bw/day	O
**Oral**						
Exposure / Dose	accidental		S	accidental		S
**Intake**						
Exposure per task / Acute Dose	22,56327682	mg/kg bw	O	39,59882731	mg/kg bw	O
Exposure per day / Chronic Dose	0,18545159	mg/kg bw/day	O	0,325469813	mg/kg bw/day	O

**Post application phase**	Task C: drying time					
	**Primary exposure**	**User**		**Secondary exposure**	**Bystander**	
**Inhalation**						
Concentration of potential exposure	205,1493218	mg/m^3	S	205,1493218	mg/m^3	S
Inhalation	2,849296137	mg/min	O	0,82629588	mg/min	O
Absorption per task	1538,619914	mg/task	O	446,199775	mg/task	O
Exposure per task / Acute Dose	25,64366523	mg/kg bw	O	53,75900903	mg/kg bw	O
Exposure per day / Chronic Dose	0,210769851	mg/kg bw/day	O	0,441854869	mg/kg bw/day	O
**Dermal**						
Exposure per task / Acute Dose	no	mg/kg bw	S	no	mg/kg bw	S
Exposure per day / Chronic Dose	no	mg/kg bw/day	S	no	mg/kg bw/day	S
**Oral**						
Exposure / Dose	accidental		S	accidental		S
**Intake**						
Exposure per task / Acute Dose	25,64366523	mg/kg bw	O	53,75900903	mg/kg bw	O
Exposure per day / Chronic Dose	0,210769851	mg/kg bw/day	O	0,441854869	mg/kg bw/day	O

**Disposal**	Task D: not applicable					

U = Undefined, D = Default, S = Set, O = Output, REM = Remark, not used in calculation

Scenario 30: handicrafts: playing with finger paints

Scenario description

				Comments
Name of product	finger paints		S	
Physical state product (liquid/solid)	liquid		S	
Density product	1,5	g/cm^3	S	*estimated, like latex paints*
Concentration of active substance in product	1	%	S	*Frame Formulation*
Concentration of active substance in product (mg/l)	15000	mg/l	O	
User	Adult		S	
User 2	Children		S	
Temperature	20	°C	D	*room temperature*

Mixing & Loading

Task A: not applicable

Application

Task B: playing with finger paints

Number of tasks per year	52	tasks/yr	S	*once per week*
Duration of task	60	min/task	S	*1 hour playing time*
Surface (treated per task)	1	m^2/task	S	
Quantity of product used per m^2	100	ml/m^2	S	*estimated, like latex paint or varnish*
Quantity of product used per task	100	ml/task	O	
Quantity of active substance used per task	1500	mg/task	O	
Model inhalation exposure	Exposure to vapour / Instantaneous release (limited to vapour pressure)			
Room volume	30	m^3	S	*Children room*
Model dermal exposure	Direct dermal contact			
Contact area between product and skin	0,084	m^2	S	*Hands*
Contact area between product and skin children	0,14601	m^2	S	*Hands, Arms, Head children*
Model oral exposure	accidental			

Post application phase

Task C: drying time

Number of tasks per year	52	tasks/yr	S	*once per week*
Duration of task	120	min/task	S	*2 hour*
Quantity of active substance used per task	1500	mg/task	O	
Model inhalation exposure	Exposure to vapour / Instantaneous release (limited to vapour pressure)			
Room volume	30	m^3	S	*Children room*
Model dermal exposure	Direct dermal contact			
Contact area between product and skin	0	m^2	S	*no direct contact*
Contact area between product and skin children	0	m^2	S	*no direct contact*
Model oral exposure	accidental			

Disposal

Task D: not applicable

Summary Results Exposure

	User	Adult		User 2	Children	
Highest potential exposure acute (all amount used is absorbed)		25	mg/kg bw		180,7228916	mg/kg bw
Highest potential exposure chronic (all amount used is absorbed)		3,561643836	mg/kg bw/day		25,74682291	mg/kg bw/day
Highest potential concentration in air		50	mg/m^3		50	mg/m^3
Inhalation acute		1,5625	mg/kg bw		3,27560241	mg/kg bw
Inhalation chronic		0,22260274	mg/kg bw/day		0,466661165	mg/kg bw/day
Dermal acute		2,1	mg/kg bw		26,3873494	mg/kg bw
Dermal chronic		0,299178082	mg/kg bw/day		3,759293613	mg/kg bw/day
Oral acute	accidental			accidental		
Oral chronic	accidental			accidental		
Intake acute		3,6625	mg/kg bw		29,66295181	mg/kg bw
Daily intake chronic		0,521780822	mg/kg bw/day		4,225954778	mg/kg bw/day

U = Undefined, D = Default, S = Set, O = Output, REM = Remark, not used in calculation

Scenario 30: handicrafts: playing with finger paints

Results / Output

Mixing & Loading	Task A: not applicable					

Application	Task B: playing with finger paints					
	**Primary exposure**	**User**		**Secondary exposure**	**Bystander**	
**Inhalation**						
Concentration of potential exposure	50	mg/m^3	O	50	mg/m^3	O
Inhalation	0,694444444	mg/min	O	0,201388889	mg/min	O
Absorption per task	31,25	mg/task	O	9,0625	mg/task	O
Exposure per task / Acute Dose	0,520833333	mg/kg bw	O	1,09186747	mg/kg bw	O
Exposure per day / Chronic Dose	0,074200913	mg/kg bw/day	O	0,155553722	mg/kg bw/day	O
**Dermal**						
Contact area between product and skin	0,084	m^2	S	0,14601	m^2	S
Volume of contact	8,4	ml	O	14,601	ml	O
Quantity of contact (mg)	126	mg	O	219,015	mg	O
Dermal load (mg/cm^2)	0,15	mg/cm^2	O	0,15	mg/cm^2	O
Absorption (mg)	126	mg	O	219,015	mg	O
Exposure per task / Acute Dose	2,1	mg/kg bw	O	26,3873494	mg/kg bw	O
Exposure per day / Chronic Dose	0,299178082	mg/kg bw/day	O	3,759293613	mg/kg bw/day	O
**Oral**						
Exposure / Dose	accidental		S	accidental		S
**Intake**						
Exposure per task / Acute Dose	2,620833333	mg/kg bw	O	27,47921687	mg/kg bw	O
Exposure per day / Chronic Dose	0,373378995	mg/kg bw/day	O	3,914847335	mg/kg bw/day	O

Post application phase	Task C: drying time					
	**Primary exposure**	**User**		**Secondary exposure**	**Bystander**	
**Inhalation**						
Concentration of potential exposure	50	mg/m^3	O	50	mg/m^3	O
Inhalation	0,694444444	mg/min	O	0,201388889	mg/min	O
Absorption per task	62,5	mg/task	O	18,125	mg/task	O
Exposure per task / Acute Dose	1,041666667	mg/kg bw	O	2,18373494	mg/kg bw	O
Exposure per day / Chronic Dose	0,148401826	mg/kg bw/day	O	0,311107443	mg/kg bw/day	O
**Dermal**						
Exposure per task / Acute Dose	no	mg/kg bw	S	no	mg/kg bw	S
Exposure per day / Chronic Dose	no	mg/kg bw/day	S	no	mg/kg bw/day	S
**Oral**						
Exposure / Dose	accidental		S	accidental		S
**Intake**						
Exposure per task / Acute Dose	1,041666667	mg/kg bw	O	2,18373494	mg/kg bw	O
Exposure per day / Chronic Dose	0,148401826	mg/kg bw/day	O	0,311107443	mg/kg bw/day	O

Disposal	Task D: not applicable					

Scenario 31: repellent: liquid

Scenario description

				Comments
Name of product	milk insect repellent		S	
Physical state product (liquid/solid)	liquid		S	
Density product	1	g/cm^3	D	*ConsExpo 0.9 g/cm^3*
Concentration of active substance in product	1	%	S	*Frame formulation 10 - 20%*
Concentration of active substance in product (mg/l)	10000	mg/l	O	
User	Consumer		S	
User 2	Children		S	
Temperature	20	°C	D	*room temperature*

Mixing & Loading

Task A: not applicable

Application

Task B: rubbing in + leave-on of repellent

Number of tasks per year	84	tasks/yr	S	*three times per day for 4 weeks per year, Consexpo: 54 tasks/yr*
Duration of task	480	min/task	S	*Frame formulation 4-8 hours, ConsExpo 3h*
Quantity of product used per task, adult	4,5	g/task	S	*ConsExpo: 6 g, TGD (2003) for sun cream 8 g*
Quantity of product used per task, child	1,5	g/task	S	*ConsExpo: 1.5 g*
Volume of product used per task, adult	4,5	ml/task	O	*Yap et al. (2000): 0.75 ml per arm and 1.5 ml per leg*
Volume of product used per task, child	1,5	ml/task	O	
Quantity of active substance used per task, adult	45	mg/task	O	
Quantity of active substance used per task, child	15	mg/task	O	
Model inhalation exposure	Exposure to vapour / Instantaneous release (limited to vapour pressure)			
Room volume	1	m^3	S	*cloud around user*
Model dermal exposure	Direct dermal contact			
Contact area between product and skin	1,073	m^2	S	*Upper and lower extremities, head*
Contact area between product and skin children	0,2060625	m^2	S	*Head, arms, legs children*
Contact amount between product and skin, adult	4,5	g/task	S	*Whole amount used*
Contact amount between product and skin, child	1,5	g/task	S	*Whole amount used*
Model oral exposure	accidental			*ConsExpo: hand-mouth contact (adult, 4% in 3h; child 10% in 3h*

Post application phase

Task C: not applicable

Disposal

Task D: not applicable

Summary Results Exposure

	User	Consumer		User 2	Children	
Highest potential exposure acute (all amount used is absorbed)		0,75	mg/kg bw		1,807228916	mg/kg bw
Highest potential exposure chronic (all amount used is absorbed)		0,17260274	mg/kg bw/day		0,415910216	mg/kg bw/day
Highest potential concentration in air		45	mg/m^3		15	mg/m^3
Inhalation acute		0,75	mg/kg bw		1,807228916	mg/kg bw
Inhalation chronic		0,17260274	mg/kg bw/day		0,415910216	mg/kg bw/day
Dermal acute		0,75	mg/kg bw		1,807228916	mg/kg bw
Dermal chronic		0,17260274	mg/kg bw/day		0,415910216	mg/kg bw/day
Oral acute	accidental			accidental		
Oral chronic	accidental			accidental		
Intake acute		0,75	mg/kg bw		1,807228916	mg/kg bw
Daily intake chronic		0,17260274	mg/kg bw/day		0,415910216	mg/kg bw/day

U = Undefined, D = Default, S = Set, O = Output, REM = Remark, not used in calculation

Scenario 31: repellent: liquid

Results / Output

**Mixing & Loading**	Task A: not applicable					

**Application**	Task B: rubbing in + leave-on of repellent					
	**Primary exposure**	**User**		**Primary exposure**	**User 2**	
**Inhalation**						
Concentration of potential exposure	45	mg/m³	O	15	mg/m³	O
Inhalation	0,625	mg/min	O	0,060416667	mg/min	O
Absorption per task	45	mg/task	S	15	mg/task	S
Exposure per task / Acute Dose	0,75	mg/kg bw	O	1,807228916	mg/kg bw	O
Exposure per day / Chronic Dose	0,17260274	mg/kg bw/day	O	0,415910216	mg/kg bw/day	O
**Dermal**						
Contact area between product and skin	1,073	m^2	S	0,2060625	m^2	S
Quantity of contact (mg)	45	mg	S	15	mg	S
Dermal load (mg/cm²)	0,004193849	mg/cm²	O	0,021838035	mg/cm²	O
Absorption (mg)	45	mg	O	15	mg	O
Exposure per task / Acute Dose	0,75	mg/kg bw	O	1,807228916	mg/kg bw	O
Exposure per day / Chronic Dose	0,17260274	mg/kg bw/day	O	0,415910216	mg/kg bw/day	O
**Oral**						
Exposure / Dose	accidental		S	accidental		S
**Intake**						
Exposure per task / Acute Dose	0,75	mg/kg bw	S	1,807228916	mg/kg bw	S
Exposure per day / Chronic Dose	0,17260274	mg/kg bw/day	O	0,415910216	mg/kg bw/day	O

**Post application phase**	Task C: not applicable					

**Disposal**	Task D: not applicable					

U = Undefined, D = Default, S = Set, O = Output, REM = Remark, not used in calculation

Scenario 32: repellent, spray

Scenario description				Comments
Name of product	spray insect repellent		S	
Physical state product (liquid/solid)	liquid		S	
Density product	1	g/cm^3	D	*ConsExpo 0.9 g/cm^3*
Concentration of active substance in product	1	%	S	*Frame formulation 10 - 20%*
Concentration of active substance in product (mg/l)	10000	mg/l	O	
User	Consumer		S	
User 2	Children		S	
Temperature	20	°C	D	*room temperature*
**Mixing & Loading**	Task A: not applicable			
**Application**	Task B: spraying, rubbing in + deposit time of aerosols			
Number of tasks per year	84	tasks/yr	S	*three times per day for 4 weeks per year, Consexpo: 54 tasks/yr*
Duration of task	15	min/task	S	
Quantity of product used per task, adult	4,5	g/task	S	*ConsExpo: 6 g, TGD (2003) for sun cream 8 g*
Quantity of product used per task, child	1,5	g/task	S	*ConsExpo: 1.5 g*
Volume of product used per task, adult	4,5	ml/task	O	*Yap et al. (2000): 0.75 ml per arm and 1.5 ml per leg*
Volume of product used per task, child	1,5	ml/task	O	
Quantity of active substance used per task, adult	45	mg/task	O	
Quantity of active substance used per task, child	15	mg/task	O	
Model inhalation exposure	Exposure to aerosols / Instantaneous release			
Room volume	1	m^3	S	*cloud around user*
Model dermal exposure	Direct dermal contact			
Contact area between product and skin	1,073	m^2	S	*Upper and lower extremities, head*
Contact area between product and skin children	0,2060625	m^2	S	*Head, arms, legs children*
Contact amount between product and skin, adult	4,5	g/task	S	*Whole amount used*
Contact amount between product and skin, child	1,5	g/task	S	*Whole amount used*
Model oral exposure	accidental			
**Post application phase**	Task C: leave-on			
Number of tasks per year	84	tasks/yr	S	*three times per day for 4 weeks per year, Consexpo: 54 tasks/yr*
Duration of task	480	min/task	S	*Frame formulation 4-8 hours, ConsExpo 3h*
Quantity of active substance used per task, adult	45	mg/task	O	
Quantity of active substance used per task, child	15	mg/task	O	
Model inhalation exposure	Exposure to vapour / Instantaneous release (limited to vapour pressure)			
Room volume	1	m^3	S	*cloud around user*
Model dermal exposure	Direct dermal contact			
Contact area between product and skin	1,073	m^2	S	*Upper and lower extremities, head*
Contact area between product and skin children	0,2060625	m^2	S	*Head, arms, legs children*
Contact amount between product and skin	0	g/task	S	*all product is supposed to be absorbed during application*
Model oral exposure	accidental			*ConsExpo: hand-mouth contact (adult, 4% in 3h; child 10% in 3h*
**Disposal**	Task D: not applicable			

Summary Results Exposure

	User	Consumer		User 2	Children	
Highest potential exposure acute (all amount used is absorbed)		0,75	mg/kg bw		1,807228916	mg/kg bw
Highest potential exposure chronic (all amount used is absorbed)		0,17260274	mg/kg bw/day		0,415910216	mg/kg bw/day
Highest potential concentration in air		45	mg/m^3		15	mg/m^3
Inhalation acute		0,75	mg/kg bw		1,807228916	mg/kg bw
Inhalation chronic		0,17260274	mg/kg bw/day		0,415910216	mg/kg bw/day
Dermal acute		0,75	mg/kg bw		1,807228916	mg/kg bw
Dermal chronic		0,17260274	mg/kg bw/day		0,415910216	mg/kg bw/day
Oral acute	accidental			accidental		
Oral chronic	accidental			accidental		
Intake acute		0,75	mg/kg bw		1,807228916	mg/kg bw
Daily intake chronic		0,17260274	mg/kg bw/day		0,415910216	mg/kg bw/day

U = Undefined, D = Default, S = Set, O = Output, REM = Remark, not used in calculation

Scenario 32: repellent, spray

Results / Output

Mixing & Loading	Task A: not applicable					

Application	Task B: spraying, rubbing in + deposit time of aerosols					
	**Primary exposure**	**User**		**Primary exposure**	**User 2**	
**Inhalation**						
Concentration of potential exposure	45	mg/m^3	O	15	mg/m^3	O
Inhalation	0,625	mg/min	O	0,060416667	mg/min	O
Absorption per task	7,03125	mg/task	O	0,6796875	mg/task	O
Exposure per task / Acute Dose	0,1171875	mg/kg bw	O	0,08189006	mg/kg bw	O
Exposure per day / Chronic Dose	0,026969178	mg/kg bw/day	O	0,018845932	mg/kg bw/day	O
**Dermal**						
Contact area between product and skin	1,073	m^2	S	0,2060625	m^2	S
Quantity of contact (mg)	45	mg	S	15	mg	S
Dermal load (mg/cm^2)	0,004193849	mg/cm^2	O	0,007279345	mg/cm^2	O
Absorption (mg)	45	mg	O	15	mg	O
Exposure per task / Acute Dose	0,75	mg/kg bw	O	1,807228916	mg/kg bw	O
Exposure per day / Chronic Dose	0,17260274	mg/kg bw/day	O	0,415910216	mg/kg bw/day	O
**Oral**						
Exposure / Dose	accidental		S	accidental		S
**Intake**						
Exposure per task / Acute Dose	0,75	mg/kg bw	S	1,807228916	mg/kg bw	S
Exposure per day / Chronic Dose	0,17260274	mg/kg bw/day	O	0,415910216	mg/kg bw/day	O

Post application phase	Task C: leave-on					
	**Primary exposure**	**User**		**Secondary exposure**	**Bystander**	
**Inhalation**						
Concentration of potential exposure	45	mg/m^3	O	15	mg/m^3	O
Inhalation	0,625	mg/min	O	0,060416667	mg/min	O
Absorption per task	45	mg/task	S	15	mg/task	S
Exposure per task / Acute Dose	0,75	mg/kg bw	O	1,807228916	mg/kg bw	O
Exposure per day / Chronic Dose	0,17260274	mg/kg bw/day	O	0,415910216	mg/kg bw/day	O
**Dermal**						
Contact area between product and skin	1,073	m^2	S	0,2060625	m^2	S
Quantity of contact (mg)	0	mg	S	0	mg	S
Dermal load (mg/cm^2)	0	mg/cm^2	O	0	mg/cm^2	O
Absorption (mg)	0	mg	O	0	mg	O
Exposure per task / Acute Dose	0	mg/kg bw	O	0	mg/kg bw	O
Exposure per day / Chronic Dose	0	mg/kg bw/day	O	0	mg/kg bw/day	O
**Oral**						
Exposure / Dose	accidental		S	accidental		S
**Intake**						
Exposure per task / Acute Dose	0,75	mg/kg bw	O	1,807228916	mg/kg bw	O
Exposure per day / Chronic Dose	0,17260274	mg/kg bw/day	O	0,415910216	mg/kg bw/day	O

Disposal	Task D: not applicable					

U = Undefined, D = Default, S = Set, O = Output, REM = Remark, not used in calculation

Scenario 33: insecticide: spray, air space

Scenario description

				Comments
Name of product	spray against flying insects		S	
Physical state product (liquid/solid)	liquid		S	
Density product	1	g/cm^3	D	
Concentration of active substance in product	1	%	S	*Frame Formulation*
Concentration of active substance in product (mg/l)	10000	mg/l	O	
User	Consumer		S	
Bystander	Children		S	
Temperature	20	°C	D	*room temperature*

Mixing & Loading

Task A: not applicable

Application

Task B: spraying into the room + deposit time of aerosols

Number of tasks per year	90	tasks/yr	S	*[ConsExpo]*
Duration of task	120	min/task	S	*ConsExpo: exp. duration 240 min, spray duration 20 s*
Quantity of product used per task	7	g/task	S	*ConsExpo: 20 s use duration with 0.35 g/s (10 s spraying time with 0,7 g/s)*
Volume of product used per task	7	ml/task	O	
Quantity of active substance used per task	70	mg/task	O	
Model inhalation exposure	Exposure to aerosols / Instantaneous release			
Room volume	58	m^3	S	*Living room, Consexpo*
Model dermal exposure	Direct dermal contact			
Contact area between product and skin	0,258033333	m^2	S	*Forearms, Hands, 33% Head, 10% Lower legs*
Contact area between product and skin children	0,106472167	m^2	S	*Hands, Arms, 33% Head, 10% Legs children*
Model oral exposure	accidental			

Post application phase

Task C: residence time

Number of tasks per year	90	tasks/yr	S	*[ConsExpo]*
Duration of task	120	min/task	S	*ConsExpo: exp. duration 240 min*
Quantity of active substance used per task	70	mg/task	O	
Model inhalation exposure	Exposure to vapour / Instantaneous release (limited to vapour pressure)			
Room volume	58	m^3	S	*Living room, ConsExpo*
Model dermal exposure	Direct dermal contact			
Contact area between product and skin	0	m^2	S	*no direct contact*
Contact area between product and skin children	0	m^2	S	*no direct contact*
Model oral exposure	accidental			

Disposal

Task D: not applicable

Summary Results Exposure

	User	Consumer	Bystander	Children
Highest potential exposure acute (all amount used is absorbed)	1,166666667	mg/kg bw	8,43373494	mg/kg bw
Highest potential exposure chronic (all amount used is absorbed)	0,287671233	mg/kg bw/day	2,079551081	mg/kg bw/day
Highest potential concentration in air	1,206896552	mg/m^3	1,206896552	mg/m^3
Inhalation acute	0,050287356	mg/kg bw	0,105421687	mg/kg bw
Inhalation chronic	0,012399622	mg/kg bw/day	0,025994389	mg/kg bw/day
Dermal acute	1,166666667	mg/kg bw	8,43373494	mg/kg bw
Dermal chronic	0,287671233	mg/kg bw/day	2,079551081	mg/kg bw/day
Oral acute	accidental		accidental	
Oral chronic	accidental		accidental	
Intake acute	1,166666667	mg/kg bw	8,43373494	mg/kg bw
Daily intake chronic	0,287671233	mg/kg bw/day	2,079551081	mg/kg bw/day

U = Undefined, D = Default, S = Set, O = Output, REM = Remark, not used in calculation

Scenario 33: insecticide: spray, air space

Results / Output

Mixing & Loading	Task A: not applicable					

Application	Task B: spraying into the room + deposit time of aerosols					
	Primary exposure	User		Secondary exposure	Bystander	
**Inhalation**						
Concentration of potential exposure	1,206896552	mg/m^3	O	1,206896552	mg/m^3	O
Inhalation	0,016762452	mg/min	O	0,004861111	mg/min	O
Absorption per task	1,50862069	mg/task	O	0,4375	mg/task	O
Exposure per task / Acute Dose	0,025143678	mg/kg bw	O	0,052710843	mg/kg bw	O
Exposure per day / Chronic Dose	0,006199811	mg/kg bw/day	O	0,012997194	mg/kg bw/day	O
**Dermal**						
Contact area between product and skin	0,258033333	m^2	S	0,106472167	m^2	S
Volume of contact	25,80333333	ml	O	10,64721667	ml	O
Quantity of contact (mg)	70	mg	S	70	mg	S
Dermal load (mg/cm^2)	0,027128278	mg/cm^2	O	0,065744882	mg/cm^2	O
Absorption (mg)	70	mg	O	70	mg	O
Exposure per task / Acute Dose	1,166666667	mg/kg bw	O	8,43373494	mg/kg bw	O
Exposure per day / Chronic Dose	0,287671233	mg/kg bw/day	O	2,079551081	mg/kg bw/day	O
**Oral**						
Exposure / Dose	accidental		S	accidental		S
**Intake**						
Exposure per task / Acute Dose	1,166666667	mg/kg bw	S	8,43373494	mg/kg bw	S
Exposure per day / Chronic Dose	0,287671233	mg/kg bw/day	O	2,079551081	mg/kg bw/day	O

Post application phase	Task C: residence time					
	Primary exposure	User		Secondary exposure	Bystander	
**Inhalation**						
Concentration of potential exposure	1,206896552	mg/m^3	O	1,206896552	mg/m^3	O
Inhalation	0,016762452	mg/min	O	0,004861111	mg/min	O
Absorption per task	1,50862069	mg/task	O	0,4375	mg/task	O
Exposure per task / Acute Dose	0,025143678	mg/kg bw	O	0,052710843	mg/kg bw	O
Exposure per day / Chronic Dose	0,006199811	mg/kg bw/day	O	0,012997194	mg/kg bw/day	O
**Dermal**						
Exposure per task / Acute Dose	no	mg/kg bw	S	no	mg/kg bw	S
Exposure per day / Chronic Dose	no	mg/kg bw/day	S	no	mg/kg bw/day	S
**Oral**						
Exposure / Dose	accidental		S	accidental		S
**Intake**						
Exposure per task / Acute Dose	0,025143678	mg/kg bw	O	0,052710843	mg/kg bw	O
Exposure per day / Chronic Dose	0,006199811	mg/kg bw/day	O	0,012997194	mg/kg bw/day	O

Disposal	Task D: not applicable					

Scenario 33b: insecticide: spray, targetted spot

Scenario description

				Comments
Name of product	spray against wasps or plant lous		S	
Physical state product (liquid/solid)	liquid		S	
Density product	1	g/cm^3	D	
Concentration of active substance in product	1	%	S	*Frame Formulation*
Concentration of active substance in product (mg/l)	10000	mg/l	O	
User	Consumer		S	
Bystander	Children		S	
Temperature	20	°C	D	*room temperature*
**Mixing & Loading**	Task A: not applicable			
**Application**	Task B: spraying (horizontally) onto nest or plant + deposit time of aerosols			
Number of tasks per year	52	tasks/yr	S	*once per week, ConsExpo 9 tasks/yr*
Duration of task	120	min/task	S	*ConsExpo: exp. duration 240 min, spray duration 6 min*
Quantity of product used per task	118,8	g/task	S	*ConsExpo: 6 min, 0.33 g/s*
Volume of product used per task	118,8	ml/task	O	
Quantity of active substance used per task	1188	mg/task	O	
Model inhalation exposure	Exposure to aerosols / Instantaneous release			
Room volume	15	m^3	S	*Kitchen, ConsExpo 20 m^3*
Model dermal exposure	Direct dermal contact			
Contact area between product and skin	0,258033333	m^2	S	*Forearms, Hands, 33% Head, 10% Lower legs*
Contact area between product and skin children	0,106472167	m^2	S	*Hands, Arms, 33% Head, 10% Legs children*
Model oral exposure	accidental			
**Post application phase**	Task C: residence time			
Number of tasks per year	52	tasks/yr	S	*once per week*
Duration of task	120	min/task	S	*ConsExpo: exp. duration 240 min*
Quantity of active substance used per task	1188	mg/task	O	
Model inhalation exposure	Exposure to vapour / Instantaneous release (limited to vapour pressure)			
Room volume	15	m^3	S	*Kitchen, ConsExpo 20 m^3*
Model dermal exposure	Direct dermal contact			
Contact area between product and skin	0	m^2	S	*no direct contact*
Contact area between product and skin children	0	m^2	S	*no direct contact*
Model oral exposure	accidental			
**Disposal**	Task D: not applicable			

Summary Results Exposure

	User	Consumer	Bystander	Children
Highest potential exposure acute (all amount used is absorbed)	19,8	mg/kg bw	143,1325301	mg/kg bw
Highest potential exposure chronic (all amount used is absorbed)	2,820821918	mg/kg bw/day	20,39148374	mg/kg bw/day
Highest potential concentration in air	79,2	mg/m^3	79,2	mg/m^3
Inhalation acute	3,3	mg/kg bw	6,918072289	mg/kg bw
Inhalation chronic	0,470136986	mg/kg bw/day	0,985588381	mg/kg bw/day
Dermal acute	4,300555556	mg/kg bw	12,82797189	mg/kg bw
Dermal chronic	0,612681887	mg/kg bw/day	1,82754668	mg/kg bw/day
Oral acute	accidental		accidental	
Oral chronic	accidental		accidental	
Intake acute	7,600555556	mg/kg bw	19,74604418	mg/kg bw
Daily intake chronic	1,082818874	mg/kg bw/day	2,813135061	mg/kg bw/day

U = Undefined, D = Default, S = Set, O = Output, REM = Remark, not used in calculation

Scenario 33b: insecticide: spray, targetted spot

Results / Output

Mixing & Loading	Task A: not applicable					

Application	Task B: spraying (horizontally) onto nest or plant + deposit time of aerosols					
	**Primary exposure**	**User**		**Secondary exposure**	**Bystander**	
**Inhalation**						
Concentration of potential exposure	79,2	mg/m^3	O	79,2	mg/m^3	O
Inhalation	1,1	mg/min	O	0,319	mg/min	O
Absorption per task	99	mg/task	O	28,71	mg/task	O
Exposure per task / Acute Dose	1,65	mg/kg bw	O	3,459036145	mg/kg bw	O
Exposure per day / Chronic Dose	0,235068493	mg/kg bw/day	O	0,49279419	mg/kg bw/day	O
**Dermal**						
Contact area between product and skin	0,258033333	m^2	S	0,106472167	m^2	S
Volume of contact	25,80333333	ml	O	10,64721667	ml	O
Quantity of contact (mg)	258,0333333	mg	O	106,4721667	mg	O
Dermal load (mg/cm^2)	0,1	mg/cm^2	O	0,1	mg/cm^2	O
Absorption (mg)	258,0333333	mg	O	106,4721667	mg	O
Exposure per task / Acute Dose	4,300555556	mg/kg bw	O	12,82797189	mg/kg bw	O
Exposure per day / Chronic Dose	0,612681887	mg/kg bw/day	O	1,82754668	mg/kg bw/day	O
**Oral**						
Exposure / Dose	accidental		S	accidental		S
**Intake**						
Exposure per task / Acute Dose	5,950555556	mg/kg bw	O	16,28700803	mg/kg bw	O
Exposure per day / Chronic Dose	0,847750381	mg/kg bw/day	O	2,32034087	mg/kg bw/day	O

Post application phase	Task C: residence time					
	**Primary exposure**	**User**		**Secondary exposure**	**Bystander**	
**Inhalation**						
Concentration of potential exposure	79,2	mg/m^3	O	79,2	mg/m^3	O
Inhalation	1,1	mg/min	O	0,319	mg/min	O
Absorption per task	99	mg/task	O	28,71	mg/task	O
Exposure per task / Acute Dose	1,65	mg/kg bw	O	3,459036145	mg/kg bw	O
Exposure per day / Chronic Dose	0,235068493	mg/kg bw/day	O	0,49279419	mg/kg bw/day	O
**Dermal**						
Exposure per task / Acute Dose	no	mg/kg bw	S	no	mg/kg bw	S
Exposure per day / Chronic Dose	no	mg/kg bw/day	S	no	mg/kg bw/day	S
**Oral**						
Exposure / Dose	accidental		S	accidental		S
**Intake**						
Exposure per task / Acute Dose	1,65	mg/kg bw	O	3,459036145	mg/kg bw	O
Exposure per day / Chronic Dose	0,235068493	mg/kg bw/day	O	0,49279419	mg/kg bw/day	O

Disposal	Task D: not applicable					

U = Undefined, D = Default, S = Set, O = Output, REM = Remark, not used in calculation

Scenario 33c: insecticide: spray, crack and crevice

Scenario description

				Comments
Name of product	spray against ants or silverfish		S	
Physical state product (liquid/solid)	liquid		S	
Density product	1	g/cm³	D	
Concentration of active substance in product	1	%	S	*Frame Formulation*
Concentration of active substance in product (mg/l)	10000	mg/l	O	
User	Consumer		S	
Bystander	Children		S	
Temperature	20	°C	D	*room temperature*

Mixing & Loading

Task A: not applicable

Application

Task B: spraying down in crack and crevice onto bottom + deposit time of aerosols

Number of tasks per year	52	tasks/yr	S	*once per week, ConsExpo 9 tasks/yr*
Duration of task	120	min/task	S	*ConsExpo: exp. duration 240 min, spray duration 4 min*
Quantity of product used per task	79,2	g/task	S	*ConsExpo: 4 min, 0.33 g/s*
Volume of product used per task	79,2	ml/task	O	
Quantity of active substance used per task	792	mg/task	O	
Model inhalation exposure	Exposure to aerosols / Instantaneous release			
Room volume	15	m³	S	*Kitchen, ConsExpo 20 m³*
Model dermal exposure	Direct dermal contact			
Contact area between product and skin	0,2187	m²	S	*Forearms, Hands, 10% Lower legs*
Contact area between product and skin children	0,0826605	m²	S	*Hands, Arms, 10% Legs children*
Model oral exposure	accidental			

Post application phase

Task C: residence time

Number of tasks per year	52	tasks/yr	S	*once per week*
Duration of task	120	min/task	S	*ConsExpo: exp. duration 240 min*
Quantity of active substance used per task	792	mg/task	O	
Model inhalation exposure	Exposure to vapour / Instantaneous release (limited to vapour pressure)			
Room volume	15	m³	S	*Kitchen, ConsExpo 20 m³*
Model dermal exposure	Direct dermal contact			
Contact area between product and skin	0	m²	S	*no direct contact*
Contact area between product and skin children	0	m²	S	*no direct contact*
Model oral exposure	accidental			

Disposal

Task D: not applicable

Summary Results Exposure

	User	Consumer		Bystander	Children
Highest potential exposure acute (all amount used is absorbed)		13,2	mg/kg bw	95,42168675	mg/kg bw
Highest potential exposure chronic (all amount used is absorbed)		1,880547945	mg/kg bw/day	13,5943225	mg/kg bw/day
Highest potential concentration in air		52,8	mg/m³	52,8	mg/m³
Inhalation acute		2,2	mg/kg bw	4,612048193	mg/kg bw
Inhalation chronic		0,313424658	mg/kg bw/day	0,657058921	mg/kg bw/day
Dermal acute		3,645	mg/kg bw	9,959096386	mg/kg bw
Dermal chronic		0,519287671	mg/kg bw/day	1,41883017	mg/kg bw/day
Oral acute	accidental			accidental	
Oral chronic	accidental			accidental	
Intake acute		5,845	mg/kg bw	14,57114458	mg/kg bw
Daily intake chronic		0,832712329	mg/kg bw/day	2,075889091	mg/kg bw/day

U = Undefined, D = Default, S = Set, O = Output, REM = Remark, not used in calculation

Scenario 33c: insecticide: spray, crack and crevice

Results / Output

Mixing & Loading	Task A: not applicable					

Application	Task B: spraying down in crack and crevice onto bottom + deposit time of aerosols					
	**Primary exposure**	**User**		**Secondary exposure**	**Bystander**	
**Inhalation**						
Concentration of potential exposure	52,8	mg/m^3	O	52,8	mg/m^3	O
Inhalation	0,733333333	mg/min	O	0,212666667	mg/min	O
Absorption per task	66	mg/task	O	19,14	mg/task	O
Exposure per task / Acute Dose	1,1	mg/kg bw	O	2,306024096	mg/kg bw	O
Exposure per day / Chronic Dose	0,156712329	mg/kg bw/day	O	0,32852946	mg/kg bw/day	O
**Dermal**						
Contact area between product and skin	0,2187	m^2	S	0,0826605	m^2	S
Volume of contact	21,87	ml	O	8,26605	ml	O
Quantity of contact (mg)	218,7	mg	O	82,6605	mg	O
Dermal load (mg/cm^2)	0,1	mg/cm^2	O	0,1	mg/cm^2	O
Absorption (mg)	218,7	mg	O	82,6605	mg	O
Exposure per task / Acute Dose	3,645	mg/kg bw	O	9,959096386	mg/kg bw	O
Exposure per day / Chronic Dose	0,519287671	mg/kg bw/day	O	1,41883017	mg/kg bw/day	O
**Oral**						
Exposure / Dose	accidental		S	accidental		S
**Intake**						
Exposure per task / Acute Dose	4,745	mg/kg bw	O	12,26512048	mg/kg bw	O
Exposure per day / Chronic Dose	0,676	mg/kg bw/day	O	1,74735963	mg/kg bw/day	O

Post application phase	Task C: residence time					
	**Primary exposure**	**User**		**Secondary exposure**	**Bystander**	
**Inhalation**						
Concentration of potential exposure	52,8	mg/m^3	O	52,8	mg/m^3	O
Inhalation	0,733333333	mg/min	O	0,212666667	mg/min	O
Absorption per task	66	mg/task	O	19,14	mg/task	O
Exposure per task / Acute Dose	1,1	mg/kg bw	O	2,306024096	mg/kg bw	O
Exposure per day / Chronic Dose	0,156712329	mg/kg bw/day	O	0,32852946	mg/kg bw/day	O
**Dermal**						
Exposure per task / Acute Dose	no	mg/kg bw	S	no	mg/kg bw	S
Exposure per day / Chronic Dose	no	mg/kg bw/day	S	no	mg/kg bw/day	S
**Oral**						
Exposure / Dose	accidental		S	accidental		S
**Intake**						
Exposure per task / Acute Dose	1,1	mg/kg bw	O	2,306024096	mg/kg bw	O
Exposure per day / Chronic Dose	0,156712329	mg/kg bw/day	O	0,32852946	mg/kg bw/day	O

Disposal	Task D: not applicable					

Scenario 34: insecticide: liquid, crack and crevice

Scenario description

				Comments
Name of product	ant killer		S	
Physical state product (liquid/solid)	liquid		S	
Density product	1	g/cm^3	D	
Concentration of active substance in product (concentrate)	1	%	S	*Frame Formulation*
Concentration of active substance in product (concentrate) (mg/l)	10000	mg/l	O	
User	Consumer		S	
Bystander	Children		S	
Temperature	20	°C	D	*room temperature*
**Mixing & Loading**	Task A: simple dilution with water in bucket			
Number of tasks per year	52	tasks/yr	S	*once per week*
Duration of task	1,333333333	min/task	S	*80 sec. [mixing spray, TNsG 2002 p. 252]*
Volume of product used per task	1,4	ml/task	S	*Info Online Apotheke*
Quantity of water used per task	5	L/task	S	*Frame Formulation*
Quantity of product used per task	1,4	g/task	O	
Quantity of active substance used per task	14	mg/task	S	
Concentration of active substance in ready-for-use solution	2,8	mg/l	O	
Model inhalation exposure	Exposure to vapour / Fugacity concept (equilibrium between ready-for-use solution and air, limited to vapour pressure)			
Room volume	1	m^3	S	*cloud around user*
REM Evaporation from mixture, release area concentrate	*0,002*	*m^2*	*S*	*bottle diameter 5 cm [TNsG 2002 p. 252]*
REM Evaporation from mixture, release area ready-for-use solution	*0,070685835*	*m^2*	*S*	*bucket diameter 30 cm*
Model dermal exposure	Direct dermal contact			
Contact area between product (concentrate) and skin	0,0168	m^2	S	*Fingertips*
Model oral exposure	accidental			
**Application**	Task B: pouring the ready-for-use solution			
Number of tasks per year	52	tasks/yr	S	*once per week*
Duration of task	10	min/task	S	
Surface (treated per task)	5	m^2/task	S	*Frame Formulation*
Quantity of ready-for-use solution used per m^2	1000	ml/m^2	S	
Quantity of ready-for-use solution used per task	5000	ml/task	O	
Concentration of active substance in ready-for-use solution	2,8	mg/l	O	
Quantity of active substance used per task	14	mg/task	O	
Model inhalation exposure	Exposure to vapour / Instantaneous release (limited to vapour pressure)			
Room volume	1	m^3	S	*cloud around user*
Model dermal exposure	Direct dermal contact			
Contact area between ready-for-use solution and skin	0,2187	m^2	S	*Forearms, Hands, 10% Lower legs*
Model oral exposure	accidental			
**Post application phase**	Task C: residence time			
Number of tasks per year	52	tasks/yr	S	*once per week*
Duration of task	480	min/task	S	*8 hours*
Concentration of active substance in ready-for-use solution	2,8	mg/l	O	
Quantity of active substance used per task	14	mg/task	O	
Model inhalation exposure	Exposure to vapour / Instantaneous release (limited to vapour pressure)			
Room volume	15	m^3	S	*Terrace (Kitchen)*
Model dermal exposure	Direct dermal contact			
Contact area between ready-for-use solution and skin	0,042	m^2	S	*50% Hands*
Contact area between ready-for-use solution and skin children	0,01040125	m^2	S	*50% Hands Children*
Model oral exposure	accidental			
**Disposal**	Task D: excessive ready-for-use solution is disposed to the main drainage			
Number of tasks per year	52	tasks/yr	S	*once per week*
Duration of task	1,333333333	min/task	S	*80 sec. [see mixing & loading]*
Volume of ready-for-use solution disposed per task	0	L/task	S	
Concentration of active substance in ready-for-use solution	2,8	mg/l	O	
Quantity of active substance disposed per task	0	mg/task	O	
Model inhalation exposure	Exposure to vapour / Fugacity concept (equilibrium between ready-for-use solution and air, limited to vapour pressure)			
Room volume	1	m^3	S	
REM Evaporation from mixture, release area ready-for-use solution	*0,070685835*	*m^2*	*S*	*bucket diameter 30 cm*
Model dermal exposure	Direct dermal contact			
Contact area between ready-for-use solution and skin	0,198	m^2	S	*Hands, Forearms*
Model oral exposure	accidental			

Summary Results Exposure

	User	Consumer		Bystander	Children	
Highest potential exposure acute (all amount used is absorbed)		0,233333333	mg/kg bw		1,686746988	mg/kg bw
Highest potential exposure chronic (all amount used is absorbed)		0,033242009	mg/kg bw/day		0,24030368	mg/kg bw/day
Highest potential concentration in air		14	mg/m^3		14	mg/m^3
Inhalation acute		0,102083466	mg/kg bw		0,214006303	mg/kg bw
Inhalation chronic		0,014543398	mg/kg bw/day		0,030488569	mg/kg bw/day
Dermal acute		0,233333333	mg/kg bw		0,000350886	mg/kg bw
Dermal chronic		0,033242009	mg/kg bw/day		4,99892E-05	mg/kg bw/day
Oral acute	accidental			accidental		
Oral chronic	accidental			accidental		
Intake acute		0,233333333	mg/kg bw		0,214357188	mg/kg bw
Daily intake chronic		0,033242009	mg/kg bw/day		0,030538558	mg/kg bw/day

U = Undefined, D = Default, S = Set, O = Output, REM = Remark, not used in calculation

Scenario 34: insecticide: liquid, crack and crevice

Results / Output

Mixing & Loading	Task A: simple dilution with water in bucket					
	Primary exposure	User		Secondary exposure	Bystander	
**Inhalation**						
Concentration of potential exposure	0,000574395	mg/m^3	O	0,000574395	mg/m^3	O
Inhalation	7,9777E-06	mg/min	O	2,31353E-06	mg/min	O
Absorption per task	7,9777E-06	mg/task	O	2,31353E-06	mg/task	O
Exposure per task / Acute Dose	1,32962E-07	mg/kg bw	O	2,78739E-07	mg/kg bw	O
Exposure per day / Chronic Dose	1,89425E-08	mg/kg bw/day	O	3,97108E-08	mg/kg bw/day	O
**Dermal**						
Contact area between product (concentrate) and skin	0,0168	m^2	S			
Volume of contact	1,68	ml	O			
Quantity of contact (mg)	14	mg	S			
Dermal load (mg/cm^2)	0,083333333	mg/cm^2	O			
Absorption (mg)	14	mg	O			
Exposure per task / Acute Dose	0,233333333	mg/kg bw	O	no		S
Exposure per day / Chronic Dose	0,033242009	mg/kg bw/day	O	no		S
**Oral**						
Exposure / Dose	accidental		S	accidental		S
**Intake**						
Exposure per task / Acute Dose	0,233333333	mg/kg bw	S	2,78739E-07	mg/kg bw	O
Exposure per day / Chronic Dose	0,033242009	mg/kg bw/day	O	3,97108E-08	mg/kg bw/day	O

Application	Task B: pouring the ready-for-use solution					
	Primary exposure	User		Secondary exposure	Bystander	
**Inhalation**						
Concentration of potential exposure	14	mg/m^3	O	14	mg/m^3	O
Inhalation	0,194444444	mg/min	O	0,056388889	mg/min	O
Absorption per task	1,458333333	mg/task	O	0,422916667	mg/task	O
Exposure per task / Acute Dose	0,024305556	mg/kg bw	O	0,050953815	mg/kg bw	O
Exposure per day / Chronic Dose	0,003462709	mg/kg bw/day	O	0,007259174	mg/kg bw/day	O
**Dermal**						
Contact area between ready-for-use solution and skin	0,2187	m^2	S			
Volume of contact	21,87	ml	O			
Quantity of contact (mg)	0,061236	mg	O			
Dermal load (mg/cm^2)	0,000028	mg/cm^2	O			
Absorption (mg)	0,061236	mg	O			
Exposure per task / Acute Dose	0,0010206	mg/kg bw	O	no		S
Exposure per day / Chronic Dose	0,000145401	mg/kg bw/day	O	no		S
**Oral**						
Exposure / Dose	accidental		S	accidental		S
**Intake**						
Exposure per task / Acute Dose	0,025326156	mg/kg bw	O	0,050953815	mg/kg bw	O
Exposure per day / Chronic Dose	0,00360811	mg/kg bw/day	O	0,007259174	mg/kg bw/day	O

Post application phase	Task C: residence time					
	Primary exposure	User		Secondary exposure	Bystander	
**Inhalation**						
Concentration of potential exposure	0,933333333	mg/m^3	O	0,933333333	mg/m^3	O
Inhalation	0,012962963	mg/min	O	0,003759259	mg/min	O
Absorption per task	4,666666667	mg/task	O	1,353333333	mg/task	O
Exposure per task / Acute Dose	0,077777778	mg/kg bw	O	0,163052209	mg/kg bw	O
Exposure per day / Chronic Dose	0,01108067	mg/kg bw/day	O	0,023229356	mg/kg bw/day	O
**Dermal**						
Contact area between ready-for-use solution and skin	0,042	m^2	S	0,01040125	m^2	S
Volume of contact	4,2	ml	O	1,040125	ml	O
Quantity of contact (mg)	0,01176	mg	O	0,00291235	mg	O
Dermal load (mg/cm^2)	0,000028	mg/cm^2	O	0,000028	mg/cm^2	O
Absorption (mg)	0,01176	mg	O	0,00291235	mg	O
Exposure per task / Acute Dose	0,000196	mg/kg bw	O	0,000350886	mg/kg bw	O
Exposure per day / Chronic Dose	2,79233E-05	mg/kg bw/day	O	4,99892E-05	mg/kg bw/day	O
**Oral**						
Exposure / Dose	accidental		S	accidental		S
**Intake**						
Exposure per task / Acute Dose	0,077973778	mg/kg bw	O	0,163403094	mg/kg bw	O
Exposure per day / Chronic Dose	0,011108593	mg/kg bw/day	O	0,023279345	mg/kg bw/day	O

Disposal	Task D: excessive ready-for-use solution is disposed to the main drainage					
	Primary exposure	User		Secondary exposure	Bystander	
**Inhalation**						
Concentration of potential exposure	0	mg/m^3	O	0	mg/m^3	O
Inhalation	0	mg/min	O	0	mg/min	O
Absorption per task	0	mg/task	O	0	mg/task	O
Exposure per task / Acute Dose	0	mg/kg bw	O	0	mg/kg bw	O
Exposure per day / Chronic Dose	0	mg/kg bw/day	O	0	mg/kg bw/day	O
**Dermal**						
Contact area between ready-for-use solution and skin	0,198	m^2	S			
Volume of contact	19,8	ml	O			
Quantity of contact (mg)	0	mg	S			
Dermal load (mg/cm^2)	0	mg/cm^2	O			
Absorption (mg)	0	mg	O			
Exposure per task / Acute Dose	0	mg/kg bw	O	no		S
Exposure per day / Chronic Dose	0	mg/kg bw/day	O	no		S
**Oral**						
Exposure / Dose	accidental		S	accidental		S
**Intake**						
Exposure per task / Acute Dose	0	mg/kg bw	O	0	mg/kg bw	O
Exposure per day / Chronic Dose	0	mg/kg bw/day	O	0	mg/kg bw/day	O

U = Undefined, D = Default, S = Set, O = Output, REM = Remark, not used in calculation

Scenario 35: insecticide: dilution of powder, crack and crevice

Scenario description

			Comments
Name of product	ant killer	S	
Physical state product (liquid/solid)	solid	S	
Density product	1 g/cm^3	D	
Concentration of active substance in product (concentrate)	1 %	S	*Frame Formulation*
Concentration of active substance in product (concentrate) (mg/l)	10000 mg/l	O	
User	Consumer	S	
Bystander	Children	S	
Temperature	20 °C	D	*room temperature*

Mixing & Loading

	Task A: simple dilution with water in bucket		
Number of tasks per year	52 tasks/yr	S	*once per week*
Duration of task	1,333333333 min/task	S	*80 sec. [mixing spray TNsG 2002 p. 252]*
Quantity of product used per task	50 g/task	S	*Frame formulation 10 g/L*
Quantity of water used per task	5 L/task	S	
Volume of product used per task	50 ml/task	S	
Quantity of active substance used per task	500 mg/task	S	
Concentration of active substance in ready-for-use solution	100 mg/l	O	
Model inhalation exposure	Exposure to vapour / Fugacity concept (equilibrium between ready-for-use solution and air, limited to vapour pressure)		
Room volume	1 m^3	S	*cloud around user*
REM Evaporation from mixture, release area concentrate	*0,002 m^2*	*S*	*bottle diameter 5 cm [TNsG 2002 p. 252]*
REM Evaporation from mixture, release area ready-for-use solution	*0,070685835 m^2*	*S*	*bucket diameter 30 cm*
Model dermal exposure	Direct dermal contact		
Contact area between product and skin	0,042 m^2	S	*50% Hands*
Contact amount between active substance and skin	0,5 mg	S	*0.1% of whole amount used [see HERA Perborate]*
Model oral exposure	accidental		

Application

	Task B: pouring the ready-for-use solution		
Number of tasks per year	52 tasks/yr	S	*once per week*
Duration of task	15 min/task	S	
Surface (treated per task)	4 m^2/task	S	
Quantity of ready-for-use solution used per m^2	1000 ml/m^2	S	*Frame formulation 1L per m^2*
Quantity of ready-for-use solution used per task	4000 ml/task	O	
Concentration of active substance in ready-for-use solution	100 mg/l	O	
Quantity of active substance used per task	400 mg/task	O	
Model inhalation exposure	Exposure to vapour / Instantaneous release (limited to vapour pressure)		
Room volume	1 m^3	S	*cloud around user*
Model dermal exposure	Direct dermal contact		
Contact area between ready-for-use solution and skin	0,2187 m^2	S	*Forearms, Hands, 10% Lower legs*
Model oral exposure	accidental		

Post application phase

	Task C: residence time		
Number of tasks per year	52 tasks/yr	S	*once per week*
Duration of task	480 min/task	S	*8 hours*
Concentration of active substance in ready-for-use solution	100 mg/l	O	
Quantity of active substance used per task	400 mg/task	O	
Model inhalation exposure	Exposure to vapour / Instantaneous release (limited to vapour pressure)		
Room volume	15 m^3	S	*Terrace (Kitchen)*
Model dermal exposure	Direct dermal contact		
Contact area between ready-for-use solution and skin	0,042 m^2	S	*50% Hands*
Contact area between ready-for-use solution and skin children	0,01040125 m^2	S	*50% Hands Children*
Model oral exposure	accidental		

Disposal

	Task D: excessive ready-for-use solution is disposed to the main drainage		
Number of tasks per year	52 tasks/yr	S	*once per week*
Duration of task	1,333333333 min/task	S	*80 sec. [see mixing & loading]*
Volume of ready-for-use solution disposed per task	1 L/task	S	
Concentration of active substance in ready-for-use solution	100 mg/l	O	
Quantity of active substance disposed per task	100 mg/task	O	
Model inhalation exposure	Exposure to vapour / Fugacity concept (equilibrium between ready-for-use solution and air, limited to vapour pressure)		
Room volume	1 m^3	S	*cloud around user*
REM Evaporation from mixture, release area ready-for-use solution	*0,070685835 m^2*	*S*	*bucket diameter 30 cm*
Model dermal exposure	Direct dermal contact		
Contact area between ready-for-use solution and skin	0,198 m^2	S	*Hands, Forearms*
Model oral exposure	accidental		

Summary Results Exposure

	User	Consumer	Bystander	Children
Highest potential exposure acute (all amount used is absorbed)		8,333333333 mg/kg bw		60,24096386 mg/kg bw
Highest potential exposure chronic (all amount used is absorbed)		1,187214612 mg/kg bw/day		8,582274303 mg/kg bw/day
Highest potential concentration in air		205,1493218 mg/m^3		205,1493218 mg/m^3
Inhalation acute		2,756474744 mg/kg bw		5,778633801 mg/kg bw
Inhalation chronic		0,392703251 mg/kg bw/day		0,823257418 mg/kg bw/day
Dermal acute		0,084783333 mg/kg bw		0,012531627 mg/kg bw
Dermal chronic		0,012078721 mg/kg bw/day		0,001785328 mg/kg bw/day
Oral acute	accidental		accidental	
Oral chronic	accidental		accidental	
Intake acute		2,841258078 mg/kg bw		5,791165428 mg/kg bw
Daily intake chronic		0,404781973 mg/kg bw/day		0,825042746 mg/kg bw/day

Scenario 35: insecticide: dilution of powder, crack and crevice

Results / Output

**Mixing & Loading**	Task A: simple dilution with water in bucket					
	**Primary exposure**	**User**		**Secondary exposure**	**Bystander**	
**Inhalation**						
Concentration of potential exposure	0,02051409	mg/m^3	O	0,02051409	mg/m^3	O
Inhalation	0,000284918	mg/min	O	8,26262E-05	mg/min	O
Absorption per task	0,000284918	mg/task	O	8,26262E-05	mg/task	O
Exposure per task / Acute Dose	4,74863E-06	mg/kg bw	O	9,95496E-06	mg/kg bw	O
Exposure per day / Chronic Dose	6,76517E-07	mg/kg bw/day	O	1,41824E-06	mg/kg bw/day	O
**Dermal**						
Contact area between product (powder) and skin	0,042	m^2	S			
Quantity of contact (mg)	0,5	mg	S			
Dermal load (mg/cm^2)	0,001190476	mg/cm^2	O			
Absorption (mg)	0,5	mg	O			
Exposure per task / Acute Dose	0,008333333	mg/kg bw	O	no		S
Exposure per day / Chronic Dose	0,001187215	mg/kg bw/day	O	no		S
**Oral**						
Exposure / Dose	accidental		S	accidental		S
**Intake**						
Exposure per task / Acute Dose	0,008338082	mg/kg bw	O	9,95496E-06	mg/kg bw	O
Exposure per day / Chronic Dose	0,001187891	mg/kg bw/day	O	1,41824E-06	mg/kg bw/day	O

**Application**	Task B: pouring the ready-for-use solution					
	**Primary exposure**	**User**		**Secondary exposure**	**Bystander**	
**Inhalation**						
Concentration of potential exposure	205,1493218	mg/m^3	S	205,1493218	mg/m^3	S
Inhalation	2,849296137	mg/min	O	0,82629588	mg/min	O
Absorption per task	32,05458154	mg/task	O	9,295828646	mg/task	O
Exposure per task / Acute Dose	0,534243026	mg/kg bw	O	1,119979355	mg/kg bw	O
Exposure per day / Chronic Dose	0,076111335	mg/kg bw/day	O	0,159558703	mg/kg bw/day	O
**Dermal**						
Contact area between ready-for-use solution and skin	0,2187	m^2	S			
Volume of contact	21,87	ml	O			
Quantity of contact (mg)	2,187	mg	O			
Dermal load (mg/cm^2)	0,001	mg/cm^2	O			
Absorption (mg)	2,187	mg	O			
Exposure per task / Acute Dose	0,03645	mg/kg bw	O	no		S
Exposure per day / Chronic Dose	0,005192877	mg/kg bw/day	O	no		S
**Oral**						
Exposure / Dose	accidental		S	accidental		S
**Intake**						
Exposure per task / Acute Dose	0,570693026	mg/kg bw	O	1,119979355	mg/kg bw	O
Exposure per day / Chronic Dose	0,081304212	mg/kg bw/day	O	0,159558703	mg/kg bw/day	O

**Post application phase**	Task C: residence time					
	**Primary exposure**	**User**		**Secondary exposure**	**Bystander**	
**Inhalation**						
Concentration of potential exposure	26,66666667	mg/m^3	O	26,66666667	mg/m^3	O
Inhalation	0,37037037	mg/min	O	0,107407407	mg/min	O
Absorption per task	133,3333333	mg/task	O	38,66666667	mg/task	O
Exposure per task / Acute Dose	2,222222222	mg/kg bw	O	4,658634538	mg/kg bw	O
Exposure per day / Chronic Dose	0,316590563	mg/kg bw/day	O	0,663695879	mg/kg bw/day	O
**Dermal**						
Contact area between ready-for-use solution and skin	0,042	m^2	S	0,01040125	m^2	S
Volume of contact	4,2	ml	O	1,040125	ml	O
Quantity of contact (mg)	0,42	mg	O	0,1040125	mg	O
Dermal load (mg/cm^2)	0,001	mg/cm^2	O	0,001	mg/cm^2	O
Absorption (mg)	0,42	mg	O	0,1040125	mg	O
Exposure per task / Acute Dose	0,007	mg/kg bw	O	0,012531627	mg/kg bw	O
Exposure per day / Chronic Dose	0,00099726	mg/kg bw/day	O	0,001785328	mg/kg bw/day	O
**Oral**						
Exposure / Dose	accidental		S	accidental		S
**Intake**						
Exposure per task / Acute Dose	2,229222222	mg/kg bw	O	4,671166165	mg/kg bw	O
Exposure per day / Chronic Dose	0,317587823	mg/kg bw/day	O	0,665481207	mg/kg bw/day	O

**Disposal**	Task D: excessive ready-for-use solution is disposed to the main drainage					
	**Primary exposure**	**User**		**Secondary exposure**	**Bystander**	
**Inhalation**						
Concentration of potential exposure	0,020510724	mg/m^3	O	0,020510724	mg/m^3	O
Inhalation	0,000284871	mg/min	O	8,26126E-05	mg/min	O
Absorption per task	0,000284871	mg/task	O	8,26126E-05	mg/task	O
Exposure per task / Acute Dose	4,74785E-06	mg/kg bw	O	9,95333E-06	mg/kg bw	O
Exposure per day / Chronic Dose	6,76406E-07	mg/kg bw/day	O	1,41801E-06	mg/kg bw/day	O
**Dermal**						
Contact area between ready-for-use solution and skin	0,198	m^2	S			
Volume of contact	19,8	ml	O			
Quantity of contact (mg)	1,98	mg	O			
Dermal load (mg/cm^2)	0,001	mg/cm^2	O			
Absorption (mg)	1,98	mg	O			
Exposure per task / Acute Dose	0,033	mg/kg bw	O	no		S
Exposure per day / Chronic Dose	0,00470137	mg/kg bw/day	O	no		S
**Oral**						
Exposure / Dose	accidental		S	accidental		S
**Intake**						
Exposure per task / Acute Dose	0,033004748	mg/kg bw	O	9,95333E-06	mg/kg bw	O
Exposure per day / Chronic Dose	0,004702046	mg/kg bw/day	O	1,41801E-06	mg/kg bw/day	O

Scenario 35b: insecticide: powder, crack and crevice

Scenario description

				Comments
Name of product	ant killer		S	
Physical state product (liquid/solid)	solid		S	
Density product	1	g/cm^3	D	
Concentration of active substance in product (concentrate)	1	%	S	*Frame Formulation*
Concentration of active substance in product (concentrate) (mg/l)	10000	mg/l	O	
User	Consumer		S	
Bystander	Children		S	
Temperature	20	°C	D	*room temperature*

Mixing & Loading

Task A: not applicable

Application

Task B: throwing the powder

Number of tasks per year	52	tasks/yr	S	*once per week*
Duration of task	15	min/task	S	
Surface (treated per task)	1	m^2/task	S	
Quantity of product used per m^2	8	g/m^2	S	*Frame formulation*
Quantity of product used per task	8	g/task	O	
Volume of product used per task	8	ml/task	O	
Quantity of active substance used per task	80	mg/task	O	
Model inhalation exposure	Exposure to vapour / Instantaneous release (limited to vapour pressure)			
Room volume	1	m^3	S	*cloud around user*
Model dermal exposure	Direct dermal contact			
Contact area between product and skin	0,042	m^2	S	*50% Hands*
Contact amount between active substance and skin	0,08	mg	S	*0.1% of whole amount used [see HERA Perborate]*
Model oral exposure	accidental			

Post application phase

Task C: residence time

Number of tasks per year	52	tasks/yr	S	*once per week*
Duration of task	480	min/task	S	*8 hours*
Quantity of active substance used per task	80	mg/task	O	
Model inhalation exposure	Exposure to vapour / Instantaneous release (limited to vapour pressure)			
Room volume	15	m^3	S	*Terrace (Kitchen)*
Model dermal exposure	Direct dermal contact			
Contact area between product and skin	0,042	m^2	S	*50% Hands*
Contact area between product and skin children	0,01040125	m^2	S	*50% Hands Children*
Contact amount between active substance and skin	0,08	mg	S	*0.1% of whole amount used*
Model oral exposure	accidental			

Disposal

Task D: not applicable

Summary Results Exposure

	User	Consumer	Bystander	Children
Highest potential exposure acute (all amount used is absorbed)	1,333333333	mg/kg bw	9,638554217	mg/kg bw
Highest potential exposure chronic (all amount used is absorbed)	0,189954338	mg/kg bw/day	1,373163888	mg/kg bw/day
Highest potential concentration in air	80	mg/m^3	80	mg/m^3
Inhalation acute	0,652777778	mg/kg bw	1,368473896	mg/kg bw
Inhalation chronic	0,092998478	mg/kg bw/day	0,194960665	mg/kg bw/day
Dermal acute	0,002666667	mg/kg bw	0,009638554	mg/kg bw
Dermal chronic	0,000379909	mg/kg bw/day	0,001373164	mg/kg bw/day
Oral acute	accidental		accidental	
Oral chronic	accidental		accidental	
Intake acute	0,655444444	mg/kg bw	1,37811245	mg/kg bw
Daily intake chronic	0,093378387	mg/kg bw/day	0,196333828	mg/kg bw/day

U = Undefined, D = Default, S = Set, O = Output, REM = Remark, not used in calculation

Scenario 35b: insecticide: powder, crack and crevice

Results / Output

Mixing & Loading	Task A: not applicable					

Application	Task B: throwing the powder					
	Primary exposure	User		Secondary exposure	Bystander	
**Inhalation**						
Concentration of potential exposure	80	mg/m^3	O	80	mg/m^3	O
Inhalation	1,111111111	mg/min	O	0,322222222	mg/min	O
Absorption per task	12,5	mg/task	O	3,625	mg/task	O
Exposure per task / Acute Dose	0,208333333	mg/kg bw	O	0,436746988	mg/kg bw	O
Exposure per day / Chronic Dose	0,029680365	mg/kg bw/day	O	0,062221489	mg/kg bw/day	O
**Dermal**						
Contact area between product and skin	0,042	m^2	S			
Quantity of contact (mg)	0,08	mg	S			
Dermal load (mg/cm^2)	0,000190476	mg/cm^2	O			
Absorption (mg)	0,08	mg	O			
Exposure per task / Acute Dose	0,001333333	mg/kg bw	O	no		S
Exposure per day / Chronic Dose	0,000189954	mg/kg bw/day	O	no		S
**Oral**						
Exposure / Dose	accidental		S	accidental		S
**Intake**						
Exposure per task / Acute Dose	0,209666667	mg/kg bw	O	0,436746988	mg/kg bw	S
Exposure per day / Chronic Dose	0,02987032	mg/kg bw/day	O	0,062221489	mg/kg bw/day	O

Post application phase	Task C: residence time					
	Primary exposure	User		Secondary exposure	Bystander	
**Inhalation**						
Concentration of potential exposure	5,333333333	mg/m^3	O	5,333333333	mg/m^3	O
Inhalation	0,074074074	mg/min	O	0,021481481	mg/min	O
Absorption per task	26,66666667	mg/task	O	7,733333333	mg/task	O
Exposure per task / Acute Dose	0,444444444	mg/kg bw	O	0,931726908	mg/kg bw	O
Exposure per day / Chronic Dose	0,063318113	mg/kg bw/day	O	0,132739176	mg/kg bw/day	O
**Dermal**						
Contact area between product and skin	0,042	m^2	S	0,01040125	m^2	S
Quantity of contact (mg)	0,08	mg	S	0,08	mg	S
Dermal load (mg/cm^2)	0,000190476	mg/cm^2	O	0,000769138	mg/cm^2	O
Absorption (mg)	0,08	mg	O	0,08	mg	O
Exposure per task / Acute Dose	0,001333333	mg/kg bw	O	0,009638554	mg/kg bw	O
Exposure per day / Chronic Dose	0,000189954	mg/kg bw/day	O	0,001373164	mg/kg bw/day	O
**Oral**						
Exposure / Dose	accidental		S	accidental		S
**Intake**						
Exposure per task / Acute Dose	0,445777778	mg/kg bw	O	0,941365462	mg/kg bw	O
Exposure per day / Chronic Dose	0,063508067	mg/kg bw/day	O	0,13411234	mg/kg bw/day	O

Disposal	Task D: not applicable					

U = Undefined, D = Default, S = Set, O = Output, REM = Remark, not used in calculation

Scenario 36: insecticide: evaporator, indoor

Scenario description

				Comments
Name of product	electrical evaporator		S	
Physical state product (liquid/solid)	solid		S	
Concentration of active substance in product	1	mg/plate	S	*Frame Formulation*
User	Consumer		S	
Bystander	Children		S	
Temperature	20	°C	D	*room temperature*

Mixing & Loading

	Task A: Loading the evaporator			
Number of tasks per year	150	tasks/yr	S	*ConsExpo 150 tasks/yr*
Duration of task	1	min/task	S	
Quantity of product used per task	1	plate/task	S	*Frame formulation 7 mg/15 m^2 in 8 h*
Quantity of active substance used per task	1	mg/task	O	
Model inhalation exposure	Exposure to vapour / Instantaneous release (limited to vapour pressure)			
Room volume	1	m^3	S	*cloud around user*
Model dermal exposure	Direct dermal contact			
Contact area between product and skin	0,00336	m^2	S	*20% Fingertips (2 Fingers)*
Contact amount between active substance and skin	0,01	mg/task	S	*1% of whole amount used*
Model oral exposure	accidental			

Application

	Task B: using the evaporator			
Number of tasks per year	150	tasks/yr	S	*ConsExpo 150 tasks/yr*
Duration of task	600	min/task	S	*Frame formulation 10 hours, ConsExpo 480 min*
Quantity of active substance used per task	1	mg/task	O	*Frame formulation 7 mg/15 m^2 in 8 h*
Model inhalation exposure	Exposure to vapour / Instantaneous release (limited to vapour pressure)			
Room volume	16	m^3	S	*Sleeping room*
Model dermal exposure	Direct dermal contact			
Contact area between product and skin	0	m^2	S	*no direct contact*
Contact area between product and skin children	0	m^2	S	*no direct contact*
Model oral exposure	accidental			

Post application phase

Task C: not applicable

Disposal

Task D: not applicable

Summary Results Exposure

	User	Consumer	Bystander	Children
Highest potential exposure acute (all amount used is absorbed)	0,016666667	mg/kg bw	0,120481928	mg/kg bw
Highest potential exposure chronic (all amount used is absorbed)	0,006849315	mg/kg bw/day	0,049513121	mg/kg bw/day
Highest potential concentration in air	1	mg/m^3	1	mg/m^3
Inhalation acute	0,006684028	mg/kg bw	0,014012299	mg/kg bw
Inhalation chronic	0,002746861	mg/kg bw/day	0,005758479	mg/kg bw/day
Dermal acute	0,000166667	mg/kg bw	0	mg/kg bw
Dermal chronic	6,84932E-05	mg/kg bw/day	0	mg/kg bw/day
Oral acute	accidental		accidental	
Oral chronic	accidental		accidental	
Intake acute	0,006850694	mg/kg bw	0,014012299	mg/kg bw
Daily intake chronic	0,002815354	mg/kg bw/day	0,005758479	mg/kg bw/day

U = Undefined, D = Default, S = Set, O = Output, REM = Remark, not used in calculation

Scenario 36: insecticide: evaporator, indoor

Results / Output

Mixing & Loading	Task A: Loading the evaporator					
	Primary exposure	User		Secondary exposure	Bystander	
**Inhalation**						
Concentration of potential exposure	1	mg/m^3	O	1	mg/m^3	O
Inhalation	0,013888889	mg/min	O	0,004027778	mg/min	O
Absorption per task	0,010416667	mg/task	O	0,003020833	mg/task	O
Exposure per task / Acute Dose	0,000173611	mg/kg bw	O	0,000363956	mg/kg bw	O
Exposure per day / Chronic Dose	7,1347E-05	mg/kg bw/day	O	0,000149571	mg/kg bw/day	O
**Dermal**						
Contact area between product and skin	0,00336	m^2	S			
Quantity of contact (mg)	0,01	mg	S			
Dermal load (mg/cm^2)	0,000297619	mg/cm^2	O			
Absorption (mg)	0,01	mg	O			
Exposure per task / Acute Dose	0,000166667	mg/kg bw	O	no	mg/kg bw	S
Exposure per day / Chronic Dose	6,84932E-05	mg/kg bw/day	O	no	mg/kg bw/day	S
**Oral**						
Exposure / Dose	accidental		S	accidental		S
**Intake**						
Exposure per task / Acute Dose	0,000340278	mg/kg bw	O	0,000363956	mg/kg bw	O
Exposure per day / Chronic Dose	0,00013984	mg/kg bw/day	O	0,000149571	mg/kg bw/day	O

Application	Task B: using the evaporator					
	Primary exposure	User		Secondary exposure	Bystander	
**Inhalation**						
Concentration of potential exposure	0,0625	mg/m^3	O	0,0625	mg/m^3	O
Inhalation	0,000868056	mg/min	O	0,000251736	mg/min	O
Absorption per task	0,390625	mg/task	O	0,11328125	mg/task	O
Exposure per task / Acute Dose	0,006510417	mg/kg bw	O	0,013648343	mg/kg bw	O
Exposure per day / Chronic Dose	0,002675514	mg/kg bw/day	O	0,005608908	mg/kg bw/day	O
**Dermal**						
Exposure per task / Acute Dose	no	mg/kg bw	S	no	mg/kg bw	S
Exposure per day / Chronic Dose	no	mg/kg bw/day	S	no	mg/kg bw/day	S
**Oral**						
Exposure / Dose	accidental		S	accidental		S
**Intake**						
Exposure per task / Acute Dose	0,006510417	mg/kg bw	O	0,013648343	mg/kg bw	O
Exposure per day / Chronic Dose	0,002675514	mg/kg bw/day	O	0,005608908	mg/kg bw/day	O

Post application phase	Task C: not applicable

Disposal	Task D: not applicable

U = Undefined, D = Default, S = Set, O = Output, REM = Remark, not used in calculation

Scenario 36b: insecticide: evaporator, wardrobe

Scenario description

				Comments
Name of product	strip against moth		S	
Physical state product (liquid/solid)	solid		S	
Concentration of active substance in product	1	mg/strip	S	*Frame formulation*
User	Consumer		S	
Bystander	Children		S	
Temperature	20	°C	D	*room temperature*
**Mixing & Loading**	Task A: placing the strip into the wardrobe			
Number of tasks per year	4	tasks/yr	S	*every three month*
Duration of task	1	min/task	S	
Quantity of product used per task	1	strip/task	S	
Quantity of active substance used per task	1	mg/task	O	
Model inhalation exposure	Exposure to vapour / Instantaneous release (limited to vapour pressure)			
Room volume	1	m^3	S	*cloud around user*
Model dermal exposure	Direct dermal contact			
Contact area between product and skin	0,00336	m^2	S	*20% Fingertips (2 Fingers)*
Contact amount between active substance and skin	0,001	mg/task	S	*0.1% of whole amount used [see HERA Perborate]*
Model oral exposure	accidental			
**Application**	Task B: opening the wardrobe			
Number of tasks per year	365	tasks/yr	S	*once per day*
Duration of task	5	min/task	S	
Quantity of active substance used per task	1	mg/task	O	
Model inhalation exposure	Exposure to vapour / Instantaneous release (limited to vapour pressure)			
Room volume	1	m^3	S	*head in wardrobe (1x2x0.5 m)*
Model dermal exposure	Direct dermal contact			
Contact area between product and skin	0	m^2	S	*no direct contact*
Contact area between product and skin children	0	m^2	S	*no direct contact*
Model oral exposure	accidental			
**Post application phase**	Task C: not applicable			
**Disposal**	Task D: not applicable			

Summary Results Exposure

	**User**		**Consumer**	**Bystander**		**Children**
Highest potential exposure acute (all amount used is absorbed)		0,016666667	mg/kg bw		0,120481928	mg/kg bw
Highest potential exposure chronic (all amount used is absorbed)		0,000182648	mg/kg bw/day		0,00132035	mg/kg bw/day
Highest potential concentration in air		1	mg/m^3		1	mg/m^3
Inhalation acute		0,001041667	mg/kg bw		0,002183735	mg/kg bw
Inhalation chronic		0,000182648	mg/kg bw/day		0,00132035	mg/kg bw/day
Dermal acute		1,66667E-05	mg/kg bw		0	mg/kg bw
Dermal chronic		1,82648E-07	mg/kg bw/day		0	mg/kg bw/day
Oral acute	accidental			accidental		
Oral chronic	accidental			accidental		
Intake acute		0,001058333	mg/kg bw		0,002183735	mg/kg bw
Daily intake chronic		0,000182648	mg/kg bw/day		0,00132035	mg/kg bw/day

U = Undefined, D = Default, S = Set, O = Output, REM = Remark, not used in calculation

Scenario 36b: insecticide: evaporator, wardrobe

Results / Output

Mixing & Loading	Task A: placing the strip into the wardrobe					
	Primary exposure	User		Secondary exposure	Bystander	
**Inhalation**						
Concentration of potential exposure	1	mg/m^3	O	1	mg/m^3	O
Inhalation	0,013888889	mg/min	O	0,004027778	mg/min	O
Absorption per task	0,010416667	mg/task	O	0,003020833	mg/task	O
Exposure per task / Acute Dose	0,000173611	mg/kg bw	O	0,000363956	mg/kg bw	O
Exposure per day / Chronic Dose	1,90259E-06	mg/kg bw/day	O	3,98856E-06	mg/kg bw/day	O
**Dermal**						
Contact area between product and skin	0,00336	m^2	S			
Quantity of contact (mg)	0,001	mg	S			
Dermal load (mg/cm^2)	2,97619E-05	mg/cm^2	O			
Absorption (mg)	0,001	mg	O			
Exposure per task / Acute Dose	1,66667E-05	mg/kg bw	O	no	mg/kg bw	S
Exposure per day / Chronic Dose	1,82648E-07	mg/kg bw/day	O	no	mg/kg bw/day	S
**Oral**						
Exposure / Dose	accidental		S	accidental		S
**Intake**						
Exposure per task / Acute Dose	0,000190278	mg/kg bw	O	0,000363956	mg/kg bw	O
Exposure per day / Chronic Dose	2,08524E-06	mg/kg bw/day	O	3,98856E-06	mg/kg bw/day	O

Application	Task B: opening the wardrobe					
	Primary exposure	User		Secondary exposure	Bystander	
**Inhalation**						
Concentration of potential exposure	1	mg/m^3	O	1	mg/m^3	O
Inhalation	0,013888889	mg/min	O	0,004027778	mg/min	O
Absorption per task	0,052083333	mg/task	O	0,015104167	mg/task	O
Exposure per task / Acute Dose	0,000868056	mg/kg bw	O	0,001819779	mg/kg bw	O
Exposure per day / Chronic Dose	0,000182648	mg/kg bw/day	S	0,00132035	mg/kg bw/day	S
**Dermal**						
Exposure per task / Acute Dose	no	mg/kg bw	S	no	mg/kg bw	S
Exposure per day / Chronic Dose	no	mg/kg bw/day	S	no	mg/kg bw/day	S
**Oral**						
Exposure / Dose	accidental		S	accidental		S
**Intake**						
Exposure per task / Acute Dose	0,000868056	mg/kg bw	O	0,001819779	mg/kg bw	O
Exposure per day / Chronic Dose	0,000182648	mg/kg bw/day	S	0,00132035	mg/kg bw/day	S

Post application phase	Task C: not applicable

Disposal	Task D: not applicable

Scenario 36c: insecticide: evaporator, dustbin

Scenario description

				Comments
Name of product	dispenser for dustbins		S	
Physical state product (liquid/solid)	solid		S	
Concentration of active substance in product	1	mg/dispenser	S	*Frame formulation*
User	Consumer		S	
Bystander	Children		S	
Temperature	20	°C	D	*room temperature*
**Mixing & Loading**	Task A: placing the dispenser into the dustbin			
Number of tasks per year	6	tasks/yr	S	*every two month, frame formulation*
Duration of task	1	min/task	S	
Quantity of product used per task	1	dispenser/task	S	
Quantity of active substance used per task	1	mg/task	O	
Model inhalation exposure	Exposure to vapour / Instantaneous release (limited to vapour pressure)			
Room volume	1	m^3	S	*cloud around user*
Model dermal exposure	Direct dermal contact			
Contact area between product and skin	0,00336	m^2	S	*20% Fingertips (2 Fingers)*
Contact amount between active substance and skin	0,01	mg/task	S	*1% of whole amount used*
Model oral exposure	accidental			
**Application**	Task B: opening the dustbin			
Number of tasks per year	365	tasks/yr	S	*once per day*
Duration of task	5	min/task	S	
Quantity of active substance used per task	1	mg/task	O	
Model inhalation exposure	Exposure to vapour / Instantaneous release (limited to vapour pressure)			
Room volume	1	m^3	S	*cloud around user during opening*
Model dermal exposure	Direct dermal contact			
Contact area between product and skin	0	m^2	S	*no direct contact*
Contact area between product and skin children	0	m^2	S	*no direct contact*
Model oral exposure	accidental			
**Post application phase**	Task C: not applicable			
**Disposal**	Task D: not applicable			

Summary Results Exposure

	User	Consumer		Bystander	Children	
Highest potential exposure acute (all amount used is absorbed)		0,016666667	mg/kg bw		0,120481928	mg/kg bw
Highest potential exposure chronic (all amount used is absorbed)		0,000273973	mg/kg bw/day		0,001980525	mg/kg bw/day
Highest potential concentration in air		1	mg/m^3		1	mg/m^3
Inhalation acute		0,001041667	mg/kg bw		0,002183735	mg/kg bw
Inhalation chronic		0,000273973	mg/kg bw/day		0,001825762	mg/kg bw/day
Dermal acute		0,000166667	mg/kg bw		0	mg/kg bw
Dermal chronic		2,73973E-06	mg/kg bw/day		0	mg/kg bw/day
Oral acute	accidental			accidental		
Oral chronic	accidental			accidental		
Intake acute		0,001208333	mg/kg bw		0,002183735	mg/kg bw
Daily intake chronic		0,000273973	mg/kg bw/day		0,001825762	mg/kg bw/day

U = Undefined, D = Default, S = Set, O = Output, REM = Remark, not used in calculation

Scenario 36c: insecticide: evaporator, dustbin

Results / Output

Mixing & Loading	Task A: placing the dispenser into the dustbin					
	Primary exposure	User		Secondary exposure	Bystander	
**Inhalation**						
Concentration of potential exposure	1	mg/m^3	O	1	mg/m^3	O
Inhalation	0,013888889	mg/min	O	0,004027778	mg/min	O
Absorption per task	0,010416667	mg/task	O	0,003020833	mg/task	O
Exposure per task / Acute Dose	0,000173611	mg/kg bw	O	0,000363956	mg/kg bw	O
Exposure per day / Chronic Dose	2,85388E-06	mg/kg bw/day	O	5,98284E-06	mg/kg bw/day	O
**Dermal**						
Contact area between product and skin	0,00336	m^2	S			
Quantity of contact (mg)	0,01	mg	S			
Dermal load (mg/cm^2)	0,000297619	mg/cm^2	O			
Absorption (mg)	0,01	mg	O			
Exposure per task / Acute Dose	0,000166667	mg/kg bw	O	no	mg/kg bw	S
Exposure per day / Chronic Dose	2,73973E-06	mg/kg bw/day	O	no	mg/kg bw/day	S
**Oral**						
Exposure / Dose	accidental		S	accidental		S
**Intake**						
Exposure per task / Acute Dose	0,000340278	mg/kg bw	O	0,000363956	mg/kg bw	O
Exposure per day / Chronic Dose	5,59361E-06	mg/kg bw/day	O	5,98284E-06	mg/kg bw/day	O

Application	Task B: opening the dustbin					
	Primary exposure	User		Secondary exposure	Bystander	
**Inhalation**						
Concentration of potential exposure	1	mg/m^3	O	1	mg/m^3	O
Inhalation	0,013888889	mg/min	O	0,004027778	mg/min	O
Absorption per task	0,052083333	mg/task	O	0,015104167	mg/task	O
Exposure per task / Acute Dose	0,000868056	mg/kg bw	O	0,001819779	mg/kg bw	O
Exposure per day / Chronic Dose	0,000273973	mg/kg bw/day	S	0,001819779	mg/kg bw/day	O
**Dermal**						
Exposure per task / Acute Dose	no	mg/kg bw	S	no	mg/kg bw	S
Exposure per day / Chronic Dose	no	mg/kg bw/day	S	no	mg/kg bw/day	S
**Oral**						
Exposure / Dose	accidental		S	accidental		S
**Intake**						
Exposure per task / Acute Dose	0,000868056	mg/kg bw	O	0,001819779	mg/kg bw	O
Exposure per day / Chronic Dose	0,000273973	mg/kg bw/day	S	0,001819779	mg/kg bw/day	O

Post application phase	Task C: not applicable

Disposal	Task D: not applicable

Scenario 37: insecticide: strip

Scenario description

				Comments
Name of product	strip against flying insects in living rooms		S	
Physical state product (liquid/solid)	solid		S	
Concentration of active substance in product	1	mg/strip	S	*Frame formulation*
User	Consumer		S	
Bystander	Children		S	
Temperature	20	°C	D	*room temperature*

Mixing & Loading

	Task A: hanging up the strip			
Number of tasks per year	6	tasks/yr	S	*every two month*
Duration of task	1	min/task	S	
Quantity of product used per task	1	strip/task	S	*Frame formulation 1 strip for 20-40 m^3*
Quantity of active substance used per task	1	mg/task	O	
Model inhalation exposure	Exposure to vapour / Instantaneous release (limited to vapour pressure)			
Room volume	1	m^3	S	*cloud around user*
Model dermal exposure	Direct dermal contact			
Contact area between product and skin	0,00336	m^2	S	*20% Fingertips (2 Fingers)*
Contact amount between active substance and skin	0,1	mg/task	S	*0.1% of whole amount used [see HERA Perborate]*
Model oral exposure	accidental			

Application

	Task B: using the strip			
Number of tasks per year	365	tasks/yr	S	*every day*
Duration of task	480	min/task	S	*time in room per day, 8 hours*
Quantity of active substance used per task	1	mg/task	O	
Model inhalation exposure	Exposure to vapour / Instantaneous release (limited to vapour pressure)			
Room volume	15	m^3	S	*Kitchen*
Model dermal exposure	Direct dermal contact			
Contact area between product and skin	0	m^2	S	*no direct contact*
Contact area between product and skin children	0	m^2	S	*no direct contact*
Model oral exposure	accidental			

Post application phase

Task C: not applicable

Disposal

Task D: not applicable

Summary Results Exposure

	User		Consumer	Bystander		Children
Highest potential exposure acute (all amount used is absorbed)		0,016666667	mg/kg bw		0,120481928	mg/kg bw
Highest potential exposure chronic (all amount used is absorbed)		0,000273973	mg/kg bw/day		0,001980525	mg/kg bw/day
Highest potential concentration in air		1	mg/m^3		1	mg/m^3
Inhalation acute		0,005729167	mg/kg bw		0,012010542	mg/kg bw
Inhalation chronic		0,000273973	mg/kg bw/day		0,001980525	mg/kg bw/day
Dermal acute		0,001666667	mg/kg bw		0	mg/kg bw
Dermal chronic		2,73973E-05	mg/kg bw/day		0	mg/kg bw/day
Oral acute	accidental			accidental		
Oral chronic	accidental			accidental		
Intake acute		0,007395833	mg/kg bw		0,012010542	mg/kg bw
Daily intake chronic		0,000273973	mg/kg bw/day		0,001980525	mg/kg bw/day

U = Undefined, D = Default, S = Set, O = Output, REM = Remark, not used in calculation

Scenario 37: insecticide: strip

Results / Output

Mixing & Loading	Task A: hanging up the strip					
	Primary exposure	User		Secondary exposure	Bystander	
**Inhalation**						
Concentration of potential exposure	1	mg/m^3	O	1	mg/m^3	O
Inhalation	0,013888889	mg/min	O	0,004027778	mg/min	O
Absorption per task	0,010416667	mg/task	O	0,003020833	mg/task	O
Exposure per task / Acute Dose	0,000173611	mg/kg bw	O	0,000363956	mg/kg bw	O
Exposure per day / Chronic Dose	2,85388E-06	mg/kg bw/day	O	5,98284E-06	mg/kg bw/day	O
**Dermal**						
Contact area between product and skin	0,00336	m^2	S			
Quantity of contact (mg)	0,1	mg	S			
Dermal load (mg/cm^2)	0,00297619	mg/cm^2	O			
Absorption (mg)	0,1	mg	O			
Exposure per task / Acute Dose	0,001666667	mg/kg bw	O	no	mg/kg bw	S
Exposure per day / Chronic Dose	2,73973E-05	mg/kg bw/day	O	no	mg/kg bw/day	S
**Oral**						
Exposure / Dose	accidental		S	accidental		S
**Intake**						
Exposure per task / Acute Dose	0,001840278	mg/kg bw	O	0,000363956	mg/kg bw	O
Exposure per day / Chronic Dose	3,02511E-05	mg/kg bw/day	O	5,98284E-06	mg/kg bw/day	O

Application	Task B: using the strip					
	Primary exposure	User		Secondary exposure	Bystander	
**Inhalation**						
Concentration of potential exposure	0,066666667	mg/m^3	O	0,066666667	mg/m^3	O
Inhalation	0,000925926	mg/min	O	0,000268519	mg/min	O
Absorption per task	0,333333333	mg/task	O	0,096666667	mg/task	O
Exposure per task / Acute Dose	0,005555556	mg/kg bw	O	0,011646586	mg/kg bw	O
Exposure per day / Chronic Dose	0,000273973	mg/kg bw/day	S	0,001980525	mg/kg bw/day	S
**Dermal**						
Exposure per task / Acute Dose	no	mg/kg bw	S	no	mg/kg bw	S
Exposure per day / Chronic Dose	no	mg/kg bw/day	S	no	mg/kg bw/day	S
**Oral**						
Exposure / Dose	accidental		S	accidental		S
**Intake**						
Exposure per task / Acute Dose	0,005555556	mg/kg bw	O	0,011646586	mg/kg bw	O
Exposure per day / Chronic Dose	0,000273973	mg/kg bw/day	S	0,001980525	mg/kg bw/day	S

Post application phase	Task C: not applicable

Disposal	Task D: not applicable

U = Undefined, D = Default, S = Set, O = Output, REM = Remark, not used in calculation

Scenario 38: insecticide: trap

Scenario description

				Comments
Name of product	trap against ants		S	
Physical state product (liquid/solid)	solid		S	
Concentration of active substance in product	1	%	S	*Frame formulation*
User	Consumer		S	
Bystander	Children		S	
Temperature	20	°C	D	*room temperature*
**Mixing & Loading**	Task A: positioning the trap			
Number of tasks per year	6	tasks/yr	S	*every two month*
Duration of task	1	min/task	S	
Quantity of product used per task	2,9	g/trap/task	S	*Technical Inf. Blattanex*
Quantity of active substance used per task	29	mg/task	S	
Model inhalation exposure	Exposure to vapour / Instantaneous release (limited to vapour pressure)			
Room volume	1	m^3	S	*cloud around user*
Model dermal exposure	Direct dermal contact			
Contact area between product (powder) and skin	0	m^2	S	*no contact*
Model oral exposure	accidental			
**Application**	Task B: using the trap			
Number of tasks per year	365	tasks/yr	S	*every day*
Duration of task	480	min/task	S	*time in room per day, 8 hours*
Quantity of active substance used per task	29	mg/task	S	
Model inhalation exposure	Exposure to vapour / Instantaneous release (limited to vapour pressure)			
Room volume	15	m^3	S	*Kitchen*
Model dermal exposure	Direct dermal contact			
Contact area between product (powder) and skin	0	m^2	S	*no direct contact*
Contact area between product (powder) and skin children	0	m^2	S	*no direct contact*
Model oral exposure	accidental			
**Post application phase**	Task C: not applicable			
**Disposal**	Task D: not applicable			

Summary Results Exposure

	User		Consumer	Bystander		Children
Highest potential exposure acute (all amount used is absorbed)		0,483333333	mg/kg bw		3,493975904	mg/kg bw
Highest potential exposure chronic (all amount used is absorbed)		0,007945205	mg/kg bw/day		0,05743522	mg/kg bw/day
Highest potential concentration in air		29	mg/m^3		29	mg/m^3
Inhalation acute		0,166145833	mg/kg bw		0,348305723	mg/kg bw
Inhalation chronic		0,007945205	mg/kg bw/day		0,053112531	mg/kg bw/day
Dermal acute		0	mg/kg bw		0	mg/kg bw
Dermal chronic		0	mg/kg bw/day		0	mg/kg bw/day
Oral acute	accidental			accidental		
Oral chronic	accidental			accidental		
Intake acute		0,166145833	mg/kg bw		0,348305723	mg/kg bw
Daily intake chronic		0,007945205	mg/kg bw/day		0,053112531	mg/kg bw/day

U = Undefined, D = Default, S = Set, O = Output, REM = Remark, not used in calculation

Scenario 38: insecticide: trap

Results / Output

Mixing & Loading	Task A: positioning the trap					
	**Primary exposure**	**User**		**Secondary exposure**	**Bystander**	
**Inhalation**						
Concentration of potential exposure	29	mg/m^3	O	29	mg/m^3	O
Inhalation	0,402777778	mg/min	O	0,116805556	mg/min	O
Absorption per task	0,302083333	mg/task	O	0,087604167	mg/task	O
Exposure per task / Acute Dose	0,005034722	mg/kg bw	O	0,010554719	mg/kg bw	O
Exposure per day / Chronic Dose	8,27626E-05	mg/kg bw/day	O	0,000173502	mg/kg bw/day	O
**Dermal**						
Exposure per task / Acute Dose	no	mg/kg bw	S	no	mg/kg bw	S
Exposure per day / Chronic Dose	no	mg/kg bw/day	S	no	mg/kg bw/day	S
**Oral**						
Exposure / Dose	accidental		S	accidental		S
**Intake**						
Exposure per task / Acute Dose	0,005034722	mg/kg bw	O	0,010554719	mg/kg bw	O
Exposure per day / Chronic Dose	8,27626E-05	mg/kg bw/day	O	0,000173502	mg/kg bw/day	O

Application	Task B: using the trap					
	**Primary exposure**	**User**		**Secondary exposure**	**Bystander**	
**Inhalation**						
Concentration of potential exposure	1,933333333	mg/m^3	O	1,933333333	mg/m^3	O
Inhalation	0,026851852	mg/min	O	0,007787037	mg/min	O
Absorption per task	9,666666667	mg/task	O	2,803333333	mg/task	O
Exposure per task / Acute Dose	0,161111111	mg/kg bw	O	0,337751004	mg/kg bw	O
Exposure per day / Chronic Dose	0,007945205	mg/kg bw/day	S	0,052939029	mg/kg bw/day	S
**Dermal**						
Exposure per task / Acute Dose	no	mg/kg bw	S	no	mg/kg bw	S
Exposure per day / Chronic Dose	no	mg/kg bw/day	S	no	mg/kg bw/day	S
**Oral**						
Exposure / Dose	accidental		S	accidental		S
**Intake**						
Exposure per task / Acute Dose	0,161111111	mg/kg bw	O	0,337751004	mg/kg bw	O
Exposure per day / Chronic Dose	0,007945205	mg/kg bw/day	S	0,052939029	mg/kg bw/day	S

Post application phase	Task C: not applicable

Disposal	Task D: not applicable

U = Undefined, D = Default, S = Set, O = Output, REM = Remark, not used in calculation
